# Improving pharmacogenetic prediction of extrapyramidal symptoms induced by antipsychotics

**DOI:** 10.1038/s41398-018-0330-4

**Published:** 2018-12-13

**Authors:** Daniel Boloc, Anna Gortat, Jia Qi Cheng-Zhang, Susana García-Cerro, Natalia Rodríguez, Mara Parellada, Jeronimo Saiz-Ruiz, Manolo J. Cuesta, Patricia Gassó, Amalia Lafuente, Miquel Bernardo, Sergi Mas

**Affiliations:** 1 Department of Medicine, University of Barcelona, Barcelona, Spain; 2 Department of Clinical Foundations, Pharmacology Unit, University of Barcelona, Barcelona, Spain; 3 Centro de Investigación Biomédica en Red de Salud Mental (CIBERSAM), Carlos III Health Institute, Barcelona, Spain; 4 Child and Adolescent Psychiatry Department, Hospital General Universitario Gregorio Marañón, School of Medicine, Universidad Complutense, IiSGM, Madrid, Spain; 5 Hospital Ramon y Cajal, Universidad de Alcala, IRYCIS, Madrid, Spain; 6 Department of Psychiatry, Complejo Hospitalario de Navarra, Instituto de Investigación Sanitaria de Navarra (IdiSNA), Pamplona, Spain; 7 The August Pi i Sunyer Biomedical Research Institute (IDIBAPS), Barcelona, Spain; 8 Barcelona Clínic Schizophrenia Unit, Hospital Clínic de Barcelona, Barcelona, Spain

**Keywords:** Personalized medicine, Pharmacogenomics

## Abstract

In previous work we developed a pharmacogenetic predictor of antipsychotic (AP) induced extrapyramidal symptoms (EPS) based on four genes involved in mTOR regulation. The main objective is to improve this predictor by increasing its biological plausibility and replication. We re-sequence the four genes using next-generation sequencing. We predict functionality “in silico” of all identified SNPs and test it using gene reporter assays. Using functional SNPs, we develop a new predictor utilizing machine learning algorithms (Discovery Cohort, *N* = 131) and replicate it in two independent cohorts (Replication Cohort 1, *N* = 113; Replication Cohort 2, *N* = 113). After prioritization, four SNPs were used to develop the pharmacogenetic predictor of AP-induced EPS. The model constructed using the Naive Bayes algorithm achieved a 66% of accuracy in the Discovery Cohort, and similar performances in the replication cohorts. The result is an improved pharmacogenetic predictor of AP-induced EPS, which is more robust and generalizable than the original.

## Introduction

Antipsychotic (AP) medication is the gold standard in schizophrenia treatment. Although APs have demonstrated overall efficacy and safety there are large inter-individual differences in their efficacy and side effects between patients. Nowadays, treatment selection remains a “trial and error” process, with multiple failed trials required before an acceptable balance between response to therapy and side effects is reached. Finding this balance is especially important considering there is an estimated noncompliance rate of 40% to AP treatment^1^. One of the strongest predictors of noncompliance is the experience of harmful side effects^2^. Therefore, the identification of robust predictors of AP-induced side effects holds the potential to provide a rational basis for treatment selection^3^.

Taking into account that much of the inter-individual variability in AP-induced side effects is due to genetic factors (estimated heritability, *h*^2^, 0.60–0.80), a number of pharmacogenetic markers have been associated with AP side effects, although none is yet a definitive predictor of response^4^.

Acute extrapyramidal symptoms (EPS) induced by AP treatment, which may develop within a few days of initiating the treatment (in contrast to tardive dyskinesia, the late-onset EPS), are frequent and serious adverse reactions to AP drugs. Acute EPS constitutes a complex phenotype including several syndromes: akathisia; acute dystonia; and parkinsonism. Acute dystonia and parkinsonism respond to AP dose reduction and anticholinergic agents, whereas, akathisia does not respond to anticholinergic medication. Even though the exact mechanism underlying each of these different syndromes is not clear, excessive striatal dopamine D2 receptor (DRD2) blockade is believed to be the common cause^5^. Our understanding of the mechanism and the genetic factors accounting for AP-induced EPS is still evolving^6^. In previous studies, our group developed a convergent functional genomics (CFG) approach to identify candidate genes for pharmacogenetic studies of EPS^7–9^. That strategy resulted in the identification of the mTOR pathway as a source of new candidate genes.

Recently, various authors have implicated the mTOR signaling pathway in the mechanism of action of APs^10^. Moreover, the relationship between mTOR and motor alterations has also been observed in Parkinson disease. L-dopa induced dyskinesia appears to be caused by DRD1 hypersentitivity and mTOR pathway activation, and could be attenuated by rapamycin, a potent mTOR inhibitor^11^. In agreement with the L-dopa model, we describe, in mice, that inhibition of mTOR signaling in the striatonigral DRD1 pathway is a possible mechanism underlying the resistance to EPS^12^.

We developed a pharmacogenetic predictor based on four single nucleotide polymorphisms (SNPs), our hypothesis was that genetic variants that modify the mTOR pathway might determine susceptibility to the appearance of AP-induced EPS^13^. However, some aspects of this pharmacogenetic predictor need to be improved before it is ready for clinical application.

The main objective of the present study is to improve the pharmacogenetic predictor of AP-induced EPS based on the mTOR pathway by increasing its biological plausibility and replication in independent populations. To this end: (1) the genes included in the predictor (*AKT1, FCHSD1, RPTOR,* and *DDIT4*) have been re-sequenced using targeted next-generation sequencing (NGS); (2) the functionality of the SNPs identified in each gene has been predicted “in silico” using a web-based software developed to this end by our group; (3) candidate SNPs with suspected functionality have been tested in vitro using luciferase reporter assays; (4) functional candidate SNPs have been used to develop a new predictor of AP-induced EPS utilizing several machine learning algorithms; and (5) the algorithm thus constructed has been replicated in two independent cohorts.

## Material and methods

### Subjects

#### Discovery Sample

Hundred and thirty-one inpatients treated with risperidone (48 cases presenting EPS and 83 controls not presenting EPS) recruited consecutively at the Psychiatry Service of the Hospital Clínic (Barcelona, Spain) over a period of 3 years (2002–2004) who participated in the original study as a Discovery Sample^13^. A complete description of this cohort can be found elsewhere^14,15^.

#### Replication Sample 1

Hundred and thirteen inpatients (49 cases presenting EPS and 64 controls not presenting EPS) recruited from the same Psychiatry Service of the Hospital Clínic (Barcelona, Spain) over a different period of time (2007–2009) treated with risperidone or other APs with similar DRD2 blockade potency and similar risks of inducing EPS (amisulpride, paliperidone, and ziprasidone).

#### Replication Sample 2

Hundred and thirteen patients (43 cases with EPS and 70 controls without) from the PEPs study (*Phenotype–genotype and environmental interaction: application of a predictive model in first psychotic episodes*) treated with the same APs as in Replication Sample 1 (amisulpride, paliperidone, risperidone, and ziprasidone)^16^. The complete clinical protocol used in the PEPs project was previously published elsewhere^17^.

The study was approved by the Ethics Committee of the Hospital Clínic.

### EPS assessment

In order to assess adverse drug reactions in detail, two procedures were followed: (a) identification of EPS events in clinical records; (b) application of the Simpson-Angus scale (SAS)^18^. In accordance with our previous studies^14,17,19,20^, EPS were considered present when three or more items from the SAS were reported in the clinical record. Patients without EPS (SAS < 3 or no-EPS event during the observational period) were taken as controls. The observational period to capture acute EPS was 15 days for the Discovery Sample and Replication Sample 1^14,19,20^. For Replication Sample 2 the observational period was 6 months^17^.

### Targeted next-generation sequencing (NGS)

Eighty-eight samples from the Discovery cohort were sent to IMGM laboratories (Lachhamer, Germany) for sequencing using the Illumina MiSeq platform. The technique was applied to the four genes from the original predictor^13^ with 10 kbps of additional flanking, downstream and upstream.

### Variant calling

From the *.fastq* format generated in the sequencing step, we reconfigured to *.sam* and *.bam* formats with *samtools* (http://samtools.sourceforge.net/). We used the *bowtie2* program (http://bowtie-bio.sourceforge.net/bowtie2/index.shtml) to prepare and index the reference sequence (GRCh37/hg19), to sort the *.bam* files by position, to align the sequences with the reference and finally, to merge and index the alignments.

Using *samtools* and *bcftools* (https://samtools.github.io/bcftools/), we performed the variant calling (filters: depth, quality, and strand bias). In order to map the variants for a specific position/SNP for each patient sequenced, we used custom made *Perl* scripts.

We performed a functionality study of all the SNPs using Ensembl’s Variant Effect Predictor (http://www.ensembl.org/info/docs/tools/vep/index.html), PolyPhen (http://genetics.bwh.harvard.edu/pph2/), PROVEAN (http://provean.jcvi.org/index.php), and SIFT (http://sift.jcvi.org/).

### SNP mapping

SNPs were mapped with the help of a local utility developed in our lab (which is now freely-available through a web portal; SiNoPsis: https://compgen.bio.ub.edu/SiNoPsis)^21^. This utility works with different databases that contain information on cis regulatory elements (CRE). This analysis yields a table classifying each SNP into one of the following categories: ecreSNP (disrupts CRE and is eQTL), creSNP (disrupts CRE, not eQTL), eSNP (only eQTL), and normSNP (neither eQTL nor disrupts CRE).

### SNP selection and genotyping

In order to select candidate SNPs to test their functionality in vitro and to create the predictor, we considered (Supplementary Table s1): (1) SiNoPsis categories; (2) LD with the SNP from the original predictor; and (3) *p*-values from the preliminary association test for EPS (*N* = 88) using SNPassoc R package^22^.

### “In vitro” functionality assessment

#### Construction of promoter–reporter plasmids

We synthesized the DNA fragments using genomic DNA from patients carrying either the wild-type (allele 1) or mutant (allele 2) sequence for each SNP studied. Regions were amplified using OneTaq Polymerase (NEBiolabs, Ipswich, MA, USA) and a pair of primers (Integrated DNA Technologies, Coralville, IA, USA) designed for each sequence (Supplementary Data Table S2). The resulting PCR products were digested with specific restriction enzymes (NEBiolabs) and were cloned into the pGL4.10-basic vector (Promega, Madison, WI, USA). The constructs were all confirmed by DNA sequencing.

#### Cell culture and plasmid transient transfection

The human embryonic kidney 293 (HEK293) cell line (generously donated by Dr. C. Sindreu) was used for the luciferase reporter assay. 2.5 × 10^5^ HEK293 cells were transfected with either 100 or 250 ng of equimolar quantities of each constructed vector or *CMV* as a positive control using the calcium/phosphate method. Cells were separately transfected with the normalization control vector (empty pGL4.10), paired for each test transfection.

#### Luciferase reporter assay

24 h after transfection, cell lysates were incubated with Beetle Lysis Juice (AttendBio, Barcelona, Spain) and the luciferase activity was measured in a Spark® luminometer (TECAN, Männedorf, Switzerland). Measured activities were normalized using empty pGL4.10 as control vector. At least three independent transfection experiments were performed and each luciferase assay was carried out in triplicate.

### Statistical analysis

All the statistical analysis was performed using GraphPad Prism v.6 software (GraphPad Software, La Jolla, CA, USA). Means and standard deviations were computed for continuous variables. The normality of continuous variables was tested according to the Kolmogorov–Smirnov and Shapiro–Wilk tests, and the equality of the variance between groups was assessed by Levene’s test. Student’s *t*-test was used to assess differences between allele 1 and allele 2 in each SNP. In all instances, a value of 0.05 was accepted.

### Development of AP-induced EPS predictor using machine learning

The polymorphisms selected were genotyped in the three populations participating in the present study by real-time PCR using TaqMan allelic discrimination pre-designed assays (Applied Biosystems, Foster City, CA, USA).

In the present analysis, supervised methods of class prediction based on machine learning were applied. This means that the machine is trained to identify classification patterns of controls and cases, using the Discovery Sample. In this process, the software has all the available data for each individual included in the study: the selected genetic markers and the individual’s classification as control or case. The algorithm created by this approach is then validated with the Replication Sample 1 and Replication Sample 2. For this validation, the software only has each individual’s genetic information, and predicts its case or control status according to the algorithm developed, but blind to the individual’s real status.

First, in order to prioritize SNPs, we performed a genetic association analysis of EPS with the selected SNPs, in the Discovery Sample using the SNPassoc R package^22^. SNPs with the nominal significant *p*-values were selected. Then, classification algorithms were applied in the Discovery Sample. For each algorithm, we used 10-fold cross-validation to estimate the prediction error. The best model was selected and then validated using Replication Sample 1 and Replication Sample 2.

We evaluated the performance of the different classification techniques using: (1) area under the curve (AUC), for classification model comparison; (2) sensitivity (true positives (TP))/((TP+false negatives (FN)), the capacity to predict EPS cases correctly; (3) specificity (true negative (TN))/(TN+FN), the capacity to reject non-EPS controls); (4) accuracy (TP+TN)/All, the capacity to correctly predict EPS cases and non-EPS controls; (5) positive predictive value (PPV) TP/(TP+FP), measures the EPS cases predicted correctly; (6) positive likelihood ratio test (LR+) (sensitivity/1 − specificity), to assess the value of performing a prediction; and (7) The Matthews correlation coefficient (MCC) a measure of the quality of binary (two-class) prediction.

We used three machine learning methods^23–25^ from the free open-source software package Orange v.2.7 (http://orange.biolab.si/download/):

- *Support Vector Machine (SVM)*: RBF kernels were used. We used the Automatic Parameter Search that tunes the relevant SVM parameters in a methodologically sound manner. All other parameters were set to default.

- *Naive Bayes (NB)*: Laplace estimate was used for assessing prior class probabilities; the method for estimating conditional probabilities was the m-estimate; and the parameter for m-estimate was set to 2.0.

- *Random Forest (RF)*: We grew trees without any pre-pruning. Ten classification trees were included in the forest. The number of attributes that are arbitrarily drawn for consideration at each node number was set according to default parameters.

## Results

Demographic and pharmacological data for the three cohorts included in the present study are summarized in Table 1. As expected from the sample description, significant differences in the AP type between the cohorts was observed.

**Table 1:** Demographic and pharmacological data of the three cohorts included in the present study

	Discovery Cohort		Replication Cohort 1		Replication Cohort 2	
	No-EPS	EPS	No-EPS	EPS	No-EPS	EPS
*N*	83	48	64	49	70	43
Gender, male (%)	45 (54.2)	28 (58.3)	38 (59.4)	28 (57.1)	46 (65.7)	32 (74.4)
Age, mean (SD)	35.2 (14.8)	29.4 (12.9)	33.1 (12.9)	31.8 (11.9)	24.4 (6.6)	21.9 (6.1)
Antipsychotic^a^						
Amisulpride, *N* (%)	–	–	4 (6.2)	5 (10.2)	6 (8.6)	1 (2.3)
Paliperidone, *N* (%)	–	–	15 (23.4)	10 (20.4)	17 (24.3)	6 (13.9)
Risperidone, *N* (%)	83 (100.0)	48 (100)	24 (37.5)	26 (53.06)	42 (60.0)	30 (69.7)
Risperidone LAI, *N* (%)	–	–	6 (9.3)	7 (14.2)	4 (5.7)	6 (13.9)
Ziprasidone, *N* (%)	–	–	10 (15.6)	7 (14.2)	1 (1.4)	0 (0.0)
Antipsychotic dose, mean (SD)^b^	362.9 (198.3)	470.7 (211.5)	466.1 (426.1)	443.2 (331.3)	625.2 (464.2)	756.8 (452.2)
Antipsychotic combination, *N* (%)	28 (33.7)	18 (37.5)	25 (37.3)	18 (36.7)	27 (38.5)	17 (39.5)

*SD* standard deviation, *LAI* long acting injection, *EPS* extrapyramidal symptoms

^a^For those patients treated with an AP combination, the AP with the higher CEDD value is listed

^b^For patients treated with an AP combination, the sum of the CEDD of each AP in the combination is calculated

### Targeted next-generation sequencing and SNP mapping

Table 2 shows the result of the NGS for each gene. As it can be observed, only 1.5% of all the SNPs were located in exonic regions of the candidate genes, and only 0.4% could be classified as missense variants. However, according to the Polyphen and SIFT predictions, amino acid changes introduced by SNPs have a weak effect on protein structure or are not potentially harmful. Conversely, 83.5% of SNPs in functionally relevant areas of the gene are located in cis regulatory regions (CRE), including promoters and enhancers.

**Table 2:** Summary of the SNPs identified after resequencing the four candidate genes in 88 samples of the Discovery cohort

			SNP functionality^b^					SiNoPsis categories^c^		
Gene	SNPs	Fragment size (pb)^a^	UTR	Splicing	Missense	Synonymous	Regulatory region	ecreSNP	creSNP	eSNP
*AKT1*	92	46,395	6	0	2	3	25	9	15	22
*FCHSD1*	61	32,118	10	1	3	3	35	1	5	29
*DDIT4*	34	22,121	2	0	0	0	19	5	1	17
*Raptor*	1455	441,549	11	1	2	12	28	59	53	382

^a^Sequenced DNA including the whole gene and 10 kbp of additional flanking region at downstream and upstream

^b^The functionality was done using Ensembl’s Variant Effect Predictor, PolyPhen, PROVEAN, and SIFT

^c^ecreSNP (disrupts CRE and is eQTL), creSNP (disrupts CRE, not an eQTL), eSNP (only an eQTL)

In order to clarify the potential role of these variants in regulatory regions we developed the SiNoPsis web-based open-source software^21^. Table 2 also includes a summary of this analysis for each gene.

According to: (1) the SiNoPsis classification (ecreSNP > creSNP > eSNP); (2) the LD with the SNPs in the original predictor (higher LD values) and; (3) the result of the preliminary association analysis (lower *p*-value) (Supplementary Table S1), 12 SNPs were selected for in vitro functionality tests and to develop the AP-induced EPS pharmacogenetic predictor (Table 3).

**Table 3:** Selected SNPs to test “in vitro” functionality and to develop the AP-induced EPS pharmacogenetic predictor

Gene	SNP	A1	A2	ID^a^	Location^b^	MAF^c^	HWE^c^	*p*-value^c,d^	LD^c, e^	SiNoPsis	MAF^f^	HWE^f^	Cod^f^	Dom^f^	Rec^f^	Over^f^	Add^f^
*AKT1*	rs1130214	G	T	A1	14:105259500–105261800	0.34	0.05	0.002	1.00	creSNP	0.33	0.308	**0.04**	0.147	0.136	**0.02**	0.654
	rs74090038	G	A	A2	14:105262088–105263088	0.32	0.06	0.028	0.83	creSNP	0.32	0.315	0.122	0.325	0.135	0.810	0.101
	rs67583154	C	T	A3	14:105265588–105267500	0.12	0.34	0.635	0.38	ecreSNP	0.15	0.436	0.305	0.317	0.335	0.206	0.480
	rs33925946	C	A	A4	14:105271000–105272088	0.30	0.07	0.032	0.67	creSNP	0.30	0.357	**0.01**	0.147	**0.05**	**0.01**	0.730
*FCHSD1*	rs1421896	A	C	F1	5:141015900–141017000	0.41	0.268	0.884	0.35	ecreSNP	0.37	0.889	0.822	0.830	0.624	0.591	0.931
	rs34798770	C	T	F2	5:141036800–141038500	0.34	0.829	0.246	0.25	creSNP	0.44	0.795	0.818	0.628	0.898	0.582	0.808
*DDIT4*	rs1053639	T	A	D1	10:74034943–74035797	0.37	0.492	0.002	1.00	ecreSNP	0.41	0.894	0.381	0.984	0.189	0.328	0.470
	rs4747241	C	T	D2	10:74035797–74036678	0.38	0.364	0.004	0.64	ecreSNP	0.42	0.594	0.414	0.715	0.286	0.235	0.731
	rs4747242	A	C	D3	10:74036797–74037678	0.37	0.492	0.002	0.92	ecreSNP	0.40	0.596	0.386	0.847	0.177	0.401	0.392
	rs10823911	A	C	D4	10:74039870–74040480	0.18	0.821	0.008	0.92	eSNP	0.40	0.689	0.413	0.801	0.189	0.452	0.380
*Raptor*	rs34726568	T	TA	R1	17:78518000–78521100	0.16	1	0.002	0.07	ecreSNP	0.21	0.558	**0.03**	0.184	**0.01**	0.752	**0.05**
	rs9899898	G	A	R2	17:78578583–78580283	0.34	0.06	0.026	0.48	ecreSNP	0.33	0.04	0.665	0.385	0.654	0.539	0.371
	rs9915667	A	G	R3	17:78753340–78756340	0.41	0.246	0.002	0.19	ecreSNP	0.45	0.516	**0.05**	0.04	**0.04**	0.799	**0.01**

*MAF* minimum allele frequency, *HWE* Hardy–Weinberg equilibrium, *LD* linkage disequilibrium, *Cod* codominant, *Dom* dominant, *Rec* recessive, *Over* overdominant, *Add* logg-additive

Summary of the genetic association analysis of AP-induced EPS performed in the Discovery cohort (*N* = 131). The *p*-value of each inheritance model is show. Each model was adjust by age, sex and dosage. In bold, nominal significant *p*-values are shown

^a^Region code identifier used throughout the manuscript

^b^Location of the fragment cloned upstream of the *Luc2P* gene according to GRCh37/hg19

^c^Calculated with the 88 samples of the Discovery cohort that where re-sequenced

^d^*p*-value of the best model (codominant, dominant, recessive, overdominant, log-additive) in the preliminary association test

^e^LD with the variant of the original predictor for each gene

^f^Calculated with the Discovery cohort (*N* = 131)

### In vitro functionality assessment

Variants of the regulatory regions cloned and their localization and identifiers are all specified in Table 3.

**Fig. 1:**
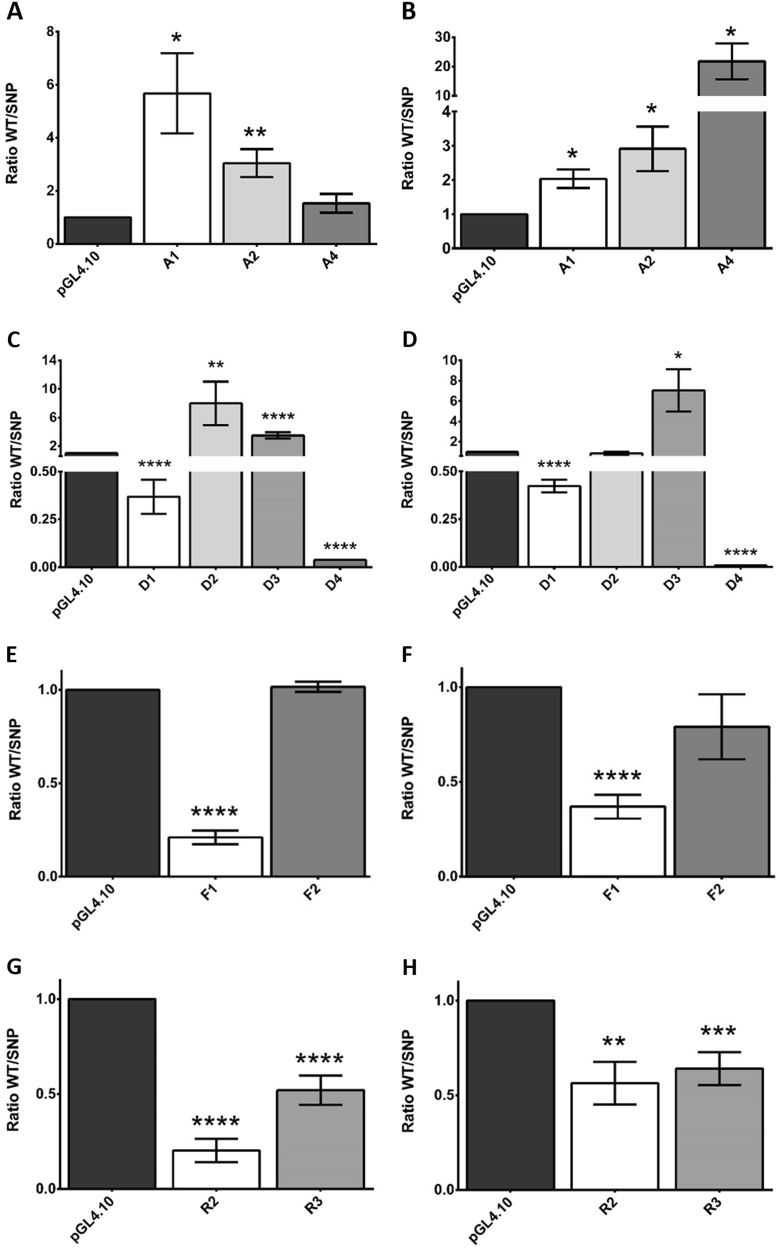
Results of the luciferase assay. using both 100 ng (**a**, **c**, **e**, **g**) and 250 ng (**b**, **d**, **f**, **h**) of DNA, of the selected SNPs in each gene: *AKT1* (**a**, **b**), *DDIT4* (**c**, **d**), *FCHSD1* (**e**, **f**), and *RPTOR* (**g**, **h**). For each SNP the ratio of the activity measured with the Allele 1 vs. Allele 2 is showed. Measured activities were normalized using empty pGL4.10 as a control vector. At least three independent transfection experiments were performed and each luciferase assay was carried out in triplicates. *p-value < 0.05; **p-value < 0.01, ***p-value < 0.001, ****p-value < 0.0001

rs1130214 (A1 region) lies within the first exon of the *AKT1* gene corresponding to the promoter and it includes the TATA box. rs74090038 (A2 region), rs67583154 (A3) and rs33925946 (A4) are three promoter-flanking regions with unknown transcriptional activity (Supplementary Figures S1A) rs67583154 (A3 region) was not assessed due to persistent difficulties in mutant generation. The SNP variants in all three regions were significantly less active than their wild-type variants (Fig. 1a, b).

Four regions within the *DDIT4* gene were studied (Supplementary Figures S1B): D1 (rs1053639) encompasses two thirds of the coding sequence; D2 (rs4747241) is located immediately following the 3′ untranslated region (UTR); while D3 (rs4747242), and D4 (rs10823911), both lie further away from the 3′ UTR of the gene. Both the D1 SNP and D4 SNP had a stimulating effect on transcription; while the transcriptional activities of the D2 SNP and D3 SNP were reduced compared to their wild-type counterparts (Fig. 1c, d).

Two regions within the *FCHSD1* gene were assessed: F1 (rs1421896) within its 3′ UTR, and F2 (rs34798770) which is localized within the 5′ UTR (Supplementary Figures S1C). Although localized within the *FCHSD1* gene, F1, bearing the TATA box, is a part of a vast sequence that overlaps with the histone deacetylase 3 (*HDAC3*) gene and acts as its promoter. The F1 SNP had a stimulating effect on the whole region. In contrast, F2 had almost null activity and was not affected by the introduction of the SNP (Fig. 1e, f).

We assessed two of the three regions within the *RPTOR* gene. The R1 (rs34726568) region was impossible to clone due to persistent amplification difficulties. The R2 (rs9899898) and R3 (rs9915667) regions have characteristics of promoter-flanking regions (Supplementary Figures S1D). The two SNPs significantly enhanced transcriptional activity (Fig. 1g, h).

### Development of AP-induced EPS predictor using machine learning

To build the AP-induced EPS predictor, we first prioritized the selected SNPs based on the association analysis performed on the Discovery Sample (Table 3). Two SNPs in the *AKT1* gene (rs33925946 and rs1130214) and two SNPs in the *RPTOR* gene (rs3476568 and rs9915667) provided nominally significant results and were selected to be included in the predictor.

Three algorithms (Random Forest, Support Vector Machine, and Naive Bayes) were applied to the Discovery Sample (Table 4). The three classifiers provide better prediction than chance, and the Naive Bayes learner achieved the best results in all the parameters used to evaluate the performance of the classification techniques. The Naive Bayes algorithm was used to predict the EPS status of Replication Sample 1 and Replication Sample 2. As it can be observed in Table 4, the different estimated parameters showed similar results for the two replication cohorts.

**Table 4:** Summary of the prediction performance during the training phase (CV = 10) with the Discovery cohort (*N* = 131), and during the replication phase of the best model with both Replication Cohort 1 (*N* = 113) and Replication Cohort 2 (*N* = 113)

Sample	Type	Model	Accuracy	Sensitivity	Specificity	PPV	AUC	LR+	MCC
Discovery cohort	Training	SVM	0.63	0.19	0.89	0.50	0.36	1.73	0.11
	Training	RF	0.65	0.25	0.88	0.55	0.65	2.08	0.17
	Training	NB	0.66	0.31	0.86	0.56	0.64	2.16	0.20
Replication Cohort 1	Replication	NB	0.63	0.39	0.81	0.61	0.64	2.07	0.22
Replication Cohort 2	Replication	NB	0.64	0.38	0.79	0.50	0.58	1.75	0.17

S*VM* support vector machine, *RF* random forest, *NB* naive bayes, *PPV* positive predictive value, *AUC* area under de curve, *LR+* positive likelihood ratio test, *MCC* The Matthews correlation coefficient

## Discussion

In the present study, we refined and replicated a pharmacogenetic predictor of EPS induced by AP. The starting point was an algorithm that had previously been developed by our group, based on the statistical interaction of the genotypes of four SNPs located in four genes involved in the mTOR pathway^13^.

Candidate gene studies have been the gold standard in pharmacogenetics, in part because of the difficulty to recruit enough samples to have sufficient statistical power to perform GWA studies. Regardless of the strategy, most studies use indirect associations. That is, they use marker SNPs (or tagSNPs) that are highly informative with regard to the variability in a gene, and that could be in LD with one or more functional variants. However, after a significant association with a tagSNP, follow-up studies have rarely been performed to identify the functional variant responsible for the association and its possible effect on the transcription of the gene or the functionality of the resulting protein. Moreover, genetic heterogeneity, i.e., more than one SNP in the same gene may be associated with a trait, is rarely taken into account.

In the original predictor, the SNPs selected were not apparently functional, although they had been associated with different clinical phenotypes in other studies^13^. Therefore, in the present study, the four genes and the adjacent regions were sequenced, to identify the functional variants. The results demonstrate something that is not surprising, and that is in fact well known: no variant in the exonic regions of these genes that could induce a change in the amino acid sequence of the resulting protein seems to be related to the presence of EPS. Across many phenotypes, the majority of associated SNPs reside within noncoding regions^26^. Noncoding risk loci are involved in the regulation of transcriptional activity and are enriched in eQTLs and CREs^27^. CREs include promoters and enhancers as well as noncoding sequences, either near to or far from genes, which include binding sites for the regulatory factors required for the expression of the gene. Our hypotheses is that SNPs affecting CREs may alter the proper spatiotemporal organization of the transcriptome in response to AP treatment, and may therefore be associated with AP-induced EPS. Using SiNoPsis^21^ we identified those SNPs that could potentially be modifying CRE functions. This information was crossed with two types of data: from the LD relationship with the variants from the original predictor, and with the information from the statistical association of the SNP with AP-induced EPS.

Since the functionality of the selected SNPs was based on in silico prediction, we decided to test their functionality using an in vitro model. Nine of the ten SNPs tested proved to be functional, since the transcription of the reporter gene was modified by the different alleles of each SNP. This result validates the predictions of SiNoPsis. Since the model does not use a neuronal or CNS-derived cell line, the exact effect that each allele may have on gene transcription is unknown. However, we are certain that the presence of one or another allele in the CRE sequence alters the binding of transcription factors and the establishment of the transcriptional machinery. Using functional SNPs instead of tagSNPs increases the likelihood of replicating the results, since LD differences between populations are avoided. It also increases the biological plausibility of the association.

Another aspect that limits the introduction of pharmacogenetics into clinical practice is that most of the results have their origin in single SNP genetic association studies that ignore the complexity of the relationship between genetic variants created by epistasis^28^. Acute EPS constitutes a complex phenotype^29^, and this complexity does not seem to be explained either by the simple interaction between APs and DRD2^30^ or by the presence of a single genetic variant with a major effect. It is rather due to the presence of multiple SNPs with discrete effects and low penetrance that interact between them. Supervised machine learning methods can detect interactions in the absence of significant individual effects that would be undetectable using traditional methods focused on major differences at the group level. Moreover, supervised machine learning methods characterize the risk at the individual level and not at the population level, in contrast to association methods, thus yielding potentially clinically useful results^31–33^. Although machine learning has some advantages over classical statistics, it also has some limitations that need to be considered, such as overfitting, the effect of genetic heterogeneity, the lack of standardized procedures and the difficulty of interpreting data^34^.

The original predictor was developed using multifactor dimensionality reduction (MDR): the first machine learning methods developed to detect gene–gene interactions^35^. However, some disadvantages have to be considered when using MDR: the models could be difficult to interpret, and the genotype combinations are classified as high or low risk but there is no quantitative measurement of that risk^36^. Therefore, in this study, different algorithms were used that can improve not only the capacity to predict AP-induced EPS but also the replication of the results.

The lack of replication is another reason for the poor clinical translation of pharmacogenetics. Several studies show that the results of the first study correlate only modestly with subsequent research on the same association^37^. The first study often suggests a stronger genetic effect than it is found by subsequent studies. Both bias and genuine population diversity may explain why early association studies tend to overestimate the trait protection or predisposition conferred by a genetic polymorphism. The heterogeneity of phenotypes and differences in LD between populations may also explain the problems in replicating these studies.

We used two different cohorts to replicate the predictor. One of those cohorts was very close to the cohort used to create the predictor, since it was recruited at the same hospital, therefore patients are from the same geographical area and the same group of clinicians established the phenotype. The second cohort came from a multicenter study, where, although the phenotype was established in a similar way, it happened at different centers with different clinicians. This cohort differed in some factors that have been related to EPS risk, including clinical (chronic inpatients vs. first episode of psychosis), pharmacological (different AP types and dosage), and demographical (age and sex) factors^29^. However, the results of applying the predictor in the three populations gave very similar results, showing that the improved predictor is a robust tool capable of correctly classifying a large majority of patients, regardless of the cohort to which they belong. In summary, in this study we optimized the predictor of AP-induced EPS based on the genetic variability of the mTOR pathway.

This new predictor includes four polymorphisms in only two genes: *AKT1* and *RPTOR*. However, the effect of the genes that were no longer included (*DDIT4*, *FCHSD1*) could not be ruled out, although we would need a larger sample with sufficient statistical power to test the effect on the model of other variants. In this sense, it should be noted that the SNP mapped to the *FCHSD1* gene appears to be found in the promoter region of an important gene for epigenetic regulation: *HDCA3*. Further studies are needed to establish the role of this gene in AP-induced EPS, and its possible interaction with the mTOR pathway.

The final result is a predictor with less accuracy than the original but which is more robust and generalizable. This is mainly due to the fact that this algorithm uses functional SNPs instead of SNP markers in LD with causal variants. In addition, the functionality of these SNPs has been tested in vitro. The different parameters used to measure the predictive capacity of this algorithm show that it is at the border of clinical application, since they show moderate to important results. It is essential to continue the search for new candidate genes and research to identify functional SNPs, and thereby to add new variables to the algorithm to increase its predictive capacity. Likewise, clinical, demographic and pharmacological variables should also form part of a future predictor with clinical applicability. A predictor of EPS would be useful for guiding clinicians in their choice of AP, and should reduce the number of unnecessary trials and limit misdiagnosed EPS. For the patient, this will mean fewer adverse events and better compliance, with the overall economic benefits that this implies.

## Supplementary information

Supplementary Figure1A

Supplementary Figure1B

Supplementary Figure1C

Supplementary Table 1 and 2

Supplementary Figure 1D

Supplementary Figures
