# Author Correction: Improving pharmacogenetic prediction of extrapyramidal symptoms induced by antipsychotics

**DOI:** 10.1038/s41398-019-0480-z

**Published:** 2019-05-02

**Authors:** Daniel Boloc, Anna Gortat, Jia Qi Cheng-Zhang, Susana García-Cerro, Natalia Rodríguez, Mara Parellada, Jeronimo Saiz-Ruiz, Manolo J. Cuesta, Patricia Gassó, Amalia Lafuente, Miquel Bernardo, Sergi Mas

**Affiliations:** 1 Department of Medicine, University of Barcelona, Barcelona, Spain; 2 Department of Clinical Foundations, Pharmacology Unit, University of Barcelona, Barcelona, Spain; 3 Centro de Investigación Biomédica en Red de Salud Mental (CIBERSAM), Carlos III Health Institute, Barcelona, Spain; 4 Child and Adolescent Psychiatry Department, Hospital General Universitario Gregorio Marañón, School of Medicine, Universidad Complutense, IiSGM, Madrid, Spain; 5 Hospital Ramon y Cajal, Universidad de Alcala, IRYCIS, Madrid, Spain; 6 Department of Psychiatry, Complejo Hospitalario de Navarra, Instituto de Investigación Sanitaria de Navarra (IdiSNA), Pamplona, Spain; 7 The August Pi i Sunyer Biomedical Research Institute (IDIBAPS), Barcelona, Spain; 8 Barcelona Clínic Schizophrenia Unit, Hospital Clínic de Barcelona, Barcelona, Spain

**Correction to: Transl. Psychiatry**

10.1038/s41398-018-0330-4 Published online 13 December 2018.

One of the funding sources (FEDER-Unión Europea) was not previously acknowledged in this Article. This study was supported by the Spanish Ministry of Health, Instituto de Salud Carlos III (FIS, Fondo de Investigacion Sanitaria PI13/00812, PI16/0122) and FEDER-Unión Europea.

