# Supplementary figures and images for: Improving pharmacogenetic prediction of extrapyramidal symptoms induced by antipsychotics
Source: Transl Psychiatry. 2018 Dec 13;8:276. doi: 10.1038/s41398-018-0330-4 (PMC6293322; doi:10.1038/s41398-018-0330-4)

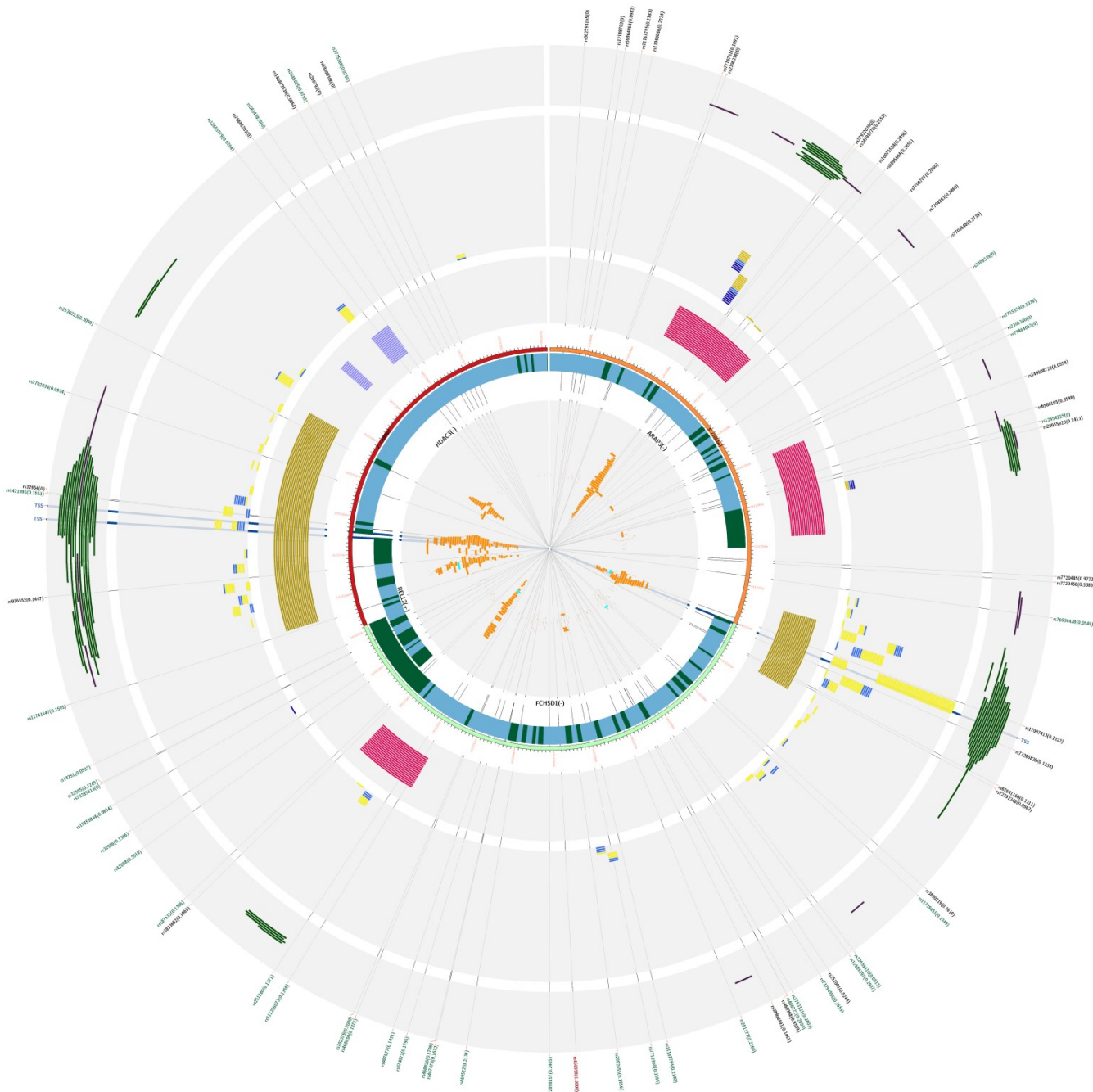

Supplement: Supplementary file 1 — Supplementary Figure1A [file 41398_2018_330_MOESM1_ESM.pdf]

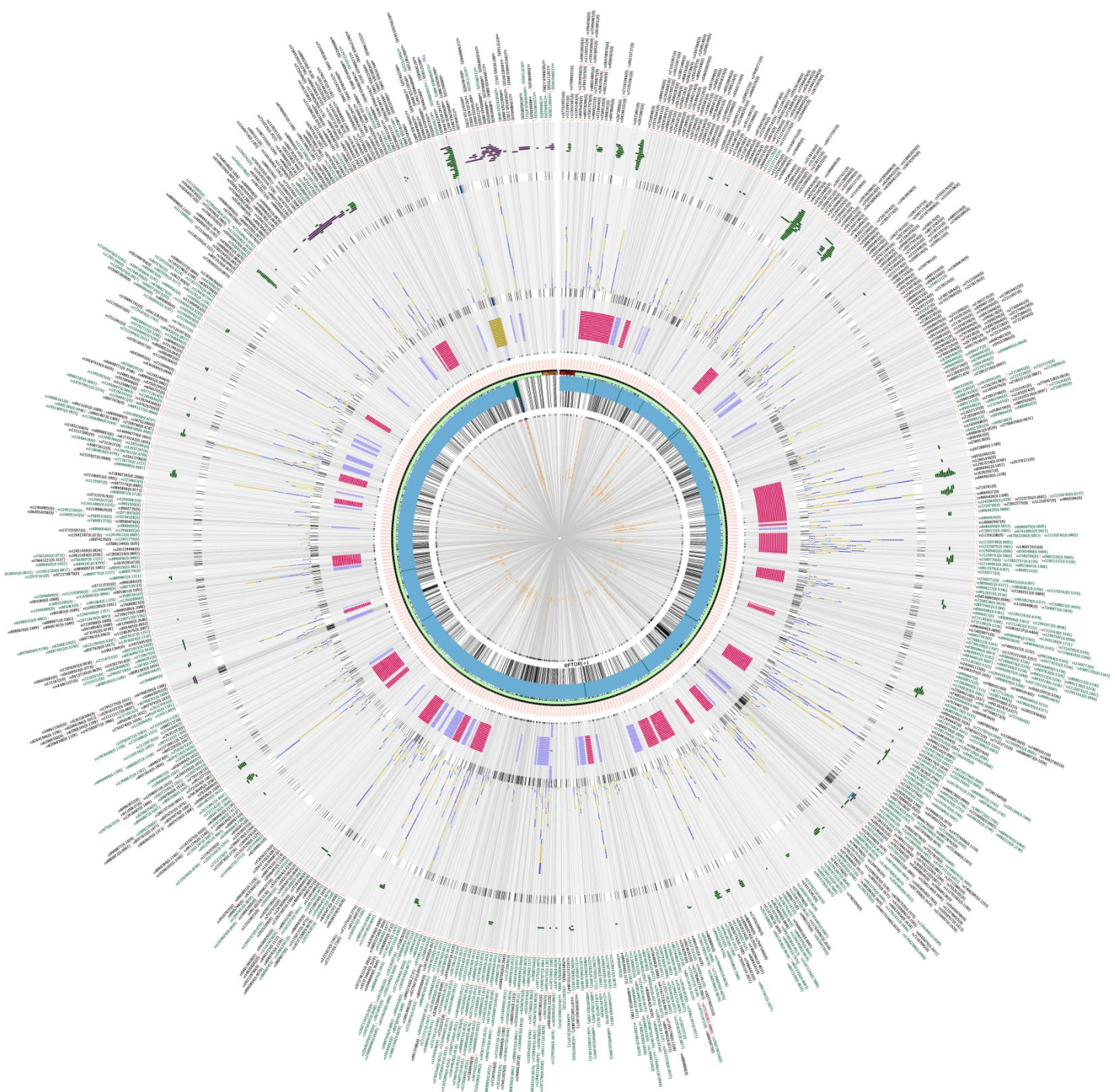

Supplement: Supplementary file 2 — Supplementary Figure1B [file 41398_2018_330_MOESM2_ESM.pdf]

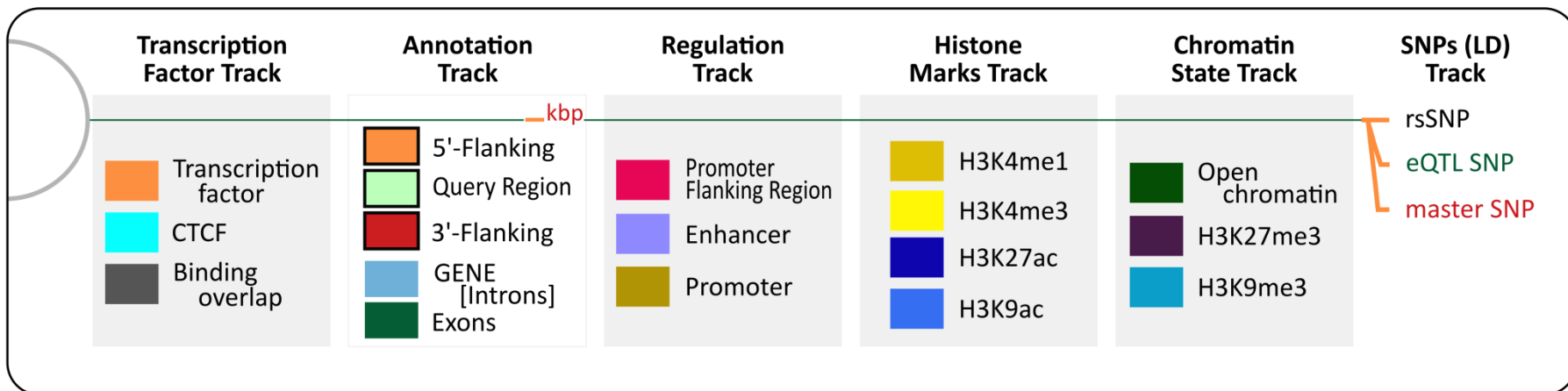

Supplement: Supplementary file 3 — Supplementary Figure1C [file 41398_2018_330_MOESM3_ESM.pdf]

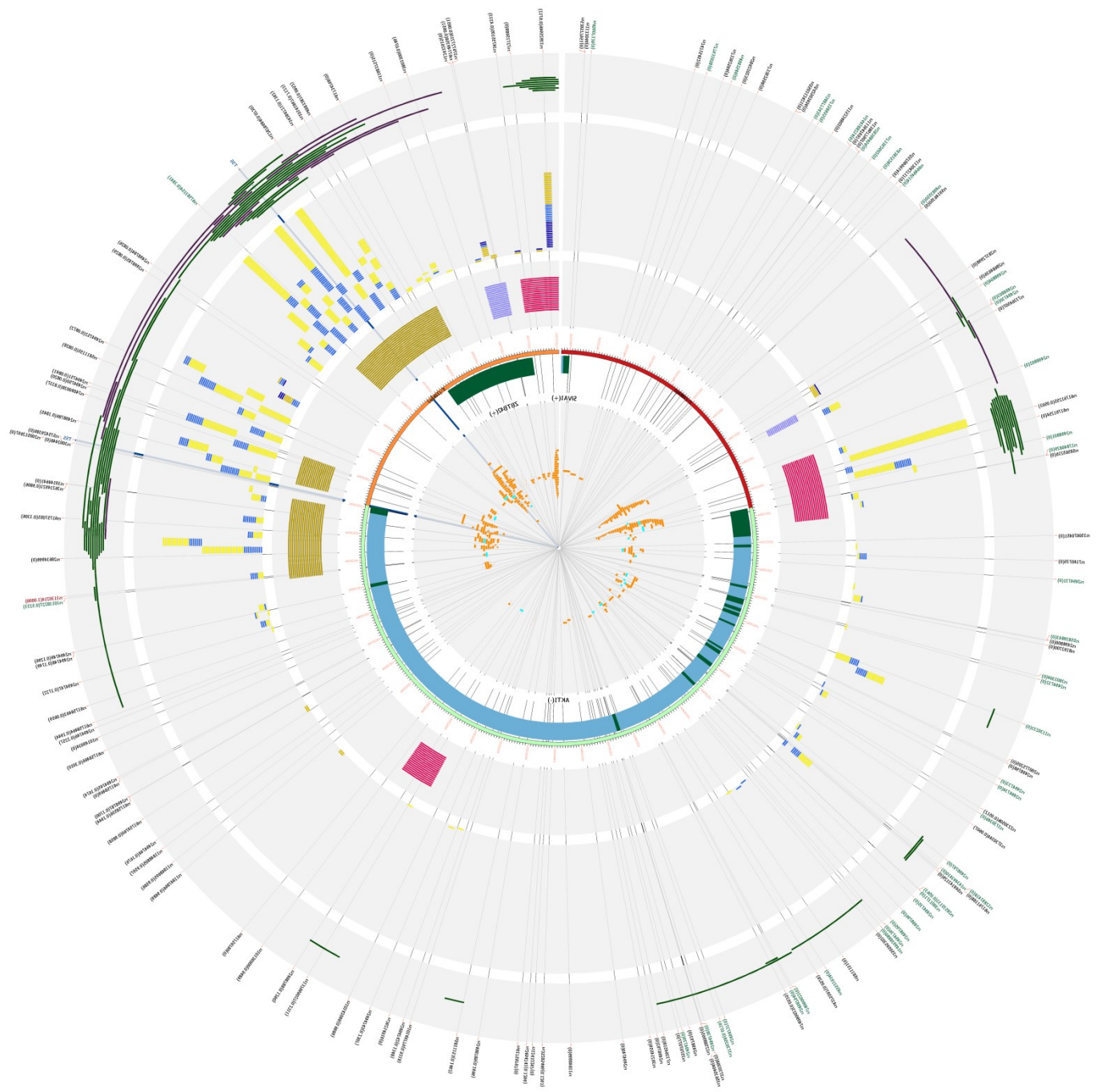

Supplement: Supplementary file 5 — Supplementary Figure 1D [file 41398_2018_330_MOESM5_ESM.pdf]

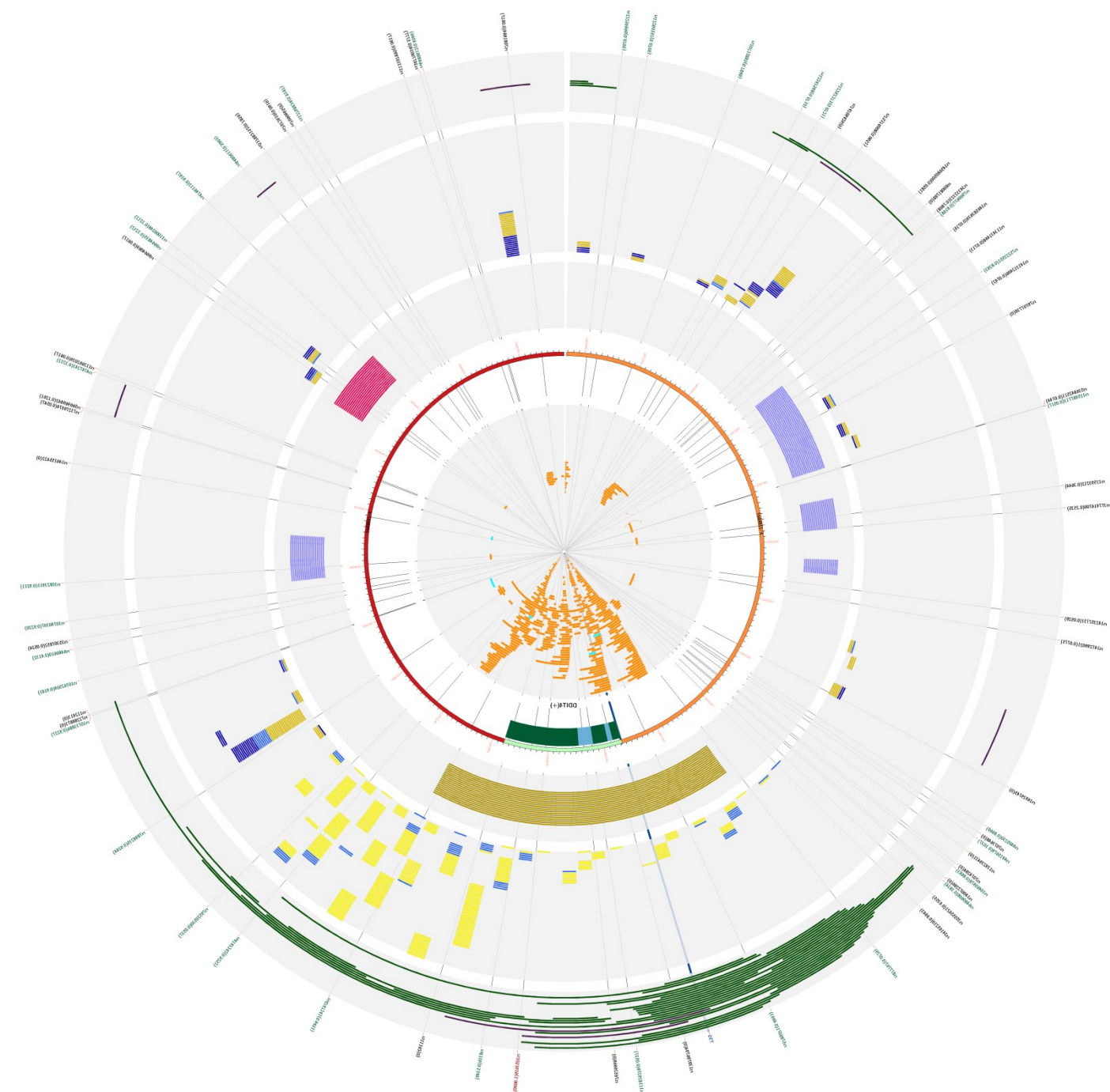

Supplement: Supplementary file 6 — Supplementary Figures [file 41398_2018_330_MOESM6_ESM.pdf]
