# Supplementary material for: Improving pharmacogenetic prediction of extrapyramidal symptoms induced by antipsychotics
Source: Transl Psychiatry. 2018 Dec 13;8:276. doi: 10.1038/s41398-018-0330-4 (PMC6293322; doi:10.1038/s41398-018-0330-4)
Supplement: Supplementary file 4 — Supplementary Table 1 and 2 [file 41398_2018_330_MOESM4_ESM.docx]

**Table S1.** Summary of the 1647 SNPs identified in the NGS.

| **Gene** | **SNP** | **Position^1^** | **SiNoPsis^2^** | **LD^3^** | **alleles** | **MAF^4^** | **HWE^4^** | **Codominant^5^** | **Dominant^5^** | **Recessive^5^** | **Overdominant^5^** | **log-additive^5^** |
| --- | --- | --- | --- | --- | --- | --- | --- | --- | --- | --- | --- | --- |
| AKT1 | rs1133044 | 105225979 | eSNP | 0 | C/G | 78,4 | 1 | 0,90144 | 0,66142 | 1 | 0,65278 | 0,71302 |
| AKT1 | rs45607139 | 105226075 | eSNP | 0 | C/T | 65,3 | 0,637214 | 0,93492 | 1 | 0,72474 | 0,83112 | 0,86891 |
| AKT1 | rs77631658 | 105227822 | eSNP | 0 | A/G | 76,1 | 1 | 0,8954 | 0,82902 | 0,64411 | 1 | 0,7234 |
| AKT1 | rs4983549 | 105228216 | eSNP | 0 | G/A | 63,1 | 0,49209 | 0,97408 | 0,82571 | 1 | 0,83112 | 0,86939 |
| AKT1 | rs139363481 | 105229268 | normSNP | 0 | A/C | 77,8 | 0,756052 | 0,88877 | 1 | 0,64411 | 0,82058 | 0,85905 |
| AKT1 | - | 105229555 | normSNP | 0 | A/C | 81,8 | 0,726269 | 0,00157 | 0,00639 | 0,49425 | 0,00112 | 0,00157 |
| AKT1 | rs7158655 | 105229646 | eSNP | 0 | T/C | 66,5 | 0,812114 | 0,88646 | 0,66683 | 0,72474 | 0,83078 | 0,62376 |
| AKT1 | - | 105230225 | normSNP | 0 | A/C | 64,2 | 0,646972 | 0,97442 | 0,82757 | 1 | 0,83112 | 0,87095 |
| AKT1 | rs73362602 | 105230595 | eSNP | 0 | C/T | 58 | 0,187848 | 0,10348 | 0,15952 | 0,05731 | 1 | 0,04284 |
| AKT1 | rs4381528 | 105230815 | eSNP | 0 | C/T | 82,4 | 0,724368 | 0,00131 | 0,00112 | 0,55323 | 0,00027 | 0,00924 |
| AKT1 | rs66464514 | 105231196 | eSNP | 0 | A/G | 63,1 | 0,251473 | 0,93584 | 1 | 0,72474 | 0,83078 | 0,86574 |
| AKT1 | rs4983550 | 105231640 | eSNP | 0 | T/G | 60,8 | 0,370164 | 0,60042 | 1 | 0,33085 | 0,5213 | 0,62121 |
| AKT1 | rs2498804 | 105233095 | ecreSNP | 0 | C/A | 65,9 | 0,641308 | 0,93315 | 0,82902 | 0,72474 | 1 | 0,74256 |
| AKT1 | rs2498802 | 105234442 | eSNP | 0 | G/C | 60,8 | 0,370164 | 0,60042 | 1 | 0,33085 | 0,5213 | 0,62121 |
| AKT1 | rs2498801 | 105235558 | ecreSNP | 0 | T/C | 61,9 | 0,503103 | 0,59786 | 1 | 0,33085 | 0,52216 | 0,62627 |
| AKT1 | rs2494731 | 105237680 | eSNP | 0 | G/C | 63,1 | 0,251473 | 0,93584 | 1 | 0,72474 | 0,83078 | 0,86574 |
| AKT1 | - | 105238591 | normSNP | 0 | A/C | 69,3 | 0,801789 | 0,88437 | 0,83112 | 0,72474 | 0,66449 | 1 |
| AKT1 | rs12590657 | 105238592 | normSNP | 0 | C/G | 80,1 | 0,000662 | 0,91842 | 1 | 0,72474 | 0,7871 | 0,8729 |
| AKT1 | rs2498800 | 105238604 | normSNP | 0 | C/T | 66,5 | 0,812114 | 0,88646 | 0,66683 | 0,72474 | 0,83078 | 0,62376 |
| AKT1 | rs3803304 | 105239146 | eSNP | 0 | C/G | 71 | 0,796007 | 0,97492 | 0,83112 | 1 | 0,82757 | 0,87018 |
| AKT1 | rs2494732 | 105239192 | eSNP | 0 | T/C | 54,5 | 1 | 0,61202 | 0,34928 | 1 | 0,39344 | 0,54285 |
| AKT1 | rs1130233 | 105239894 | ecreSNP | 0 | C/T | 72,7 | 0,185157 | 0,87582 | 0,83008 | 0,72474 | 0,65278 | 1 |
| AKT1 | rs2494733 | 105240784 | eSNP | 0 | C/G | 65,3 | 0,637214 | 0,88437 | 0,66449 | 0,72474 | 0,83112 | 0,62034 |
| AKT1 | rs2494734 | 105240885 | eSNP | 0 | G/C | 58 | 0,380514 | 0,09806 | 0,48769 | 0,0314 | 0,39146 | 0,10171 |
| AKT1 | rs3730346 | 105241378 | eSNP | 0 | C/T | 81,2 | 0,725846 | 0,00077 | 0,00333 | 0,49425 | 0,00053 | 0,00077 |
| AKT1 | rs2498797 | 105242228 | ecreSNP | 0 | T/C | 61,9 | 0,263582 | 0,73103 | 0,8234 | 0,50044 | 0,5213 | 0,8676 |
| AKT1 | rs12897418 | 105242374 | ecreSNP | 0 | G/A | 81,8 | 0,726269 | 0,00157 | 0,00639 | 0,49425 | 0,00112 | 0,00157 |
| AKT1 | rs28535133 | 105242826 | eSNP | 0,0563 | C/T | 78,4 | 1 | 0,00517 | 0,00807 | 0,29556 | 0,0014 | 0,06348 |
| AKT1 | rs3001371 | 105242831 | eSNP | 0 | C/T | 66,5 | 0,812114 | 0,88646 | 0,66683 | 0,72474 | 0,83078 | 0,62376 |
| AKT1 | rs2494735 | 105242966 | eSNP | 0 | T/C | 60,8 | 0,370164 | 0,94539 | 1 | 0,74706 | 0,83078 | 0,86923 |
| AKT1 | rs2498796 | 105243220 | eSNP | 0 | G/A | 68,8 | 1 | 0,97529 | 0,83078 | 1 | 0,83008 | 0,86875 |
| AKT1 | rs148209792 | 105243605 | normSNP | 0 | G/C | 78,4 | 0,063019 | 1 | 0,82902 | 1 | 1 | 1 |
| AKT1 | rs139394329 | 105243606 | normSNP | 0 | G/C | 77,8 | - | 0,51955 | - | - | - | - |
| AKT1 | rs149938880 | 105243623 | ecreSNP | 0 | C/G | 76,7 | 0,03386 | 0,83065 | 0,66847 | 1 | 0,83008 | 0,83065 |
| AKT1 | - | 105243640 | normSNP | 0 | G/C | 86,4 | - | 0,33749 | - | - | - | - |
| AKT1 | rs45598737 | 105244239 | normSNP | 0 | C/T | 87,5 | - | 0,00254 | - | - | - | - |
| AKT1 | rs5811151 | 105244345 | normSNP | 0 | A/C | 79,5 | 1 | 0,1368 | 0,04645 | 0,55323 | 0,07072 | 0,05308 |
| AKT1 | rs2498794 | 105245251 | ecreSNP | 0 | G/A | 54,5 | 0,672306 | 0,0839 | 0,0963 | 0,0557 | 1 | 0,02774 |
| AKT1 | rs12437017 | 105245951 | normSNP | 0 | A/C | 88,6 | - | 0,30762 | - | - | - | - |
| AKT1 | rs111268345 | 105245969 | normSNP | 0 | C/T | 68,8 | - | 0,50866 | - | - | - | - |
| AKT1 | rs2494737 | 105246325 | eSNP | 0 | T/A | 64,2 | 0,358877 | 0,42874 | 0,66142 | 0,28706 | 0,28578 | 0,86744 |
| AKT1 | rs3730358 | 105246407 | eSNP | 0,0716 | G/A | 78,4 | 0,75223 | 0,51853 | 0,2755 | 0,55323 | 0,37493 | 0,25176 |
| AKT1 | rs2498792 | 105247366 | normSNP | 0 | G/A | 96,6 | 0,084231 | 0,05544 | 0,05544 | 1 | 0,11643 | 0,05544 |
| AKT1 | rs2494740 | 105247881 | normSNP | 0 | T/A | 61,9 | 0,364895 | 0,30472 | 1 | 0,15304 | 0,27966 | 0,46048 |
| AKT1 | rs2498791 | 105248470 | normSNP | 0 | G/T | 60,8 | 0,370164 | 0,15176 | 0,17614 | 0,33085 | 0,05358 | 0,62121 |
| AKT1 | rs11848899 | 105248660 | normSNP | 0 | G/T | 79,5 | 1 | 0,29504 | 0,12215 | 0,55323 | 0,17614 | 0,12313 |
| AKT1 | rs10149779 | 105251086 | normSNP | 0,9318 | G/A | 67 | 0,051221 | 0,00068 | 0,01588 | 0,05544 | 0,00053 | 0,00068 |
| AKT1 | rs10142069 | 105252070 | normSNP | 0,9686 | T/C | 65,9 | 0,059461 | 0,00177 | 0,00807 | 0,07799 | 0,00053 | 0,14947 |
| AKT1 | - | 105252763 | normSNP | 0 | A/C | 86,9 | 0,000023 | 0,43562 | 0,26682 | 0,23023 | 0,72474 | 0,20427 |
| AKT1 | - | 105252771 | normSNP | 0 | C/G | 57,4 | 0 | 0,88766 | 0,83112 | 0,65754 | 0,74706 | 0,72914 |
| AKT1 | - | 105252772 | normSNP | 0 | T/G | 61,4 | 0 | 0,4438 | 0,66449 | 0,37493 | 0,29556 | 0,50064 |
| AKT1 | - | 105252773 | normSNP | 0 | T/C | 69,3 | 0 | 0,65493 | 1 | 0,63199 | 0,39341 | 0,80953 |
| AKT1 | rs560517072 | 105252775 | normSNP | 0 | C/G | 63,6 | 0 | 0,41486 | 0,82571 | 0,50293 | 0,49425 | 0,41486 |
| AKT1 | rs2498788 | 105253009 | normSNP | 129 | G/A | 93,8 | 0,030082 | 0,92496 | 0,72474 | 1 | 0,69315 | 0,78626 |
| AKT1 | rs10136000 | 105253581 | normSNP | 0,9489 | G/A | 67,6 | 0,052713 | 0,00031 | 0,00876 | 0,05544 | 0,00023 | 0,00031 |
| AKT1 | rs11847866 | 105255205 | normSNP | 0,9489 | G/A | 71,6 | 0,792807 | 0,01634 | 0,0872 | 0,07799 | 0,0093 | 0,48962 |
| AKT1 | rs11848695 | 105255405 | normSNP | 0,9586 | G/A | 66,5 | 0,030101 | 0,00142 | 0,02768 | 0,05544 | 0,00114 | 0,00142 |
| AKT1 | rs11848805 | 105255658 | normSNP | 0,9597 | G/A | 67 | 0,051221 | 0,00199 | 0,04897 | 0,05544 | 0,00249 | 0,00199 |
| AKT1 | rs2494744 | 105255814 | normSNP | 0,1676 | G/A | 92,6 | 0,061363 | 0,93973 | 0,74706 | 1 | 0,72474 | 0,79664 |
| AKT1 | rs61759760 | 105256187 | normSNP | 0,9858 | C/T | 66,5 | 0,030101 | 0,00142 | 0,02768 | 0,05544 | 0,00114 | 0,00142 |
| AKT1 | rs61758556 | 105256575 | normSNP | 0,1944 | A/T | 79,5 | 0,508429 | 0,2008 | 0,07879 | 1 | 0,07509 | 0,10939 |
| AKT1 | rs2498787 | 105256581 | normSNP | 0,17 | C/T | 92 | 0,082844 | 0,39529 | 0,21023 | 1 | 0,17368 | 0,30914 |
| AKT1 | rs2494745 | 105256913 | normSNP | 0,1674 | C/G | 92,6 | 0,061363 | 0,93973 | 0,74706 | 1 | 0,72474 | 0,79664 |
| AKT1 | rs61758466 | 105257354 | normSNP | 301 | G/A | 79 | 0,104249 | 0,02873 | 0,0293 | 1 | 0,01588 | 0,02873 |
| AKT1 | rs2494746 | 105257719 | normSNP | 0,2257 | G/C | 90,3 | 0,002441 | 0,55678 | 0,76375 | 0,29556 | 0,72474 | 0,51828 |
| AKT1 | rs61758464 | 105257802 | normSNP | 0,1944 | G/A | 79,5 | 0,508429 | 0,2008 | 0,07879 | 1 | 0,07509 | 0,10939 |
| AKT1 | rs61758463 | 105258067 | normSNP | 0,0659 | G/A | 69,3 | 1 | 0,90457 | 0,66944 | 1 | 0,66683 | 0,74179 |
| AKT1 | rs2494747 | 105258437 | creSNP | 0,1722 | T/G | 54,5 | 0,829506 | 0,39772 | 0,24675 | 0,79553 | 0,19965 | 0,55193 |
| AKT1 | rs2494748 | 105258892 | creSNP | 0,1749 | T/C | 58 | 0,078597 | 0,07065 | 0,23605 | 0,10141 | 0,0293 | 1 |
| AKT1 | rs2494749 | 105258893 | creSNP | 0,1296 | G/A | 94,9 | 0,193471 | 0,7133 | 0,45614 | 1 | 0,69315 | 0,7133 |
| AKT1 | rs10138227 | 105259706 | ecreSNP | 0,5233 | C/T | 87,5 | 0,615347 | 0,58741 | 0,61069 | 0,49425 | 1 | 0,58741 |
| AKT1 | rs1130214 | 105259734 | creSNP | 1 | C/A | 65,9 | 0,017676 | 0,00279 | 0,04645 | 0,05544 | 0,00235 | 0,00279 |
| AKT1 | rs61757055 | 105260906 | creSNP | 0,1306 | A/C | 50,6 | 0 | 0,43329 | 0,19965 | 0,28477 | 0,64411 | 0,22656 |
| AKT1 | rs36214921 | 105261414 | creSNP | 0,9806 | G/C | 65,9 | 0,017676 | 0,00279 | 0,04645 | 0,05544 | 0,00235 | 0,00279 |
| AKT1 | rs2498786 | 105262368 | creSNP | 0,1845 | G/C | 58,5 | 0,084069 | 0,04626 | 0,15952 | 0,10141 | 0,01685 | 0,86469 |
| AKT1 | rs74090038 | 105262781 | creSNP | 0,8337 | C/T | 68,2 | 0,025373 | 0,02821 | 0,19248 | 0,11643 | 0,0315 | 0,02821 |
| AKT1 | rs2494750 | 105262912 | creSNP | 82 | C/G | 92,6 | 0,379683 | 1 | 1 | 1 | 0,74706 | 1 |
| AKT1 | rs2494751 | 105262961 | creSNP | 0,0841 | A/G | 92 | 0,429156 | 0,75737 | 0,76375 | 1 | 0,5336 | 0,75737 |
| AKT1 | rs5811155 | 105263348 | creSNP | 0,0828 | A/C | 89,8 | 0,004197 | 0,79582 | 0,55939 | 1 | 0,50044 | 0,67161 |
| AKT1 | rs2494752 | 105263608 | creSNP | 0,0872 | G/A | 92,6 | 0,379683 | 1 | 1 | 1 | 0,74706 | 1 |
| AKT1 | rs2498785 | 105264826 | normSNP | 0,0829 | G/A | 93,2 | 0,330711 | 0,7387 | 0,74706 | 1 | 0,50044 | 0,7387 |
| AKT1 | rs2498784 | 105264963 | normSNP | 0,0826 | G/A | 92,6 | 0,379683 | 1 | 1 | 1 | 0,74706 | 1 |
| AKT1 | rs67583154 | 105266092 | ecreSNP | 0,3841 | G/A | 88,1 | 0,343361 | 0,63519 | 0,79553 | 0,49425 | 0,7871 | 0,63519 |
| AKT1 | rs12878684 | 105267579 | creSNP | 72 | G/A | 91,5 | 0,478488 | 0,54939 | 0,55939 | 1 | 0,36558 | 0,54939 |
| AKT1 | rs34284721 | 105267934 | creSNP | 0,1362 | G/A | 82,4 | 0,290417 | 0,50026 | 0,36782 | 1 | 0,49599 | 0,50026 |
| AKT1 | rs10141867 | 105268104 | normSNP | 0,7123 | G/A | 68,8 | 0,133553 | 0,0958 | 0,19585 | 0,15362 | 0,05409 | 0,58751 |
| AKT1 | rs4983387 | 105268228 | normSNP | 0,0952 | A/G | 89,8 | 1 | 1 | 0,7871 | 1 | 1 | 1 |
| AKT1 | rs3803300 | 105269779 | normSNP | 0,0746 | C/T | 88,6 | 0,010855 | 0,82265 | 0,58001 | 1 | 0,5336 | 0,67928 |
| AKT1 | rs35721922 | 105270483 | normSNP | 0 | A/C | 50 | 0,1346 | 0,86087 | 1 | 0,59681 | 0,66449 | 0,73882 |
| AKT1 | rs67749358 | 105270485 | normSNP | 0,0651 | A/C | 68,2 | 0 | 0,43193 | 0,2755 | 0,80249 | 0,24091 | 0,44693 |
| AKT1 | - | 105271144 | normSNP | 0 | A/C | 89,2 | 0,251782 | 0,24222 | 0,17437 | 0,49425 | 0,39373 | 0,24222 |
| AKT1 | rs72715988 | 105271352 | normSNP | 0 | C/T | 87,5 | 0,123522 | 0,31435 | 0,79553 | 0,24138 | 0,58001 | 0,31435 |
| AKT1 | rs33925946 | 105271836 | creSNP | 0,6712 | G/T | 69,9 | 0,073579 | 0,03293 | 0,28274 | 0,11643 | 0,05409 | 0,03293 |
| FCHSD1 | rs250793 | 141011187 | normSNP | 0 | C/T | 98,9 | - | 1 | - | - | - | - |
| FCHSD1 | rs2530223 | 141014494 | eSNP | 0,3099 | C/T | 58,5 | 0,509015 | 0,95863 | 1 | 0,7871 | 0,83008 | 0,88334 |
| FCHSD1 | rs7702834 | 141015242 | eSNP | 0,0934 | G/T | 86,4 | 0,654989 | 0,59215 | 0,32371 | 1 | 0,30762 | 0,38629 |
| FCHSD1 | rs32954 | 141016276 | creSNP | 0 | C | 100 | - | - | - | - | - | - |
| FCHSD1 | rs1421896 | 141016288 | ecreSNP | 0,3553 | G/T | 59,7 | 0,26813 | 0,95797 | 1 | 0,7871 | 0,82902 | 0,88489 |
| FCHSD1 | rs976552 | 141017339 | creSNP | 0,1447 | A/C | 79,5 | 0,750596 | 0,17687 | 0,07509 | 1 | 0,06568 | 0,13666 |
| FCHSD1 | rs11741647 | 141018454 | eSNP | 0,1595 | C/G | 84,7 | 0,026078 | 0,32626 | 0,1377 | 0,64411 | 0,17437 | 0,1879 |
| FCHSD1 | rs14251 | 141019110 | eSNP | 0,0592 | C/A | 56,8 | 0,516631 | 0,40022 | 0,17614 | 0,59681 | 0,39146 | 0,23885 |
| FCHSD1 | rs32955 | 141019313 | eSNP | 0,1249 | C/T | 79,5 | 0,508429 | 0,67103 | 0,38076 | 1 | 0,37493 | 0,42553 |
| FCHSD1 | rs73285814 | 141019324 | eSNP | 0 | G/A | 79,5 | 0,1819 | 0,57048 | 0,36782 | 0,39341 | 0,63199 | 0,29655 |
| FCHSD1 | rs17855844 | 141019569 | eSNP | 0,0654 | G/C | 81,2 | 0,001631 | 0,38872 | 0,23605 | 1 | 0,17437 | 0,4075 |
| FCHSD1 | rs11742646 | 141019830 | normSNP | 0 | C/G | 83 | 0,015563 | 0,56202 | 0,33749 | 0,39341 | 0,59681 | 0,28486 |
| FCHSD1 | rs32956 | 141019881 | eSNP | 0,1366 | G/C | 79,5 | 1 | 0,5143 | 0,27021 | 0,55323 | 0,36782 | 0,24888 |
| FCHSD1 | rs41098 | 141020100 | eSNP | 0,2018 | C/A | 72,2 | 0,432002 | 0,76978 | 0,66976 | 0,64411 | 0,51955 | 0,85905 |
| FCHSD1 | rs187515 | 141020765 | eSNP | 0,1366 | T/C | 79 | 0,752823 | 0,62903 | 0,38076 | 0,55323 | 0,50293 | 0,33869 |
| FCHSD1 | rs3833652 | 141020848 | normSNP | 0,1965 | A/C | 77,3 | 0,764065 | 0,63566 | 0,51329 | 0,64411 | 0,36782 | 0,72283 |
| FCHSD1 | rs149876519 | 141020849 | normSNP | 0 | A/C | 80,1 | 0,316322 | 0,72935 | 0,65278 | 0,64411 | 0,47784 | 0,85763 |
| FCHSD1 | rs251180 | 141021935 | eSNP | 0,1371 | A/G | 79 | 0,752823 | 0,62903 | 0,38076 | 0,55323 | 0,50293 | 0,33869 |
| FCHSD1 | - | 141022112 | normSNP | 0 | A/C | 89,8 | - | 1 | - | - | - | - |
| FCHSD1 | - | 141022113 | normSNP | 0 | A/C | 87,5 | 0,615347 | 1 | 1 | 1 | 1 | 1 |
| FCHSD1 | rs702380 | 141022544 | normSNP | 0 | A/G | 75 | 0,253217 | 0,72734 | 0,5213 | 0,55323 | 0,66683 | 0,44913 |
| FCHSD1 | rs702379 | 141022848 | eSNP | 0,204 | T/C | 76,7 | 0,381822 | 0,41101 | 0,19585 | 0,55323 | 0,2755 | 0,18327 |
| FCHSD1 | rs468650 | 141022882 | eSNP | 0,1371 | C/T | 79 | 0,752823 | 0,62903 | 0,38076 | 0,55323 | 0,50293 | 0,33869 |
| FCHSD1 | rs467677 | 141023281 | eSNP | 0,1431 | A/G | 79,5 | 0,1819 | 0,62317 | 0,36782 | 1 | 0,33749 | 0,48735 |
| FCHSD1 | rs468920 | 141023723 | eSNP | 0,1706 | T/C | 73,9 | 0,782866 | 0,84628 | 0,83078 | 0,64411 | 0,66449 | 1 |
| FCHSD1 | rs467478 | 141023733 | eSNP | 0,1672 | G/A | 74,4 | 0,411639 | 0,97614 | 0,83078 | 1 | 0,82902 | 0,85509 |
| FCHSD1 | rs469522 | 141024048 | eSNP | 0,2139 | A/G | 59,7 | 0,378886 | 0,15142 | 0,11107 | 0,5336 | 0,05358 | 0,41489 |
| FCHSD1 | rs469074 | 141024136 | normSNP | 0 | T/G | 72,7 | 0,590502 | 0,84836 | 0,83112 | 0,64411 | 0,66683 | 1 |
| FCHSD1 | rs112898157 | 141024890 | eSNP | 0,2465 | A/C | 64,2 | 0,358877 | 0,27467 | 0,38076 | 0,28706 | 0,13466 | 0,86744 |
| FCHSD1 | rs456998 | 141025162 | normSNP | 1 | G/T | 50,6 | 0,526803 | 0,11514 | 0,63199 | 0,08749 | 0,05358 | 0,46533 |
| FCHSD1 | rs2052455 | 141025581 | eSNP | 0,1956 | A/G | 86,9 | 0,633425 | 0,05926 | 0,02262 | 1 | 0,0178 | 0,04546 |
| FCHSD1 | rs7711960 | 141025929 | eSNP | 0,3595 | A/G | 86,4 | 1 | 0,33197 | 0,22367 | 1 | 0,32371 | 0,33197 |
| FCHSD1 | rs11167754 | 141026094 | eSNP | 0,214 | G/A | 88,6 | 1 | 0,11868 | 0,06712 | 1 | 0,11004 | 0,11868 |
| FCHSD1 | rs251177 | 141026884 | eSNP | 0,226 | T/C | 73,3 | 0,594031 | 0,7665 | 0,66944 | 0,64411 | 0,5169 | 0,85945 |
| FCHSD1 | rs58908491 | 141027226 | normSNP | 0,1461 | G/A | 88,6 | 1 | 0,01577 | 0,0178 | 1 | 0,00683 | 0,01577 |
| FCHSD1 | rs468968 | 141027399 | normSNP | 0,9559 | C/T | 50,6 | 0,526803 | 0,33777 | 0,1493 | 0,80828 | 0,28477 | 0,30627 |
| FCHSD1 | rs468222 | 141027400 | eSNP | 0,289 | G/C | 64,8 | 0,484636 | 0,35036 | 0,51329 | 0,28706 | 0,20012 | 1 |
| FCHSD1 | rs3763121 | 141027462 | eSNP | 0,2403 | G/A | 84,7 | 1 | 0,22947 | 0,0963 | 1 | 0,08749 | 0,13881 |
| FCHSD1 | rs73794956 | 141027791 | eSNP | 0,1659 | T/A | 84,7 | 0,026078 | 0,61382 | 0,32371 | 0,64411 | 0,41692 | 0,34884 |
| FCHSD1 | rs251041 | 141027888 | normSNP | 0,5244 | G/C | 66,5 | 0,339808 | 0,97386 | 0,83078 | 1 | 0,82757 | 0,8794 |
| FCHSD1 | rs173683 | 141028047 | normSNP | 0 | G/C | 51,1 | 0,673204 | 0,30481 | 0,1493 | 1 | 0,19965 | 0,37543 |
| FCHSD1 | rs12659397 | 141028149 | eSNP | 0,2937 | T/C | 84,7 | 1 | 0,22947 | 0,0963 | 1 | 0,08749 | 0,13881 |
| FCHSD1 | rs11739451 | 141029072 | eSNP | 0,1349 | G/A | 83,5 | 0,236986 | 0,53874 | 0,47784 | 0,29556 | 0,80249 | 0,33808 |
| FCHSD1 | rs72792348 | 141030415 | creSNP | 0,0562 | G/C | 88,1 | 0,016486 | 0,31256 | 0,17437 | 1 | 0,12878 | 0,30412 |
| FCHSD1 | rs7720458 | 141032603 | normSNP | 0,5386 | G/A | 59,1 | 0,007578 | 0,74434 | 0,82902 | 0,45249 | 0,65278 | 0,58977 |
| FCHSD1 | rs7720485 | 141032661 | normSNP | 0,9722 | A/G | 52,8 | 0,057316 | 0,07834 | 0,82058 | 0,05373 | 0,04897 | 0,33427 |
| FCHSD1 | rs6580194 | 141034123 | normSNP | 0 | A/T | 65,3 | 0,487128 | 0,97401 | 0,83008 | 1 | 0,82902 | 0,87839 |
| FCHSD1 | rs28655920 | 141034183 | normSNP | 0,1413 | A/G | 88,6 | 1 | 0,01577 | 0,0178 | 1 | 0,00683 | 0,01577 |
| FCHSD1 | rs6580195 | 141034392 | normSNP | 0,3548 | C/T | 69,3 | 0,208264 | 0,56297 | 0,28578 | 0,74706 | 0,37493 | 0,35855 |
| FCHSD1 | rs371182755 | 141034792 | normSNP | 0 | A/C | 56,8 | 0,12954 | 0,68019 | 0,65754 | 0,61069 | 0,38541 | 1 |
| FCHSD1 | rs7715559 | 141035374 | eSNP | 0,1038 | G/A | 86,4 | 1 | 0,62812 | 0,46622 | 1 | 0,62227 | 0,62812 |
| FCHSD1 | rs2306339 | 141035955 | eSNP | 0 | G/A | 80,7 | 0,297649 | 0,28386 | 0,25593 | 0,15362 | 0,63199 | 0,14698 |
| FCHSD1 | rs7703648 | 141036337 | normSNP | 0,2739 | A/G | 56,8 | 1 | 0,38471 | 0,16844 | 0,58001 | 0,39344 | 0,21765 |
| FCHSD1 | rs7704263 | 141036676 | normSNP | 0,286 | A/G | 56,8 | 1 | 0,38471 | 0,16844 | 0,58001 | 0,39344 | 0,21765 |
| FCHSD1 | rs7708707 | 141036951 | normSNP | 0,286 | T/C | 56,8 | 1 | 0,38471 | 0,16844 | 0,58001 | 0,39344 | 0,21765 |
| FCHSD1 | rs6895094 | 141037277 | normSNP | 0,2855 | T/G | 55,7 | 1 | 0,50807 | 0,24675 | 0,7871 | 0,39344 | 0,35864 |
| FCHSD1 | rs10075524 | 141037377 | normSNP | 0,2856 | T/C | 56,2 | 0,829508 | 0,50105 | 0,24675 | 0,58001 | 0,52216 | 0,27679 |
| FCHSD1 | rs34798770 | 141037646 | creSNP | 0,2553 | G/A | 56,2 | 0,829508 | 0,50105 | 0,24675 | 0,58001 | 0,52216 | 0,27679 |
| FCHSD1 | rs77922030 | 141037683 | creSNP | 0 | G/A | 85,2 | 0,682452 | 0,09272 | 0,0963 | 1 | 0,05373 | 0,09272 |
| FCHSD1 | rs2306338 | 141039160 | normSNP | 0 | G/T | 85,2 | 0,682452 | 0,09272 | 0,0963 | 1 | 0,05373 | 0,09272 |
| FCHSD1 | rs11167755 | 141040048 | normSNP | 0,2183 | G/A | 55,7 | 0,004662 | 0,86737 | 0,66142 | 0,63199 | 1 | 0,59565 |
| FCHSD1 | rs12188703 | 141040299 | normSNP | 0 | C/T | 71,6 | 0,60713 | 0,13786 | 0,08686 | 0,12995 | 0,38076 | 0,04825 |
| DDIT4 | rs10733885 | 74024873 | eSNP | 0,7846 | G/T | 62,5 | 0,364913 | 0,00403 | 0,37493 | 0,00423 | 0,00995 | 0,50751 |
| DDIT4 | rs7900677 | 74026591 | eSNP | 0,8168 | G/A | 63,1 | 0,49209 | 0,00257 | 0,27021 | 0,00423 | 0,00522 | 0,62164 |
| DDIT4 | - | 74027086 | normSNP | 0 | A/C | 72,7 | 0,000862 | 0,0186 | 0,12636 | 0,00483 | 0,62227 | 0,01907 |
| DDIT4 | rs11593212 | 74028706 | normSNP | 0,3644 | T/C | 58 | 1 | 0,02262 | 0,25593 | 0,04367 | 0,01003 | 0,75694 |
| DDIT4 | rs71479434 | 74028744 | normSNP | 0 | T/C | 76,7 | 0,138744 | 0,42718 | 0,83008 | 0,49425 | 0,5169 | 0,42718 |
| DDIT4 | rs200224739 | 74028752 | normSNP | 0 | C/T | 86,4 | - | 0,63199 | - | - | - | - |
| DDIT4 | - | 74029169 | normSNP | 0 | A/C | 64,2 | 0,245052 | 0,01657 | 0,05255 | 0,24091 | 0,00426 | 0,46003 |
| DDIT4 | rs4405230 | 74031208 | eSNP | 0,8668 | G/T | 63,1 | 0,49209 | 0,00257 | 0,27021 | 0,00423 | 0,00522 | 0,62164 |
| DDIT4 | rs147667360 | 74031312 | normSNP | 0 | A/C | 86,9 | - | 0,22367 | - | - | - | - |
| DDIT4 | rs4415679 | 74031313 | eSNP | 0,1037 | G/A | 58 | 0,829388 | 0,01659 | 0,36782 | 0,02425 | 0,00995 | 0,54517 |
| DDIT4 | rs6480608 | 74031694 | eSNP | 0,3834 | C/T | 79,5 | 0,000001 | 0,41549 | 0,63199 | 0,21023 | 0,5336 | 0,37129 |
| DDIT4 | rs35555621 | 74031815 | creSNP | 0,6355 | A/C | 80,1 | 0 | 0,10344 | 0,11004 | 0,0557 | 1 | 0,10344 |
| DDIT4 | - | 74031941 | normSNP | 0 | A/C | 67 | 0,628925 | 0,00869 | 0,19585 | 0,01888 | 0,00995 | 1 |
| DDIT4 | - | 74033220 | normSNP | 0 | A/C | 69,9 | 0,000079 | 0,16815 | 0,5169 | 0,26682 | 0,07775 | 0,89082 |
| DDIT4 | rs1053639 | 74035041 | ecreSNP | 1 | T/A | 63,1 | 0,49209 | 0,00257 | 0,27021 | 0,00423 | 0,00522 | 0,62164 |
| DDIT4 | rs8316 | 74035297 | ecreSNP | 0,5349 | C/T | 60,2 | 0,82439 | 0,01741 | 0,26372 | 0,0314 | 0,01003 | 0,75273 |
| DDIT4 | rs4747241 | 74036429 | ecreSNP | 0,6451 | C/T | 62,5 | 0,364913 | 0,00403 | 0,37493 | 0,00423 | 0,00995 | 0,50751 |
| DDIT4 | rs4747242 | 74037193 | ecreSNP | 0,9252 | A/C | 62,5 | 0,364913 | 0,00403 | 0,37493 | 0,00423 | 0,00995 | 0,50751 |
| DDIT4 | rs7898235 | 74038181 | ecreSNP | 0,9244 | C/A | 63,1 | 0,49209 | 0,00257 | 0,27021 | 0,00423 | 0,00522 | 0,62164 |
| DDIT4 | rs10733886 | 74039074 | eSNP | 0,9227 | A/C | 63,1 | 0,49209 | 0,00257 | 0,27021 | 0,00423 | 0,00522 | 0,62164 |
| DDIT4 | rs142400744 | 74039085 | normSNP | 0 | A/C | 61,9 | 0,116586 | 0,00994 | 0,36782 | 0,00902 | 0,0176 | 0,60663 |
| DDIT4 | rs10762504 | 74039335 | eSNP | 0,9181 | C/A | 63,1 | 0,49209 | 0,00257 | 0,27021 | 0,00423 | 0,00522 | 0,62164 |
| DDIT4 | rs6480610 | 74039563 | eSNP | 0,4132 | A/G | 65,9 | 0,233625 | 0,05231 | 0,83078 | 0,0314 | 0,07879 | 0,37081 |
| DDIT4 | rs10740397 | 74039762 | eSNP | 922 | G/A | 67 | 1 | 0,02834 | 0,83008 | 0,00902 | 0,19823 | 0,19139 |
| DDIT4 | rs10823911 | 74040034 | eSNP | 0,9211 | A/C | 61,4 | 0,821753 | 0,00808 | 0,37493 | 0,00983 | 0,01003 | 0,5239 |
| DDIT4 | rs4747243 | 74041547 | eSNP | 0,3212 | A/T | 56,2 | 1 | 0,04099 | 0,35932 | 0,0557 | 0,01836 | 0,64955 |
| DDIT4 | rs9664835 | 74042533 | eSNP | 0,3212 | A/G | 58,5 | 1 | 0,00577 | 0,36782 | 0,00864 | 0,00522 | 0,44227 |
| DDIT4 | - | 74042538 | normSNP | 0 | A/C | 60,8 | 0,000036 | 0,67542 | 1 | 0,46622 | 0,46622 | 0,70059 |
| DDIT4 | rs11000248 | 74042601 | eSNP | 0,3212 | G/A | 56,8 | 1 | 0,00669 | 0,16844 | 0,02425 | 0,0026 | 0,75859 |
| DDIT4 | rs4746112 | 74042995 | eSNP | 0,9197 | G/A | 63,1 | 0,49209 | 0,00257 | 0,27021 | 0,00423 | 0,00522 | 0,62164 |
| DDIT4 | rs6480611 | 74043334 | eSNP | 0,5003 | T/C | 61,4 | 0,660087 | 0,00538 | 1 | 0,00232 | 0,0315 | 0,12754 |
| DDIT4 | rs7090665 | 74043803 | normSNP | 0 | C/T | 93,8 | - | 0,74706 | - | - | - | - |
| DDIT4 | rs12768834 | 74043874 | eSNP | 0,9197 | A/G | 62,5 | 0,364913 | 0,00403 | 0,37493 | 0,00423 | 0,00995 | 0,50751 |
| DDIT4 | rs6480612 | 74044792 | eSNP | 0,6566 | G/A | 88,6 | 0 | 0,31606 | 0,21023 | 0,12995 | 1 | 0,14776 |
| DDIT4 | rs10509767 | 74045917 | normSNP | - | A/C | 67,6 | 0,463839 | 0,26987 | 0,39295 | 0,33085 | 0,12636 | 0,87748 |
| RPTOR | - | 78509563 | normSNP | 0 | A/C | 75 | 1 | 0,41131 | 0,28274 | 0,64411 | 0,1881 | 0,47895 |
| RPTOR | rs8074979 | 78510195 | normSNP | 0 | C/T | 76,7 | 1 | 0,02798 | 0,08182 | 0,15362 | 0,01332 | 0,37499 |
| RPTOR | rs113948441 | 78510221 | normSNP | 0 | G/A | 77,3 | 1 | 0,40141 | 0,19248 | 1 | 0,18267 | 0,27035 |
| RPTOR | rs4890036 | 78512545 | normSNP | 0 | C/T | 78,4 | 1 | 0,18741 | 0,07879 | 1 | 0,07072 | 0,13899 |
| RPTOR | rs7222801 | 78513101 | eSNP | 0 | A/C | 76,1 | 0,560648 | 0,01065 | 0,08182 | 0,07799 | 0,00639 | 0,49188 |
| RPTOR | rs4890037 | 78513216 | normSNP | 0 | C/T | 78,4 | 1 | 0,18741 | 0,07879 | 1 | 0,07072 | 0,13899 |
| RPTOR | rs4889855 | 78513280 | normSNP | 0 | A/G | 81,8 | 0,469917 | 0,47706 | 0,35932 | 0,29556 | 0,63199 | 0,25779 |
| RPTOR | rs4889856 | 78513632 | normSNP | 0,0899 | T/C | 54,5 | 1 | 0,53246 | 1 | 0,28883 | 0,39344 | 0,54285 |
| RPTOR | rs113987635 | 78514207 | normSNP | 0 | A/T | 52,8 | 0,833298 | 0,48965 | 0,81312 | 0,30762 | 0,28578 | 0,65358 |
| RPTOR | rs3923310 | 78514453 | normSNP | 0 | A/G | 68,2 | 0,327159 | 0,2945 | 0,13466 | 0,33085 | 0,38076 | 0,12253 |
| RPTOR | rs9914626 | 78514938 | normSNP | 0 | C/A | 81,2 | 0 | 1 | 0,7871 | 1 | 1 | 1 |
| RPTOR | rs3923514 | 78515210 | normSNP | 0,0582 | A/G | 75 | 0,39748 | 0,12663 | 0,05101 | 0,23023 | 0,17614 | 0,04324 |
| RPTOR | rs9916371 | 78515527 | normSNP | 0,2195 | A/G | 50 | 0,1346 | 0,21999 | 0,28883 | 0,28883 | 0,08182 | 1 |
| RPTOR | rs7224941 | 78516908 | normSNP | 0 | G/A | 93,2 | 0,330711 | 0,7387 | 0,74706 | 1 | 0,50044 | 0,7387 |
| RPTOR | - | 78517287 | normSNP | 0 | A/C | 92,6 | 0,379683 | 1 | 1 | 1 | 0,74706 | 1 |
| RPTOR | rs4890039 | 78517883 | normSNP | 0,0651 | C/T | 75,6 | 0,382987 | 0,08333 | 0,0293 | 0,23023 | 0,11107 | 0,02816 |
| RPTOR | rs9674924 | 78518138 | normSNP | 0,0881 | T/C | 76,7 | 0,548234 | 0,03147 | 0,01588 | 0,07799 | 0,11107 | 0,00865 |
| RPTOR | rs11658698 | 78518327 | ecreSNP | 0,4083 | C/T | 68,2 | 0,46289 | 0,4199 | 0,83008 | 0,23023 | 0,39295 | 0,7329 |
| RPTOR | rs4890040 | 78518408 | creSNP | 0,0927 | G/A | 75,6 | 0,382987 | 0,08333 | 0,0293 | 0,23023 | 0,11107 | 0,02816 |
| RPTOR | rs11547301 | 78518780 | creSNP | 0,0645 | C/T | 76,7 | 0,548234 | 0,03147 | 0,01588 | 0,07799 | 0,11107 | 0,00865 |
| RPTOR | rs34726568 | 78519101 | creSNP | 0 | A/C | 84,1 | 1 | 0,00252 | 0,00084 | 0,49425 | 0,00347 | 0,00252 |
| RPTOR | rs12602885 | 78519169 | ecreSNP | 0 | G/A | 80,7 | 0,509961 | 0,01762 | 0,00728 | 0,49425 | 0,02351 | 0,01762 |
| RPTOR | rs200058905 | 78520329 | normSNP | 0 | A/C | 60,8 | 0 | 0,64428 | 0,5169 | 0,37493 | 0,64411 | 0,43031 |
| RPTOR | rs9908940 | 78520783 | normSNP | 0,2447 | C/T | 70,5 | 0 | 0,25836 | 0,1881 | 0,11004 | 1 | 0,111 |
| RPTOR | rs78457522 | 78520896 | eSNP | 0 | G/T | 89,8 | 1 | 0,59017 | 0,41692 | 1 | 0,58001 | 0,59017 |
| RPTOR | rs4890041 | 78521114 | normSNP | 0,0828 | A/T | 76,1 | 0,244885 | 0,05115 | 0,01588 | 0,23023 | 0,06568 | 0,01773 |
| RPTOR | rs4622563 | 78521326 | normSNP | 0 | A | 100 | - | - | - | - | - | - |
| RPTOR | rs9916171 | 78521387 | normSNP | 0,099 | T/G | 70,5 | 0,607239 | 0,0201 | 0,00522 | 0,28706 | 0,02768 | 0,00953 |
| RPTOR | rs570299617 | 78521878 | normSNP | 0 | A/C | 70,5 | 0 | 0,46768 | 0,25593 | 0,22367 | 1 | 0,22151 |
| RPTOR | rs11654614 | 78522296 | normSNP | 0,0987 | G/C | 65,9 | 0,008275 | 0,0361 | 0,01003 | 0,26682 | 0,06568 | 0,02382 |
| RPTOR | rs9894515 | 78523600 | normSNP | 0 | A/T | 93,2 | 0,330711 | 0,7387 | 0,74706 | 1 | 0,50044 | 0,7387 |
| RPTOR | rs5003528 | 78524130 | normSNP | 0 | T/C | 94,3 | 0,32747 | 0,50044 | - | - | - | - |
| RPTOR | rs4627412 | 78524244 | normSNP | 0,1046 | A/G | 71 | 1 | 0,03248 | 0,01003 | 0,23023 | 0,05101 | 0,01054 |
| RPTOR | rs9911978 | 78524407 | normSNP | 0,1046 | A/G | 70,5 | 0,80348 | 0,03625 | 0,01003 | 0,45614 | 0,0293 | 0,02026 |
| RPTOR | rs10438820 | 78524597 | normSNP | 0,1046 | T/C | 70,5 | 0,80348 | 0,03625 | 0,01003 | 0,45614 | 0,0293 | 0,02026 |
| RPTOR | rs9902500 | 78524727 | normSNP | 0 | G/T | 94,3 | 0,32747 | 0,50044 | - | - | - | - |
| RPTOR | rs9912382 | 78524756 | eSNP | 0,328 | C/T | 58,5 | 0,030329 | 0,67623 | 0,47784 | 0,50044 | 0,82757 | 0,37914 |
| RPTOR | rs9903230 | 78525050 | normSNP | 0,1046 | C/T | 68,2 | 1 | 0,14957 | 0,05358 | 0,72474 | 0,08417 | 0,10441 |
| RPTOR | rs79517096 | 78525117 | normSNP | 0,0655 | G/T | 78,4 | 0,53643 | 0,09525 | 0,04645 | 0,15362 | 0,16844 | 0,03044 |
| RPTOR | rs74003969 | 78526212 | normSNP | 0,0655 | C/T | 76,1 | 0,560648 | 0,0919 | 0,0293 | 0,39341 | 0,07072 | 0,03721 |
| RPTOR | rs12451149 | 78526245 | eSNP | 0 | T/G | 79 | 0,752823 | 0,01137 | 0,00807 | 0,24138 | 0,04345 | 0,01137 |
| RPTOR | rs4889857 | 78526498 | normSNP | 0 | A/G | 94,3 | 0,32747 | 0,50044 | - | - | - | - |
| RPTOR | rs4890042 | 78526527 | normSNP | 0 | A/G | 94,3 | 0,32747 | 0,50044 | - | - | - | - |
| RPTOR | rs12937147 | 78526565 | eSNP | 0 | T/C | 79 | 0,752823 | 0,01137 | 0,00807 | 0,24138 | 0,04345 | 0,01137 |
| RPTOR | rs4890043 | 78526831 | normSNP | 0,0883 | C/T | 77,3 | 0,764065 | 0,04104 | 0,01588 | 0,15362 | 0,07072 | 0,01155 |
| RPTOR | rs12950635 | 78526895 | eSNP | 0 | C/T | 79,5 | 0,508429 | 0,01928 | 0,00807 | 0,49425 | 0,02574 | 0,01928 |
| RPTOR | rs9898771 | 78527220 | normSNP | 0,1048 | G/A | 70,5 | 0,80348 | 0,03625 | 0,01003 | 0,45614 | 0,0293 | 0,02026 |
| RPTOR | rs7209040 | 78527294 | normSNP | 0,2458 | G/A | 50,6 | 0,199848 | 0,14776 | 0,19307 | 0,28883 | 0,05101 | 0,86939 |
| RPTOR | rs7210454 | 78528230 | normSNP | 0,1048 | G/C | 70,5 | 0,80348 | 0,03625 | 0,01003 | 0,45614 | 0,0293 | 0,02026 |
| RPTOR | rs9901646 | 78528265 | normSNP | 0,2458 | G/C | 50,6 | 0,199848 | 0,14776 | 0,19307 | 0,28883 | 0,05101 | 0,86939 |
| RPTOR | rs7212429 | 78528340 | normSNP | 0,1048 | G/A | 68,8 | 0,22335 | 0,08761 | 0,03225 | 0,74706 | 0,04645 | 0,09002 |
| RPTOR | rs6565696 | 78528492 | normSNP | 0 | C/T | 93,2 | 0,330711 | 0,7387 | 0,74706 | 1 | 0,50044 | 0,7387 |
| RPTOR | rs148803677 | 78528542 | normSNP | 0 | A/C | 65,3 | 0 | 0,82932 | 1 | 0,82058 | 0,55323 | 0,9091 |
| RPTOR | rs372123427 | 78528543 | normSNP | 0 | A/C | 64,8 | 0 | 1 | 1 | 1 | 1 | 1 |
| RPTOR | - | 78528544 | normSNP | 0 | A/C | 89,8 | 0,280048 | 0,07105 | - | - | - | - |
| RPTOR | - | 78528553 | normSNP | 0 | A/C | 87,5 | 0 | 0,30127 | 0,12878 | 0,28706 | 0,29556 | 0,16818 |
| RPTOR | rs144728806 | 78528569 | normSNP | 0,2432 | A/C | 70,5 | 0,009196 | 0,54719 | 0,28274 | 0,76375 | 0,34928 | 0,38206 |
| RPTOR | rs35128350 | 78528571 | normSNP | 0 | A/C | 89,2 | 0,000495 | 0,83245 | 0,55939 | 0,64411 | 0,72474 | 0,54662 |
| RPTOR | - | 78528585 | normSNP | 0 | A/C | 78,4 | 0,003421 | 0,13706 | 0,11107 | 0,07105 | 0,61069 | 0,05364 |
| RPTOR | rs7217900 | 78528751 | normSNP | 0,1048 | C/T | 70,5 | 0,80348 | 0,03625 | 0,01003 | 0,45614 | 0,0293 | 0,02026 |
| RPTOR | rs7218584 | 78529194 | normSNP | 0,1049 | C/T | 70,5 | 0,80348 | 0,03625 | 0,01003 | 0,45614 | 0,0293 | 0,02026 |
| RPTOR | rs12952391 | 78529826 | normSNP | 0,1182 | T/G | 57,4 | 0 | 0,01102 | 0,01003 | 0,00333 | 0,76375 | 0,00312 |
| RPTOR | rs12950400 | 78529928 | normSNP | 0,1184 | G/T | 70,5 | 0,80348 | 0,03625 | 0,01003 | 0,45614 | 0,0293 | 0,02026 |
| RPTOR | rs11651818 | 78530300 | normSNP | 0,0819 | T/C | 76,7 | 1 | 0,06532 | 0,0293 | 0,15362 | 0,11706 | 0,01953 |
| RPTOR | rs12936394 | 78530324 | normSNP | 0,1182 | C/G | 71 | 1 | 0,03248 | 0,01003 | 0,23023 | 0,05101 | 0,01054 |
| RPTOR | rs72855201 | 78530597 | normSNP | 0,0799 | A/G | 76,1 | 0,560648 | 0,0919 | 0,0293 | 0,39341 | 0,07072 | 0,03721 |
| RPTOR | rs9890210 | 78530664 | normSNP | 0,353 | C/T | 59,1 | 0,12669 | 0,55915 | 0,48769 | 0,33085 | 1 | 0,30998 |
| RPTOR | rs9890464 | 78530810 | normSNP | 0,2587 | C/T | 51,1 | 0,28533 | 0,22455 | 0,30762 | 0,28883 | 0,08417 | 1 |
| RPTOR | rs11655721 | 78530967 | normSNP | 0,2667 | C/G | 50 | 0,1346 | 0,21999 | 0,28883 | 0,28883 | 0,08182 | 1 |
| RPTOR | rs11653158 | 78531256 | normSNP | 0 | G/T | 92,6 | 0,379683 | 1 | 1 | 1 | 0,74706 | 1 |
| RPTOR | rs113898529 | 78531338 | normSNP | 0,1174 | A/C | 68,2 | 0,624407 | 0,03519 | 0,00995 | 0,50044 | 0,0293 | 0,02592 |
| RPTOR | - | 78531342 | normSNP | 0 | A/C | 89,2 | 0 | 0,61815 | 0,33085 | 0,45614 | 0,55323 | 0,36552 |
| RPTOR | rs9897599 | 78531360 | normSNP | 0 | A/G | 94,3 | 0,32747 | 0,50044 | - | - | - | - |
| RPTOR | rs9897762 | 78531361 | normSNP | 0,1185 | A/G | 70,5 | 0,80348 | 0,03625 | 0,01003 | 0,45614 | 0,0293 | 0,02026 |
| RPTOR | rs4480861 | 78531474 | normSNP | 0 | C/T | 93,2 | 0,330711 | 0,7387 | 0,74706 | 1 | 0,50044 | 0,7387 |
| RPTOR | rs9908487 | 78531491 | normSNP | 0 | G/A | 93,2 | 0,330711 | 0,7387 | 0,74706 | 1 | 0,50044 | 0,7387 |
| RPTOR | rs11150863 | 78532321 | normSNP | 0,0793 | G/A | 77,3 | 0,764065 | 0,04104 | 0,01588 | 0,15362 | 0,07072 | 0,01155 |
| RPTOR | rs12452948 | 78532346 | eSNP | 0 | G/A | 79,5 | 0,508429 | 0,01928 | 0,00807 | 0,49425 | 0,02574 | 0,01928 |
| RPTOR | rs11150864 | 78532751 | normSNP | 0 | G/A | 94,3 | 0,32747 | 0,50044 | - | - | - | - |
| RPTOR | rs12451590 | 78532822 | normSNP | 0 | G/A | 94,3 | 0,32747 | 0,50044 | - | - | - | - |
| RPTOR | rs4278795 | 78533189 | normSNP | 0,0818 | A/T | 76,1 | 0,560648 | 0,0919 | 0,0293 | 0,39341 | 0,07072 | 0,03721 |
| RPTOR | rs4297763 | 78533626 | creSNP | 0,1002 | G/C | 76,1 | 0,560648 | 0,0919 | 0,0293 | 0,39341 | 0,07072 | 0,03721 |
| RPTOR | rs9892100 | 78533811 | creSNP | 0,2663 | A/G | 50 | 0,054054 | 0,08858 | 0,17437 | 0,17437 | 0,02768 | 1 |
| RPTOR | rs12452321 | 78533931 | ecreSNP | 0 | A/G | 79,5 | 0,508429 | 0,01928 | 0,00807 | 0,49425 | 0,02574 | 0,01928 |
| RPTOR | rs9905427 | 78534380 | normSNP | 0,1172 | G/T | 69,9 | 0,615285 | 0,03554 | 0,01003 | 0,28706 | 0,04897 | 0,01503 |
| RPTOR | rs9911399 | 78534636 | normSNP | 0,1185 | G/T | 70,5 | 0,80348 | 0,03625 | 0,01003 | 0,45614 | 0,0293 | 0,02026 |
| RPTOR | rs9893668 | 78534640 | normSNP | 0 | T/C | 94,3 | 0,32747 | 0,50044 | - | - | - | - |
| RPTOR | - | 78534727 | normSNP | 0 | A/C | 67 | 0,475736 | 0,01987 | 0,00512 | 0,33085 | 0,0293 | 0,01212 |
| RPTOR | rs67866561 | 78534730 | normSNP | 0,1188 | A/C | 85,2 | 0 | 0,13022 | 0,04367 | 0,10141 | 0,29556 | 0,05415 |
| RPTOR | rs4890044 | 78535273 | normSNP | 0,1016 | G/C | 77,3 | 0,764065 | 0,04104 | 0,01588 | 0,15362 | 0,07072 | 0,01155 |
| RPTOR | rs117273085 | 78535448 | ecreSNP | 0 | G/A | 79,5 | 0,508429 | 0,01928 | 0,00807 | 0,49425 | 0,02574 | 0,01928 |
| RPTOR | rs12603810 | 78535778 | normSNP | 0,118 | G/A | 70,5 | 0,80348 | 0,03625 | 0,01003 | 0,45614 | 0,0293 | 0,02026 |
| RPTOR | rs7210225 | 78536542 | normSNP | 0,3522 | C/T | 59,1 | 0,048985 | 0,57768 | 0,34928 | 0,50044 | 0,66449 | 0,29568 |
| RPTOR | rs11382913 | 78536558 | normSNP | 0 | A/C | 84,1 | 0,522684 | 0,24091 | - | - | - | - |
| RPTOR | rs56873925 | 78536611 | normSNP | 0,0797 | C/T | 76,1 | 0,560648 | 0,0919 | 0,0293 | 0,39341 | 0,07072 | 0,03721 |
| RPTOR | rs12951500 | 78536725 | eSNP | 0 | G/A | 79,5 | 0,508429 | 0,01928 | 0,00807 | 0,49425 | 0,02574 | 0,01928 |
| RPTOR | - | 78537014 | normSNP | 0 | A/C | 84,7 | 0,026078 | 0,29234 | 1 | 0,15362 | 0,41692 | 0,57504 |
| RPTOR | rs7211373 | 78537318 | normSNP | 0 | T/C | 91,5 | 0,012299 | 0,77733 | 0,5336 | 0,55323 | 0,72474 | 0,48282 |
| RPTOR | rs113192259 | 78537385 | normSNP | 0 | A/C | 92,6 | 0,061363 | 0,31845 | 0,74706 | 0,49425 | 0,28706 | 0,31845 |
| RPTOR | rs10871510 | 78537965 | normSNP | 0,1204 | C/T | 65,3 | 0,004048 | 0,09727 | 0,03225 | 0,41692 | 0,10415 | 0,07169 |
| RPTOR | rs12450516 | 78538003 | eSNP | 0 | C/T | 85,2 | 0,682452 | 0,15541 | 0,0963 | 1 | 0,1493 | 0,15541 |
| RPTOR | rs8068274 | 78538405 | normSNP | 0 | T/C | 94,3 | 0,32747 | 0,50044 | - | - | - | - |
| RPTOR | rs11653509 | 78538692 | normSNP | 0,1283 | T/C | 69,9 | 0,615285 | 0,03554 | 0,01003 | 0,28706 | 0,04897 | 0,01503 |
| RPTOR | rs12601815 | 78538875 | eSNP | 0 | C/T | 79 | 0,752823 | 0,01137 | 0,00807 | 0,24138 | 0,04345 | 0,01137 |
| RPTOR | rs7216810 | 78539425 | normSNP | 0 | C/T | 52,3 | 0,001186 | 0,43246 | 0,58001 | 0,21023 | 0,64704 | 0,25344 |
| RPTOR | rs7210150 | 78539594 | normSNP | 0,1328 | A/T | 70,5 | 0,80348 | 0,03625 | 0,01003 | 0,45614 | 0,0293 | 0,02026 |
| RPTOR | rs35108655 | 78539688 | normSNP | 0,2698 | A/G | 51,1 | 0,28533 | 0,22455 | 0,30762 | 0,28883 | 0,08417 | 1 |
| RPTOR | rs7222130 | 78540203 | normSNP | 0,1349 | T/G | 70,5 | 0,80348 | 0,03625 | 0,01003 | 0,45614 | 0,0293 | 0,02026 |
| RPTOR | rs12945242 | 78540832 | eSNP | 0 | A/G | 79 | 0,339731 | 0,02617 | 0,01588 | 0,49425 | 0,04645 | 0,02617 |
| RPTOR | rs75979103 | 78540847 | normSNP | 0,0851 | T/G | 79 | 0,518935 | 0,13496 | 0,07509 | 0,15362 | 0,24675 | 0,04713 |
| RPTOR | rs12939332 | 78540961 | normSNP | 0,1328 | A/G | 68,2 | 0,014052 | 0,20901 | 0,08686 | 0,24091 | 0,35932 | 0,08255 |
| RPTOR | rs9747033 | 78540969 | normSNP | 0 | C/T | 89,8 | 0,000245 | 0,89822 | 0,76375 | 0,64411 | 1 | 0,68468 |
| RPTOR | rs143012748 | 78541464 | eSNP | 0 | C/T | 81,8 | 0,147858 | 0,03176 | 0,01015 | 0,15362 | 0,0469 | 0,00919 |
| RPTOR | rs62068271 | 78541535 | normSNP | 0 | T/C | 92 | 0,082844 | 0,41018 | 1 | 0,49425 | 0,50044 | 0,41018 |
| RPTOR | rs377197917 | 78541603 | normSNP | 0 | A/C | 71,6 | 0,60713 | 0,58743 | 0,66944 | 0,45614 | 0,38076 | 1 |
| RPTOR | rs9907125 | 78541676 | normSNP | 0,133 | T/C | 70,5 | 0,80348 | 0,03625 | 0,01003 | 0,45614 | 0,0293 | 0,02026 |
| RPTOR | rs4890045 | 78541883 | normSNP | 0 | A/G | 92 | 0,007658 | 0,61815 | 0,33085 | 0,55323 | 0,45614 | 0,33817 |
| RPTOR | rs4889858 | 78543155 | normSNP | 0 | T/G | 94,3 | 0,32747 | 0,50044 | - | - | - | - |
| RPTOR | rs4506961 | 78543182 | normSNP | 0,0836 | G/A | 76,7 | 0,548234 | 0,14073 | 0,04897 | 0,39341 | 0,11107 | 0,05634 |
| RPTOR | rs4508465 | 78543368 | normSNP | 0,0849 | G/A | 77,3 | 0,764065 | 0,04104 | 0,01588 | 0,15362 | 0,07072 | 0,01155 |
| RPTOR | rs61096326 | 78543796 | normSNP | 0,0852 | G/C | 76,1 | 0,560648 | 0,0919 | 0,0293 | 0,39341 | 0,07072 | 0,03721 |
| RPTOR | rs8080678 | 78544003 | normSNP | 0,1145 | G/T | 76,1 | 0,560648 | 0,0919 | 0,0293 | 0,39341 | 0,07072 | 0,03721 |
| RPTOR | rs55959137 | 78544330 | normSNP | 0,2366 | G/A | 52,8 | 0,391151 | 0,32191 | 0,32371 | 0,41692 | 0,13218 | 0,87231 |
| RPTOR | rs75398920 | 78544540 | normSNP | 0,0842 | C/T | 76,7 | 1 | 0,13717 | 0,08182 | 0,15362 | 0,26372 | 0,04909 |
| RPTOR | rs12942701 | 78544604 | normSNP | 0 | G/A | 77,3 | 0,543266 | 0,00673 | 0,00463 | 0,24138 | 0,02768 | 0,00673 |
| RPTOR | rs4890046 | 78545548 | normSNP | 0,086 | T/C | 76,1 | 0,560648 | 0,20978 | 0,08182 | 0,39341 | 0,17614 | 0,08363 |
| RPTOR | rs540843850 | 78546214 | normSNP | 0 | A/T | 58 | 0,017237 | 0,52944 | 0,2755 | 0,45249 | 0,65754 | 0,27437 |
| RPTOR | rs8069320 | 78546235 | normSNP | 0,2873 | C/A | 51,1 | 0,134297 | 0,65622 | 0,43624 | 0,7871 | 0,38541 | 0,73866 |
| RPTOR | rs11150865 | 78547433 | eSNP | 0 | C/T | 72,2 | 0,794069 | 0,14777 | 0,05409 | 0,39341 | 0,12969 | 0,05607 |
| RPTOR | rs72856538 | 78547467 | eSNP | 0,077 | G/C | 77,3 | 0,764065 | 0,19152 | 0,51329 | 0,15362 | 0,17614 | 1 |
| RPTOR | rs7226296 | 78547657 | normSNP | 0,286 | G/A | 51,1 | 0,134297 | 0,65622 | 0,43624 | 0,7871 | 0,38541 | 0,73866 |
| RPTOR | rs7215496 | 78547753 | eSNP | 0,0741 | C/T | 71,6 | 0,792807 | 0,09685 | 0,03225 | 0,39341 | 0,08417 | 0,0365 |
| RPTOR | rs12103906 | 78548009 | eSNP | 0,3828 | C/T | 65,3 | 0,246675 | 0,23392 | 0,19823 | 0,12878 | 0,82757 | 0,09814 |
| RPTOR | rs4889861 | 78548569 | normSNP | 0 | C/G | 98,3 | 0,017143 | 1 | 1 | 1 | 1 | 1 |
| RPTOR | rs9899426 | 78549007 | normSNP | 0,2874 | G/A | 51,1 | 0,134297 | 0,65622 | 0,43624 | 0,7871 | 0,38541 | 0,73866 |
| RPTOR | rs4890047 | 78549439 | creSNP | 0,1192 | C/T | 77,3 | 0,764065 | 0,09675 | 0,04897 | 0,15362 | 0,17614 | 0,0312 |
| RPTOR | rs4890048 | 78549602 | normSNP | 0,1102 | G/A | 77,8 | 0,756052 | 0,13646 | 0,07879 | 0,15362 | 0,25593 | 0,04828 |
| RPTOR | rs4396581 | 78549734 | normSNP | 0,2879 | T/C | 51,1 | 0,134297 | 0,65622 | 0,43624 | 0,7871 | 0,38541 | 0,73866 |
| RPTOR | rs11150866 | 78550013 | eSNP | 0 | C/T | 72,2 | 0,794069 | 0,14777 | 0,05409 | 0,39341 | 0,12969 | 0,05607 |
| RPTOR | rs4889863 | 78550468 | normSNP | 0,0859 | A/G | 76,7 | 0,548234 | 0,29037 | 0,12636 | 0,39341 | 0,25593 | 0,11966 |
| RPTOR | rs78012645 | 78550939 | normSNP | 0,0857 | C/T | 79 | 0,518935 | 0,23392 | 0,18267 | 0,15362 | 0,48769 | 0,10571 |
| RPTOR | rs8071934 | 78551583 | normSNP | 0,3677 | C/T | 58,5 | 0,030329 | 0,36324 | 0,47784 | 0,17368 | 0,82757 | 0,21738 |
| RPTOR | rs72856551 | 78552267 | normSNP | 0,087 | G/T | 76,1 | 0,560648 | 0,20978 | 0,08182 | 0,39341 | 0,17614 | 0,08363 |
| RPTOR | - | 78554014 | normSNP | 0 | A/C | 72,2 | 0,432002 | 0,23139 | 0,0872 | 0,64411 | 0,13218 | 0,10795 |
| RPTOR | - | 78554754 | normSNP | 0 | A/C | 77,8 | 0,350089 | 0,4798 | 0,27021 | 0,39341 | 0,48769 | 0,226 |
| RPTOR | rs4424946 | 78554888 | eSNP | 0 | T/C | 72,2 | 0,794069 | 0,14777 | 0,05409 | 0,39341 | 0,12969 | 0,05607 |
| RPTOR | rs11651034 | 78555298 | eSNP | 0,5124 | A/G | 69,9 | 0,800508 | 0,1 | 1 | 0,03876 | 0,28274 | 0,3978 |
| RPTOR | rs11652198 | 78555332 | normSNP | 0,1299 | T/G | 80,1 | 0,036825 | 0,07561 | 1 | 0,03876 | 0,20941 | 0,3974 |
| RPTOR | rs114177791 | 78555512 | eSNP | 0,0622 | G/T | 77,3 | 0,764065 | 0,19152 | 0,51329 | 0,15362 | 0,17614 | 1 |
| RPTOR | rs4075781 | 78556180 | eSNP | 0,5177 | C/T | 65,3 | 0,155603 | 0,10564 | 1 | 0,03876 | 0,28477 | 0,38122 |
| RPTOR | rs12941673 | 78556371 | eSNP | 0 | C/T | 78,4 | 0,53643 | 0,77567 | 0,50866 | 0,64411 | 0,64704 | 0,47601 |
| RPTOR | rs140386936 | 78556635 | normSNP | 0 | C/T | 89,2 | 1 | 0,43624 | - | - | - | - |
| RPTOR | rs376251601 | 78556982 | normSNP | 0 | A/C | 75,6 | 0 | 0,41005 | 0,33749 | 0,19307 | 0,64411 | 0,24369 |
| RPTOR | rs4584879 | 78557050 | eSNP | 0 | G/A | 79 | 0,053112 | 0,06516 | 0,07072 | 0,03876 | 0,46622 | 0,02653 |
| RPTOR | rs4531781 | 78557051 | eSNP | 0,1316 | T/C | 66,5 | 0,000002 | 0,11376 | 0,13218 | 0,03961 | 0,79553 | 0,05017 |
| RPTOR | rs201791426 | 78557379 | normSNP | 0 | A/C | 68,2 | 0,46289 | 0,11714 | 0,51955 | 0,03876 | 0,66944 | 0,17057 |
| RPTOR | rs9899563 | 78557499 | eSNP | 0 | G/A | 72,2 | 0,794069 | 0,14777 | 0,05409 | 0,39341 | 0,12969 | 0,05607 |
| RPTOR | rs35856510 | 78557682 | eSNP | 0 | G/A | 72,2 | 0,794069 | 0,14777 | 0,05409 | 0,39341 | 0,12969 | 0,05607 |
| RPTOR | rs28434589 | 78558082 | eSNP | 0,5197 | G/A | 65,3 | 0,155603 | 0,10564 | 1 | 0,03876 | 0,28477 | 0,38122 |
| RPTOR | rs11655474 | 78558257 | ecreSNP | 0,5183 | C/T | 65,3 | 0,155603 | 0,10564 | 1 | 0,03876 | 0,28477 | 0,38122 |
| RPTOR | rs12951309 | 78558286 | ecreSNP | 0 | G/A | 72,2 | 0,794069 | 0,14777 | 0,05409 | 0,39341 | 0,12969 | 0,05607 |
| RPTOR | rs12949279 | 78558411 | ecreSNP | 0,0917 | T/C | 58 | 0,078597 | 0,05028 | 0,0319 | 0,10141 | 0,38541 | 0,01452 |
| RPTOR | rs12949300 | 78558444 | ecreSNP | 0 | T/C | 72,2 | 0,794069 | 0,14777 | 0,05409 | 0,39341 | 0,12969 | 0,05607 |
| RPTOR | rs62068276 | 78558485 | ecreSNP | 0 | G/A | 85,8 | 0,681984 | 0,47784 | - | - | - | - |
| RPTOR | rs11656563 | 78558542 | ecreSNP | 0,5203 | C/T | 65,3 | 0,155603 | 0,10564 | 1 | 0,03876 | 0,28477 | 0,38122 |
| RPTOR | - | 78558966 | normSNP | 0 | A/C | 95,5 | 0,083296 | 1 | - | - | - | - |
| RPTOR | - | 78558974 | normSNP | 0 | A/C | 89,2 | 1 | 0,42854 | 0,28883 | 1 | 0,41692 | 0,42854 |
| RPTOR | - | 78558994 | normSNP | 0 | A/C | 55,7 | 0,009634 | 0,37963 | 0,20941 | 0,74706 | 0,18267 | 0,47092 |
| RPTOR | - | 78558995 | normSNP | 0 | A/C | 88,1 | 1 | 1 | 1 | 1 | 0,79553 | 1 |
| RPTOR | rs200461858 | 78559020 | normSNP | 0 | T/C | 68,2 | 0,141256 | 0,09147 | 0,03225 | 0,21023 | 0,18267 | 0,03384 |
| RPTOR | rs9902719 | 78559047 | eSNP | 0,5203 | C/G | 70,5 | 0,80348 | 0,31741 | 0,66976 | 0,12995 | 0,66449 | 0,32465 |
| RPTOR | rs4890050 | 78559388 | normSNP | 0 | G/C | 98,9 | 0 | 1 | - | - | - | - |
| RPTOR | rs12600592 | 78559726 | eSNP | 0,5197 | A/G | 65,3 | 0,155603 | 0,10564 | 1 | 0,03876 | 0,28477 | 0,38122 |
| RPTOR | rs12944929 | 78559907 | eSNP | 0 | C/A | 72,2 | 0,794069 | 0,14777 | 0,05409 | 0,39341 | 0,12969 | 0,05607 |
| RPTOR | rs7215020 | 78559908 | eSNP | 0 | G/A | 85,8 | 0,681984 | 0,47784 | - | - | - | - |
| RPTOR | - | 78560663 | normSNP | 0 | A/C | 82,4 | 0,455626 | 0,03582 | 0,01015 | 0,29556 | 0,02736 | 0,01208 |
| RPTOR | rs150091191 | 78561549 | normSNP | 0 | A/C | 72,7 | 0,590502 | 0,15642 | 0,05409 | 0,64411 | 0,08417 | 0,07413 |
| RPTOR | rs7225476 | 78561603 | eSNP | 0,1761 | A/G | 61,9 | 0,173574 | 0,03775 | 0,82902 | 0,02425 | 0,04897 | 0,31136 |
| RPTOR | rs8064502 | 78562434 | eSNP | 0,0917 | C/A | 58 | 0,078597 | 0,05028 | 0,0319 | 0,10141 | 0,38541 | 0,01452 |
| RPTOR | rs12946994 | 78562921 | eSNP | 0 | G/A | 72,2 | 0,794069 | 0,14777 | 0,05409 | 0,39341 | 0,12969 | 0,05607 |
| RPTOR | rs60288601 | 78563151 | eSNP | 0,5285 | A/T | 65,3 | 0,155603 | 0,10564 | 1 | 0,03876 | 0,28477 | 0,38122 |
| RPTOR | rs57262732 | 78563471 | eSNP | 0,5285 | T/C | 65,3 | 0,155603 | 0,10564 | 1 | 0,03876 | 0,28477 | 0,38122 |
| RPTOR | rs4890051 | 78563541 | normSNP | 0 | T | 100 | - | - | - | - | - | - |
| RPTOR | rs7221001 | 78563813 | normSNP | 0 | G/A | 98,9 | 0 | 1 | - | - | - | - |
| RPTOR | rs7221494 | 78564067 | eSNP | 0,1012 | A/G | 61,9 | 0,823526 | 0,02399 | 0,01469 | 0,05731 | 0,28578 | 0,00632 |
| RPTOR | rs11654005 | 78564103 | eSNP | 0,5294 | C/T | 65,3 | 0,155603 | 0,10564 | 1 | 0,03876 | 0,28477 | 0,38122 |
| RPTOR | rs4890052 | 78564245 | normSNP | 0,0943 | G/T | 77,8 | 0,350089 | 0,12629 | 0,12215 | 0,07799 | 0,48769 | 0,05551 |
| RPTOR | rs56260647 | 78564392 | normSNP | 0 | G/A | 71 | 0,008068 | 0,31511 | 0,13218 | 1 | 0,13384 | 0,16375 |
| RPTOR | rs56257075 | 78564395 | normSNP | 0 | C/T | 69,9 | 0,002303 | 0,31102 | 0,12969 | 1 | 0,13218 | 0,16069 |
| RPTOR | rs9904962 | 78564946 | normSNP | 0 | G/A | 98,9 | 0 | 1 | - | - | - | - |
| RPTOR | rs184195122 | 78565042 | normSNP | 0 | C/A | 72,2 | 0,000003 | 0,46656 | 0,27021 | 0,26682 | 0,7871 | 0,21792 |
| RPTOR | rs6420492 | 78566260 | normSNP | 0 | A | 100 | - | - | - | - | - | - |
| RPTOR | rs4303599 | 78566700 | normSNP | 0,0944 | C/T | 77,8 | 0,756052 | 0,13646 | 0,07879 | 0,15362 | 0,25593 | 0,04828 |
| RPTOR | rs56698771 | 78567750 | normSNP | 0,3748 | G/A | 56,8 | 0,385763 | 1 | 1 | 1 | 1 | 1 |
| RPTOR | rs62068297 | 78567775 | eSNP | 0 | C/T | 86,9 | 1 | 0,46622 | - | - | - | - |
| RPTOR | rs4889865 | 78568447 | normSNP | 0,0942 | A/T | 76,1 | 0,560648 | 0,20978 | 0,08182 | 0,39341 | 0,17614 | 0,08363 |
| RPTOR | - | 78568836 | normSNP | 0 | A/C | 59,1 | 0,000153 | 0,09479 | 0,32371 | 0,07799 | 0,06568 | 1 |
| RPTOR | - | 78569259 | normSNP | 0 | A/C | 72,2 | 0,794069 | 0,14777 | 0,05409 | 0,39341 | 0,12969 | 0,05607 |
| RPTOR | rs6565701 | 78569900 | normSNP | 0 | T | 100 | - | - | - | - | - | - |
| RPTOR | rs12952925 | 78569948 | eSNP | 0 | A/G | 72,7 | 0,590502 | 0,15642 | 0,05409 | 0,64411 | 0,08417 | 0,07413 |
| RPTOR | - | 78569977 | normSNP | 0 | A/C | 83 | 0,707919 | 0,11131 | 0,03613 | 0,55323 | 0,05373 | 0,04579 |
| RPTOR | rs11150867 | 78570504 | normSNP | 0 | G | 100 | - | - | - | - | - | - |
| RPTOR | rs12450644 | 78570973 | ecreSNP | 0 | G/A | 72,2 | 0,794069 | 0,14777 | 0,05409 | 0,39341 | 0,12969 | 0,05607 |
| RPTOR | rs12450647 | 78571042 | ecreSNP | 0,0693 | G/T | 72,2 | 0,794069 | 0,14777 | 0,05409 | 0,39341 | 0,12969 | 0,05607 |
| RPTOR | rs8065287 | 78571226 | ecreSNP | 0,0693 | G/C | 72,2 | 0,794069 | 0,14777 | 0,05409 | 0,39341 | 0,12969 | 0,05607 |
| RPTOR | rs147979011 | 78571466 | eSNP | 0,527 | A/C | 64,2 | 0,064645 | 0,1068 | 1 | 0,03876 | 0,28274 | 0,37514 |
| RPTOR | rs12948413 | 78571796 | eSNP | 0 | G/T | 72,2 | 0,794069 | 0,14777 | 0,05409 | 0,39341 | 0,12969 | 0,05607 |
| RPTOR | rs9891172 | 78572131 | eSNP | 0,0668 | A/G | 72,7 | 0,590502 | 0,15642 | 0,05409 | 0,64411 | 0,08417 | 0,07413 |
| RPTOR | - | 78572682 | normSNP | 0 | A/C | 57,4 | 0,015774 | 0,25164 | 0,22367 | 0,17368 | 0,82571 | 0,10408 |
| RPTOR | - | 78573129 | normSNP | 0 | A/C | 77,3 | 0,764065 | 0,2892 | 0,82757 | 0,15362 | 0,36782 | 0,72283 |
| RPTOR | rs9913181 | 78573872 | creSNP | 0,1009 | T/G | 66,5 | 0,232728 | 0,2818 | 0,19248 | 0,23023 | 0,52216 | 0,11765 |
| RPTOR | rs9901467 | 78574008 | creSNP | 0,1005 | A/G | 76,1 | 0,560648 | 0,20978 | 0,08182 | 0,39341 | 0,17614 | 0,08363 |
| RPTOR | - | 78574088 | normSNP | 0 | A/C | 83 | 0,263109 | 0,15421 | 0,06001 | 0,29556 | 0,1377 | 0,0543 |
| RPTOR | rs35524093 | 78574246 | eSNP | 0,0975 | A/G | 66,5 | 0,232728 | 0,2818 | 0,19248 | 0,23023 | 0,52216 | 0,11765 |
| RPTOR | rs9902390 | 78574387 | eSNP | 0,0948 | G/A | 66,5 | 0,232728 | 0,2818 | 0,19248 | 0,23023 | 0,52216 | 0,11765 |
| RPTOR | rs113056888 | 78574660 | eSNP | 0,5269 | G/A | 64,2 | 0,064645 | 0,1068 | 1 | 0,03876 | 0,28274 | 0,37514 |
| RPTOR | rs7209768 | 78574717 | normSNP | 0,4747 | G/A | 70,5 | 1 | 0,10305 | 0,28578 | 0,03876 | 1 | 0,09012 |
| RPTOR | rs62068300 | 78574727 | eSNP | 0,4905 | G/A | 65,3 | 0,155603 | 0,11814 | 0,66142 | 0,03876 | 0,5213 | 0,21946 |
| RPTOR | rs150267076 | 78575082 | normSNP | 0 | A/C | 51,1 | 0,005175 | 0,08364 | 0,09388 | 0,07623 | 1 | 0,02617 |
| RPTOR | rs7225687 | 78575103 | normSNP | 0 | C/T | 65,9 | 0,000002 | 0,35932 | - | - | - | - |
| RPTOR | - | 78575353 | normSNP | 0 | A/C | 71,6 | 0,000465 | 0,83375 | 0,66449 | 0,55939 | 1 | 0,56975 |
| RPTOR | rs12451164 | 78576194 | normSNP | 0 | C/T | 71 | 0 | 0,17898 | 0,65278 | 0,45249 | 0,07105 | 0,90009 |
| RPTOR | rs112172082 | 78576212 | normSNP | 0 | G/A | 76,7 | 0 | 0,07675 | 0,02736 | 0,03199 | 0,64411 | 0,02352 |
| RPTOR | rs4890053 | 78576248 | normSNP | 0 | G/A | 67 | 0 | 0,14171 | 0,07879 | 0,05373 | 1 | 0,05051 |
| RPTOR | - | 78576270 | normSNP | 0 | A/C | 81,8 | 0,00093 | 0,75282 | 0,63199 | 0,45614 | 1 | 0,5064 |
| RPTOR | rs12449366 | 78576347 | eSNP | 0 | G/A | 72,7 | 0,590502 | 0,15642 | 0,05409 | 0,64411 | 0,08417 | 0,07413 |
| RPTOR | rs12451045 | 78576652 | eSNP | 0 | G/A | 72,7 | 0,590502 | 0,15642 | 0,05409 | 0,64411 | 0,08417 | 0,07413 |
| RPTOR | rs12946618 | 78577168 | eSNP | 0 | G/A | 72,2 | 0,432002 | 0,10077 | 0,03225 | 0,64411 | 0,05255 | 0,04879 |
| RPTOR | rs7221617 | 78577351 | normSNP | 0 | G | 100 | - | - | - | - | - | - |
| RPTOR | rs12937297 | 78577638 | eSNP | 0 | G/A | 72,2 | 0,794069 | 0,14777 | 0,05409 | 0,39341 | 0,12969 | 0,05607 |
| RPTOR | rs11869826 | 78578153 | eSNP | 0,4799 | G/A | 64,2 | 0,064645 | 0,1068 | 1 | 0,03876 | 0,28274 | 0,37514 |
| RPTOR | rs11867812 | 78578154 | eSNP | 0,4799 | A/T | 64,2 | 0,064645 | 0,1068 | 1 | 0,03876 | 0,28274 | 0,37514 |
| RPTOR | rs2315927 | 78578898 | normSNP | 0,094 | C/T | 76,1 | 0,560648 | 0,20978 | 0,08182 | 0,39341 | 0,17614 | 0,08363 |
| RPTOR | rs7218776 | 78579213 | ecreSNP | 0,1727 | T/C | 69,9 | 0,012048 | 0,25126 | 0,19823 | 0,12878 | 0,81718 | 0,10694 |
| RPTOR | rs9899898 | 78579283 | ecreSNP | 0,4847 | G/A | 65,9 | 0,059461 | 0,03594 | 1 | 0,02605 | 0,19823 | 0,03594 |
| RPTOR | rs138907285 | 78580580 | normSNP | 0,2848 | A/C | 87,5 | 1 | 1 | 0,80249 | 1 | 1 | 1 |
| RPTOR | - | 78581396 | normSNP | 0 | A/C | 79,5 | 0,000001 | 0,10662 | 0,05373 | 0,05731 | 0,5336 | 0,03468 |
| RPTOR | - | 78581399 | normSNP | 0 | A/C | 67,6 | 0,463839 | 0,2194 | 0,08686 | 0,74706 | 0,12636 | 0,16359 |
| RPTOR | rs4477776 | 78581765 | normSNP | 0,0985 | A/G | 76,7 | 0,548234 | 0,29037 | 0,12636 | 0,39341 | 0,25593 | 0,11966 |
| RPTOR | rs8080957 | 78582693 | eSNP | 0,3738 | G/A | 57,4 | 0,275261 | 0,95246 | 1 | 0,76375 | 0,83008 | 0,86955 |
| RPTOR | rs141872693 | 78583573 | normSNP | 0 | A/C | 56,2 | 0,518094 | 0,23464 | 0,64017 | 0,15304 | 0,13384 | 0,6335 |
| RPTOR | rs12942637 | 78583871 | eSNP | 0 | T/C | 71,6 | 0,792807 | 0,09685 | 0,03225 | 0,39341 | 0,08417 | 0,0365 |
| RPTOR | rs12945061 | 78583954 | eSNP | 0 | G/C | 71,6 | 0,792807 | 0,09685 | 0,03225 | 0,39341 | 0,08417 | 0,0365 |
| RPTOR | rs11653499 | 78584303 | eSNP | 0,5375 | G/A | 65,9 | 0,059461 | 0,03594 | 1 | 0,02605 | 0,19823 | 0,03594 |
| RPTOR | rs146259280 | 78585569 | normSNP | 0 | A/C | 54 | 0,057346 | 0,046 | 0,20941 | 0,07623 | 0,01588 | 0,86469 |
| RPTOR | rs12452339 | 78585608 | eSNP | 0 | G/A | 71,6 | 0,792807 | 0,09685 | 0,03225 | 0,39341 | 0,08417 | 0,0365 |
| RPTOR | rs35895230 | 78585732 | eSNP | 0 | A/G | 71,6 | 0,792807 | 0,09685 | 0,03225 | 0,39341 | 0,08417 | 0,0365 |
| RPTOR | rs12946802 | 78587740 | eSNP | 0 | T/C | 71 | 0,607314 | 0,06018 | 0,01836 | 0,39341 | 0,05255 | 0,02294 |
| RPTOR | rs12942347 | 78587942 | normSNP | 0,0735 | C/T | 63,6 | 0,040873 | 0,1976 | 0,11706 | 0,23023 | 0,38896 | 0,07244 |
| RPTOR | rs12939613 | 78588202 | eSNP | 0,0985 | A/G | 56,8 | 0,081949 | 0,02082 | 0,01544 | 0,05731 | 0,38541 | 0,00542 |
| RPTOR | rs8074235 | 78588669 | normSNP | 0 | C | 100 | - | - | - | - | - | - |
| RPTOR | rs12951779 | 78588726 | eSNP | 0 | C/T | 71 | 0,607314 | 0,06018 | 0,01836 | 0,39341 | 0,05255 | 0,02294 |
| RPTOR | rs35881344 | 78588926 | normSNP | 0,3035 | G/A | 88,1 | 1 | 0,446 | 0,30762 | 1 | 0,43624 | 0,446 |
| RPTOR | rs12451459 | 78589668 | normSNP | 0,0924 | G/A | 76,7 | 0,548234 | 0,29037 | 0,12636 | 0,39341 | 0,25593 | 0,11966 |
| RPTOR | rs8072291 | 78590217 | eSNP | 0,5545 | G/T | 69,9 | 0,131442 | 0,62254 | 0,66944 | 0,33085 | 0,8234 | 0,44561 |
| RPTOR | rs7503186 | 78590472 | normSNP | 0,0702 | G/C | 64,2 | 0,064645 | 0,14509 | 0,07509 | 0,23023 | 0,28274 | 0,04943 |
| RPTOR | rs7503203 | 78590528 | eSNP | 0,0722 | G/A | 64,8 | 0,034284 | 0,18062 | 0,07509 | 0,39341 | 0,19585 | 0,06724 |
| RPTOR | rs72858219 | 78590588 | normSNP | 0,0842 | G/A | 78,4 | 1 | 0,07869 | 0,66142 | 0,11643 | 0,17614 | 0,07869 |
| RPTOR | rs79641221 | 78590792 | normSNP | 0,3237 | A/G | 88,1 | 1 | 0,446 | 0,30762 | 1 | 0,43624 | 0,446 |
| RPTOR | rs9890502 | 78591025 | eSNP | 0,5552 | C/T | 66,5 | 0,09343 | 0,03829 | 0,82757 | 0,02605 | 0,28477 | 0,03829 |
| RPTOR | rs7503807 | 78591111 | eSNP | 0,1321 | A/C | 56,8 | 0,081949 | 0,02082 | 0,01544 | 0,05731 | 0,38541 | 0,00542 |
| RPTOR | rs4890056 | 78591211 | eSNP | 0,0983 | T/C | 56,8 | 0,081949 | 0,02082 | 0,01544 | 0,05731 | 0,38541 | 0,00542 |
| RPTOR | rs9909241 | 78591427 | eSNP | 0,4799 | T/G | 66,5 | 0,09343 | 0,03829 | 0,82757 | 0,02605 | 0,28477 | 0,03829 |
| RPTOR | rs4890057 | 78591679 | normSNP | 0,1001 | G/A | 76,1 | 0,560648 | 0,20978 | 0,08182 | 0,39341 | 0,17614 | 0,08363 |
| RPTOR | rs12936815 | 78591874 | eSNP | 0,0933 | G/A | 58,5 | 0,389541 | 0,06732 | 0,02101 | 0,36558 | 0,13384 | 0,03426 |
| RPTOR | rs12937163 | 78592057 | eSNP | 0 | G/A | 71 | 0,607314 | 0,06018 | 0,01836 | 0,39341 | 0,05255 | 0,02294 |
| RPTOR | rs11651320 | 78592121 | eSNP | 0,4817 | C/T | 66,5 | 0,09343 | 0,03829 | 0,82757 | 0,02605 | 0,28477 | 0,03829 |
| RPTOR | rs4889775 | 78592287 | eSNP | 0,1337 | A/G | 56,8 | 0,081949 | 0,02082 | 0,01544 | 0,05731 | 0,38541 | 0,00542 |
| RPTOR | rs9899250 | 78592367 | eSNP | 0,4811 | C/T | 66,5 | 0,09343 | 0,03829 | 0,82757 | 0,02605 | 0,28477 | 0,03829 |
| RPTOR | rs4889867 | 78593058 | eSNP | 0,1321 | C/T | 58 | 0,078597 | 0,05028 | 0,0319 | 0,10141 | 0,38541 | 0,01452 |
| RPTOR | rs12940468 | 78594514 | eSNP | 0 | C/T | 72,2 | 0,432002 | 0,10077 | 0,03225 | 0,64411 | 0,05255 | 0,04879 |
| RPTOR | rs11658093 | 78594534 | eSNP | 0,4866 | T/C | 65,9 | 0,059461 | 0,03594 | 1 | 0,02605 | 0,19823 | 0,03594 |
| RPTOR | rs12946049 | 78594669 | eSNP | 0 | T/C | 71,6 | 0,792807 | 0,09685 | 0,03225 | 0,39341 | 0,08417 | 0,0365 |
| RPTOR | rs901060 | 78594990 | normSNP | 0,1068 | G/A | 64,8 | 0,101146 | 0,20616 | 0,12215 | 0,23023 | 0,39146 | 0,07647 |
| RPTOR | rs901061 | 78595328 | normSNP | 0,1051 | G/C | 64,8 | 0,101146 | 0,20616 | 0,12215 | 0,23023 | 0,39146 | 0,07647 |
| RPTOR | rs12950489 | 78595542 | eSNP | 0 | C/T | 71,6 | 0,792807 | 0,09685 | 0,03225 | 0,39341 | 0,08417 | 0,0365 |
| RPTOR | rs34019285 | 78595585 | eSNP | 0 | G/A | 71,6 | 0,792807 | 0,09685 | 0,03225 | 0,39341 | 0,08417 | 0,0365 |
| RPTOR | rs901062 | 78595674 | normSNP | 0,1049 | C/T | 65,3 | 0,057509 | 0,26095 | 0,12215 | 0,39341 | 0,28274 | 0,10232 |
| RPTOR | rs35081961 | 78595747 | eSNP | 0 | G/A | 72,2 | 0,432002 | 0,10077 | 0,03225 | 0,64411 | 0,05255 | 0,04879 |
| RPTOR | rs901064 | 78596040 | eSNP | 0,137 | A/G | 57,4 | 0,048794 | 0,02834 | 0,01544 | 0,10141 | 0,2755 | 0,00795 |
| RPTOR | rs12952285 | 78596158 | normSNP | 0,1051 | C/G | 64,8 | 0,101146 | 0,20616 | 0,12215 | 0,23023 | 0,39146 | 0,07647 |
| RPTOR | rs12942880 | 78596435 | eSNP | 0 | G/A | 71,6 | 0,792807 | 0,09685 | 0,03225 | 0,39341 | 0,08417 | 0,0365 |
| RPTOR | rs12947696 | 78596793 | eSNP | 0,1357 | C/T | 57,4 | 0,048794 | 0,02834 | 0,01544 | 0,10141 | 0,2755 | 0,00795 |
| RPTOR | rs62068310 | 78597054 | eSNP | 0,4882 | T/C | 65,9 | 0,059461 | 0,03594 | 1 | 0,02605 | 0,19823 | 0,03594 |
| RPTOR | rs4889870 | 78597360 | normSNP | 0,1049 | G/A | 64,8 | 0,101146 | 0,20616 | 0,12215 | 0,23023 | 0,39146 | 0,07647 |
| RPTOR | rs4889871 | 78597399 | normSNP | 0,1061 | C/A | 64,8 | 0,101146 | 0,20616 | 0,12215 | 0,23023 | 0,39146 | 0,07647 |
| RPTOR | rs9905747 | 78597524 | normSNP | 0,1049 | C/T | 77,3 | 1 | 0,40141 | 0,19248 | 1 | 0,18267 | 0,27035 |
| RPTOR | rs9906589 | 78597551 | normSNP | 0,1048 | A/G | 75 | 0,570228 | 0,18353 | 0,08586 | 0,29556 | 0,19248 | 0,06573 |
| RPTOR | rs28719636 | 78597927 | eSNP | 0,4893 | A/G | 65,9 | 0,059461 | 0,03594 | 1 | 0,02605 | 0,19823 | 0,03594 |
| RPTOR | rs7209279 | 78598055 | normSNP | 0,1008 | A/T | 76,7 | 0,228113 | 0,31187 | 0,1881 | 0,23023 | 0,48769 | 0,13057 |
| RPTOR | rs7220588 | 78598231 | normSNP | 0,1008 | A/G | 75,6 | 0,771737 | 0,29326 | 0,12969 | 0,39341 | 0,26372 | 0,1203 |
| RPTOR | rs7209723 | 78598339 | eSNP | 0,5362 | G/C | 72,7 | 0,016284 | 0,25792 | 0,39295 | 0,49425 | 0,20012 | 0,25792 |
| RPTOR | rs9914854 | 78598662 | normSNP | 0,1008 | G/A | 76,7 | 0,548234 | 0,29037 | 0,12636 | 0,39341 | 0,25593 | 0,11966 |
| RPTOR | rs731452 | 78599052 | normSNP | 0,0749 | G/T | 64,8 | 0,101146 | 0,20616 | 0,12215 | 0,23023 | 0,39146 | 0,07647 |
| RPTOR | rs9319606 | 78599067 | normSNP | 0,2646 | T/G | 58,5 | 0,389541 | 0,95205 | 1 | 0,76375 | 0,83078 | 0,87186 |
| RPTOR | rs17848685 | 78599562 | normSNP | 0 | C/G | 77,8 | 1 | 0,05982 | 0,51329 | 0,11643 | 0,11706 | 0,05982 |
| RPTOR | rs901065 | 78599655 | creSNP | 0,0952 | G/T | 65,9 | 0,059461 | 0,35386 | 0,1881 | 0,39341 | 0,39146 | 0,14947 |
| RPTOR | rs8078454 | 78599847 | eSNP | 0,5196 | T/A | 65,9 | 0,059461 | 0,03594 | 1 | 0,02605 | 0,19823 | 0,03594 |
| RPTOR | rs58973922 | 78600065 | eSNP | 0,5196 | A/G | 84,1 | 0,217546 | 0,18053 | 0,33749 | 0,11643 | 1 | 0,18053 |
| RPTOR | rs542887031 | 78600152 | normSNP | 0 | A/C | 88,1 | 0 | 0,31409 | 0,33085 | 0,17368 | 1 | 0,31409 |
| RPTOR | rs112482676 | 78600155 | normSNP | 0,3007 | A/C | 89,8 | 0,280048 | 0,28706 | - | - | - | - |
| RPTOR | rs561149743 | 78600156 | normSNP | 0 | A/C | 89,2 | 0 | 0,61815 | 0,33085 | 0,45614 | 0,55323 | 0,36552 |
| RPTOR | rs4889779 | 78600534 | normSNP | 0 | A/T | 59,7 | 0 | 0,42854 | 0,28883 | 1 | 0,43624 | 0,42854 |
| RPTOR | rs8077626 | 78600552 | normSNP | 0,1017 | C/G | 76,1 | 0,560648 | 0,20978 | 0,08182 | 0,39341 | 0,17614 | 0,08363 |
| RPTOR | rs61213659 | 78600783 | eSNP | 0,5207 | C/T | 65,9 | 0,059461 | 0,03594 | 1 | 0,02605 | 0,19823 | 0,03594 |
| RPTOR | rs8079537 | 78600818 | eSNP | 0,1353 | A/G | 57,4 | 0,048794 | 0,02834 | 0,01544 | 0,10141 | 0,2755 | 0,00795 |
| RPTOR | - | 78601165 | normSNP | 0 | A/C | 73,3 | 1 | 0,14785 | 0,05358 | 0,39341 | 0,12636 | 0,05667 |
| RPTOR | rs12936076 | 78601195 | eSNP | 0,131 | C/T | 57,4 | 0,048794 | 0,02834 | 0,01544 | 0,10141 | 0,2755 | 0,00795 |
| RPTOR | rs8071015 | 78602372 | eSNP | 0,5558 | A/C | 65,9 | 0,059461 | 0,03594 | 1 | 0,02605 | 0,19823 | 0,03594 |
| RPTOR | rs12600403 | 78602477 | normSNP | 0 | A/T | 64,8 | 0,101146 | 0,20616 | 0,12215 | 0,23023 | 0,39146 | 0,07647 |
| RPTOR | rs11654265 | 78602697 | creSNP | 0,0938 | G/A | 76,7 | 0,548234 | 0,29037 | 0,12636 | 0,39341 | 0,25593 | 0,11966 |
| RPTOR | rs9891324 | 78602899 | normSNP | 0 | A/C | 86,9 | 0,00043 | 0,04079 | 0,17437 | 0,02605 | 0,74706 | 0,04079 |
| RPTOR | rs60268947 | 78603006 | ecreSNP | 0,563 | G/A | 65,9 | 0,059461 | 0,03594 | 1 | 0,02605 | 0,19823 | 0,03594 |
| RPTOR | rs7213432 | 78603090 | creSNP | 0 | A/G | 93,2 | 0,330711 | 0,01492 | 0,01951 | 1 | 0,00423 | 0,01492 |
| RPTOR | rs56209503 | 78603142 | creSNP | 0,077 | C/T | 71,6 | 0,792807 | 0,56297 | 0,39344 | 0,39341 | 0,66683 | 0,29936 |
| RPTOR | rs56137145 | 78603168 | creSNP | 0,0629 | C/T | 72,2 | 0,794069 | 0,06059 | 0,01836 | 0,39341 | 0,05101 | 0,02347 |
| RPTOR | - | 78603385 | normSNP | 0 | A/C | 78,4 | 1 | 0,18741 | 0,07879 | 1 | 0,07072 | 0,13899 |
| RPTOR | - | 78603594 | normSNP | 0 | A/C | 72,7 | 0,590502 | 0,06141 | 0,01836 | 0,64411 | 0,03058 | 0,03156 |
| RPTOR | rs7220261 | 78604161 | eSNP | 0 | C/G | 72,2 | 0,432002 | 0,10077 | 0,03225 | 0,64411 | 0,05255 | 0,04879 |
| RPTOR | rs7225153 | 78604275 | eSNP | 0 | T/C | 71,6 | 0,792807 | 0,09685 | 0,03225 | 0,39341 | 0,08417 | 0,0365 |
| RPTOR | rs8080265 | 78604814 | eSNP | 0,5186 | G/A | 65,9 | 0,059461 | 0,03594 | 1 | 0,02605 | 0,19823 | 0,03594 |
| RPTOR | rs8082303 | 78605012 | normSNP | 0,1016 | G/A | 76,1 | 0,560648 | 0,20978 | 0,08182 | 0,39341 | 0,17614 | 0,08363 |
| RPTOR | rs12948080 | 78606129 | ecreSNP | 0 | G/C | 71,6 | 0,792807 | 0,09685 | 0,03225 | 0,39341 | 0,08417 | 0,0365 |
| RPTOR | rs12952775 | 78607820 | normSNP | 0,1031 | G/A | 76,7 | 0,548234 | 0,29037 | 0,12636 | 0,39341 | 0,25593 | 0,11966 |
| RPTOR | - | 78607918 | normSNP | 0 | A/C | 58,5 | 1 | 0,05843 | 0,02351 | 0,15304 | 0,28578 | 0,01994 |
| RPTOR | rs62068345 | 78608040 | normSNP | 0,1298 | C/T | 75,6 | 0,00021 | 0,39633 | 0,26372 | 0,21023 | 0,79553 | 0,18238 |
| RPTOR | rs62068346 | 78608052 | normSNP | 0,1013 | G/A | 69,3 | 0 | 0,19341 | 0,12636 | 0,79553 | 0,09388 | 0,29609 |
| RPTOR | rs62641966 | 78608075 | normSNP | 0,1296 | G/A | 71,6 | 0,186482 | 0,68282 | 0,39295 | 1 | 0,39295 | 0,46162 |
| RPTOR | rs202244249 | 78608105 | normSNP | 0 | G/A | 77,8 | 0,545987 | 0,40776 | 0,19248 | 0,55323 | 0,27021 | 0,18172 |
| RPTOR | rs62648832 | 78608289 | normSNP | 0,0957 | A/G | 69,9 | 0,131442 | 0,2044 | 0,19965 | 0,10141 | 0,8234 | 0,0917 |
| RPTOR | rs201831890 | 78608291 | normSNP | 0 | A/C | 88,1 | 0,016486 | 0,95548 | 0,7871 | 1 | 0,76375 | 0,83818 |
| RPTOR | - | 78608397 | normSNP | 0 | A/C | 88,1 | 0,000004 | 0,84335 | 0,55939 | 0,69315 | 0,69315 | 0,58307 |
| RPTOR | rs560638674 | 78608403 | normSNP | 0,0997 | A/C | 72,7 | 0,683252 | 0,33749 | - | - | - | - |
| RPTOR | - | 78608657 | normSNP | 0 | A/C | 64,2 | 0 | 0,24735 | 0,51329 | 0,16844 | 0,23023 | 0,29439 |
| RPTOR | rs62068351 | 78608933 | eSNP | 0,5499 | C/G | 65,9 | 0,059461 | 0,03594 | 1 | 0,02605 | 0,19823 | 0,03594 |
| RPTOR | rs8079792 | 78609394 | normSNP | 0,1032 | G/A | 76,1 | 0,560648 | 0,20978 | 0,08182 | 0,39341 | 0,17614 | 0,08363 |
| RPTOR | rs35327093 | 78609965 | eSNP | 0 | C/T | 85,8 | 0,681984 | 0,47784 | - | - | - | - |
| RPTOR | rs6565467 | 78610113 | normSNP | 0,1032 | G/A | 76,7 | 0,548234 | 0,29037 | 0,12636 | 0,39341 | 0,25593 | 0,11966 |
| RPTOR | rs7501740 | 78610345 | normSNP | 0,1034 | G/A | 76,7 | 0,548234 | 0,29037 | 0,12636 | 0,39341 | 0,25593 | 0,11966 |
| RPTOR | rs9910745 | 78610543 | eSNP | 0,1341 | C/T | 58,5 | 0,009097 | 0,02135 | 0,01544 | 0,07105 | 0,27021 | 0,00554 |
| RPTOR | rs12939549 | 78611724 | eSNP | 0,1338 | A/G | 57,4 | 0,048794 | 0,02834 | 0,01544 | 0,10141 | 0,2755 | 0,00795 |
| RPTOR | rs10163471 | 78611941 | eSNP | 0,5483 | G/A | 65,9 | 0,059461 | 0,03594 | 1 | 0,02605 | 0,19823 | 0,03594 |
| RPTOR | rs7215486 | 78612034 | eSNP | 0,1323 | C/T | 57,4 | 0,048794 | 0,02834 | 0,01544 | 0,10141 | 0,2755 | 0,00795 |
| RPTOR | rs7215534 | 78612119 | eSNP | 0,1329 | C/T | 57,4 | 0,048794 | 0,02834 | 0,01544 | 0,10141 | 0,2755 | 0,00795 |
| RPTOR | rs12940068 | 78612956 | eSNP | 0 | T/C | 71,6 | 0,792807 | 0,09685 | 0,03225 | 0,39341 | 0,08417 | 0,0365 |
| RPTOR | rs11150735 | 78613486 | eSNP | 0 | C/G | 71 | 0,607314 | 0,14657 | 0,05409 | 0,39341 | 0,13218 | 0,05511 |
| RPTOR | rs11150736 | 78613507 | eSNP | 0,1082 | C/T | 65,3 | 0,057509 | 0,26095 | 0,12215 | 0,39341 | 0,28274 | 0,10232 |
| RPTOR | rs9915162 | 78614326 | eSNP | 0,1309 | T/C | 58,5 | 0,030329 | 0,06614 | 0,0319 | 0,17368 | 0,2755 | 0,02074 |
| RPTOR | rs9909449 | 78614393 | eSNP | 0,1309 | A/G | 58 | 0,380514 | 0,1428 | 0,10415 | 0,12878 | 0,66847 | 0,04894 |
| RPTOR | rs8068637 | 78614470 | eSNP | 0,1104 | C/T | 64,8 | 0,101146 | 0,20616 | 0,12215 | 0,23023 | 0,39146 | 0,07647 |
| RPTOR | - | 78614596 | normSNP | 0 | A/C | 66,5 | 0,09343 | 0,03829 | 0,82757 | 0,02605 | 0,28477 | 0,03829 |
| RPTOR | rs8072592 | 78615361 | normSNP | 0,1017 | T/G | 76,7 | 0,548234 | 0,29037 | 0,12636 | 0,39341 | 0,25593 | 0,11966 |
| RPTOR | rs12940622 | 78615571 | eSNP | 0,1302 | G/A | 57,4 | 0,048794 | 0,02834 | 0,01544 | 0,10141 | 0,2755 | 0,00795 |
| RPTOR | rs8073414 | 78615899 | normSNP | 0,1804 | A/G | 69,3 | 1 | 0,28439 | 1 | 0,12995 | 0,38896 | 0,50961 |
| RPTOR | rs35418252 | 78615982 | eSNP | 0 | G/A | 71,6 | 0,792807 | 0,09685 | 0,03225 | 0,39341 | 0,08417 | 0,0365 |
| RPTOR | rs8074714 | 78616019 | eSNP | 0,563 | C/T | 65,9 | 0,059461 | 0,03594 | 1 | 0,02605 | 0,19823 | 0,03594 |
| RPTOR | rs6565469 | 78617439 | normSNP | 0,1032 | C/T | 76,7 | 0,548234 | 0,29037 | 0,12636 | 0,39341 | 0,25593 | 0,11966 |
| RPTOR | rs1006809 | 78617956 | eSNP | 0,1543 | T/C | 69,3 | 0,005339 | 0,18191 | 0,19823 | 0,07623 | 1 | 0,0841 |
| RPTOR | rs12603265 | 78618637 | normSNP | 0,1032 | G/A | 76,1 | 0,560648 | 0,20978 | 0,08182 | 0,39341 | 0,17614 | 0,08363 |
| RPTOR | rs12946972 | 78618922 | normSNP | 0,1032 | C/T | 76,1 | 0,560648 | 0,20978 | 0,08182 | 0,39341 | 0,17614 | 0,08363 |
| RPTOR | rs12936687 | 78619009 | normSNP | 0,1489 | A/G | 69,3 | 0,005339 | 0,18191 | 0,19823 | 0,07623 | 1 | 0,0841 |
| RPTOR | rs58272260 | 78619050 | eSNP | 0,3783 | C/T | 65,9 | 0,059461 | 0,03594 | 1 | 0,02605 | 0,19823 | 0,03594 |
| RPTOR | rs12947901 | 78619242 | eSNP | 0 | T/C | 71,6 | 0,792807 | 0,09685 | 0,03225 | 0,39341 | 0,08417 | 0,0365 |
| RPTOR | rs1007850 | 78619434 | normSNP | 0,1514 | C/T | 69,3 | 0,005339 | 0,18191 | 0,19823 | 0,07623 | 1 | 0,0841 |
| RPTOR | rs8066867 | 78620072 | eSNP | 0,5207 | G/A | 65,9 | 0,059461 | 0,03594 | 1 | 0,02605 | 0,19823 | 0,03594 |
| RPTOR | rs8067046 | 78620158 | eSNP | 0,5207 | G/T | 65,9 | 0,059461 | 0,03594 | 1 | 0,02605 | 0,19823 | 0,03594 |
| RPTOR | rs7221014 | 78620957 | normSNP | 0,1482 | C/T | 69,3 | 0,005339 | 0,18191 | 0,19823 | 0,07623 | 1 | 0,0841 |
| RPTOR | rs4889872 | 78621306 | normSNP | 0,0907 | G/A | 77,3 | 0,764065 | 0,09675 | 0,04897 | 0,15362 | 0,17614 | 0,0312 |
| RPTOR | rs9900877 | 78621328 | normSNP | 0,1478 | C/T | 69,3 | 0,005339 | 0,18191 | 0,19823 | 0,07623 | 1 | 0,0841 |
| RPTOR | rs78638495 | 78621470 | creSNP | 0,32 | G/A | 88,1 | 1 | 0,446 | 0,30762 | 1 | 0,43624 | 0,446 |
| RPTOR | rs8077832 | 78621625 | creSNP | 0,1477 | T/C | 69,3 | 0,005339 | 0,18191 | 0,19823 | 0,07623 | 1 | 0,0841 |
| RPTOR | rs374798455 | 78621626 | normSNP | 0 | T/G | 69,3 | 0,005339 | 0,18191 | 0,19823 | 0,07623 | 1 | 0,0841 |
| RPTOR | rs8076974 | 78621817 | normSNP | 0,1002 | A/G | 76,1 | 0,560648 | 0,20978 | 0,08182 | 0,39341 | 0,17614 | 0,08363 |
| RPTOR | rs8064620 | 78622182 | normSNP | 0,1373 | G/T | 68,8 | 0,011578 | 0,19924 | 0,28477 | 0,07623 | 0,81718 | 0,11282 |
| RPTOR | rs8079156 | 78622217 | normSNP | 0,1408 | G/A | 68,8 | 0,011578 | 0,19924 | 0,28477 | 0,07623 | 0,81718 | 0,11282 |
| RPTOR | rs9900506 | 78622376 | normSNP | 0,1569 | A/G | 60,8 | 0,121466 | 0,03274 | 0,08182 | 0,01306 | 0,82757 | 0,01398 |
| RPTOR | rs9914825 | 78622612 | eSNP | 0,3922 | T/C | 64,8 | 0,034284 | 0,01773 | 0,65754 | 0,02605 | 0,08417 | 0,01773 |
| RPTOR | - | 78623515 | normSNP | 0 | A/C | 77,8 | 0,756052 | 0,36131 | 0,66142 | 0,15362 | 0,82058 | 0,37356 |
| RPTOR | rs7212142 | 78623941 | ecreSNP | 0,3266 | G/A | 58,5 | 0,030329 | 0,1116 | 0,47784 | 0,03774 | 0,51329 | 0,11186 |
| RPTOR | rs9894736 | 78624722 | eSNP | 0,3609 | G/A | 58,5 | 0,030329 | 0,1116 | 0,47784 | 0,03774 | 0,51329 | 0,11186 |
| RPTOR | rs11421875 | 78625335 | normSNP | 0,502 | A/C | 71,6 | 0,429777 | 0,02909 | 0,52216 | 0,05544 | 0,08586 | 0,02909 |
| RPTOR | rs62069682 | 78625419 | normSNP | 0,2139 | G/A | 87,5 | 0,615347 | 0,12352 | 0,30762 | 0,49425 | 0,11004 | 0,12352 |
| RPTOR | rs11871066 | 78625984 | eSNP | 0,7413 | A/G | 68,8 | 0,001035 | 0,58909 | 0,82757 | 0,49425 | 0,82902 | 0,58909 |
| RPTOR | rs9911795 | 78626115 | normSNP | 0,114 | C/T | 77,3 | 0,368313 | 0,20547 | 0,07879 | 0,39341 | 0,16844 | 0,08286 |
| RPTOR | rs12601247 | 78626132 | eSNP | 0,5607 | G/A | 65,3 | 0,155603 | 0,01592 | 1 | 0,01207 | 0,13384 | 0,01592 |
| RPTOR | rs9912051 | 78626258 | eSNP | 0,4983 | T/C | 65,3 | 0,155603 | 0,01592 | 1 | 0,01207 | 0,13384 | 0,01592 |
| RPTOR | rs4889873 | 78627063 | normSNP | 0 | A | 100 | - | - | - | - | - | - |
| RPTOR | rs62069686 | 78627423 | eSNP | 0,7486 | G/A | 65,3 | 0,155603 | 0,01592 | 1 | 0,01207 | 0,13384 | 0,01592 |
| RPTOR | rs7220294 | 78627753 | eSNP | 0,0744 | T/C | 71,6 | 0,792807 | 0,03585 | 0,01003 | 0,39341 | 0,03058 | 0,01433 |
| RPTOR | - | 78627839 | normSNP | 0 | A/C | 81,8 | 1 | 0,26682 | - | - | - | - |
| RPTOR | rs35675302 | 78627976 | eSNP | 0,5293 | T/C | 58,5 | 0,030329 | 0,1116 | 0,47784 | 0,03774 | 0,51329 | 0,11186 |
| RPTOR | rs6565471 | 78628256 | normSNP | 0 | T/C | 98,9 | 0 | 1 | - | - | - | - |
| RPTOR | rs12939557 | 78628432 | normSNP | 0 | G/A | 85,2 | 0,387915 | 0,27904 | 0,22367 | 0,55323 | 0,12463 | 0,41595 |
| RPTOR | rs7210730 | 78629025 | normSNP | 0,1728 | A/G | 69,3 | 0,000024 | 0,50782 | 0,27966 | 0,7871 | 0,30762 | 0,41732 |
| RPTOR | - | 78629270 | normSNP | 0 | A/C | 75,6 | 0,382987 | 0,2138 | 0,08182 | 0,69315 | 0,11107 | 0,1312 |
| RPTOR | rs74969730 | 78629271 | normSNP | 0,1204 | A/C | 75,6 | 0,382987 | 0,2138 | 0,08182 | 0,69315 | 0,11107 | 0,1312 |
| RPTOR | - | 78629273 | normSNP | 0 | A/C | 63,1 | 0,000001 | 0,2099 | 0,08686 | 0,46622 | 0,19307 | 0,16451 |
| RPTOR | rs11650627 | 78629981 | eSNP | 0,7713 | C/T | 65,9 | 0,641308 | 0,00441 | 0,5169 | 0,00248 | 0,19965 | 0,00441 |
| RPTOR | - | 78633102 | normSNP | 0 | A/C | 58,5 | 0,667331 | 0,77054 | 0,82058 | 0,55939 | 0,52216 | 0,87505 |
| RPTOR | rs9319607 | 78633460 | normSNP | 0 | C/G | 93,2 | 0,151491 | 1 | - | - | - | - |
| RPTOR | rs9903851 | 78633497 | normSNP | 0,1118 | C/T | 69,3 | 0 | 0,85122 | 0,82757 | 0,79553 | 0,58001 | 1 |
| RPTOR | rs9897453 | 78634300 | normSNP | 0,1114 | A/G | 76,7 | 0,548234 | 0,14073 | 0,04897 | 0,39341 | 0,11107 | 0,05634 |
| RPTOR | rs12939076 | 78634810 | eSNP | 0,0658 | C/A | 71,6 | 0,792807 | 0,03585 | 0,01003 | 0,39341 | 0,03058 | 0,01433 |
| RPTOR | rs12947653 | 78634823 | eSNP | 0,0658 | T/A | 71,6 | 0,792807 | 0,03585 | 0,01003 | 0,39341 | 0,03058 | 0,01433 |
| RPTOR | rs57678654 | 78635988 | normSNP | 0,423 | C/T | 58,5 | 0,389541 | 0,64076 | 1 | 0,36558 | 0,5213 | 0,62832 |
| RPTOR | rs542170625 | 78636342 | normSNP | 0 | A/C | 89,2 | 0,251782 | 0,6234 | 0,7871 | 0,49425 | 0,77674 | 0,6234 |
| RPTOR | rs12601423 | 78636795 | eSNP | 0,0755 | G/A | 71,6 | 0,429777 | 0,01974 | 0,00522 | 0,64411 | 0,00971 | 0,01153 |
| RPTOR | - | 78637200 | normSNP | 0 | A/C | 86,9 | 0,03454 | 0,1282 | 0,06712 | 1 | 0,04367 | 0,15678 |
| RPTOR | rs12453880 | 78637655 | ecreSNP | 0,0682 | A/G | 71 | 0,607314 | 0,02007 | 0,00522 | 0,39341 | 0,0176 | 0,00842 |
| RPTOR | rs12936509 | 78638305 | normSNP | 0 | A/G | 85,2 | 0,387915 | 0,27904 | 0,22367 | 0,55323 | 0,12463 | 0,41595 |
| RPTOR | - | 78638327 | normSNP | 0 | A/C | 69,3 | 0,322109 | 0,03356 | 1 | 0,02605 | 0,19965 | 0,03356 |
| RPTOR | rs150560230 | 78639132 | creSNP | 0 | C/T | 61,9 | 0,01292 | 0,4747 | 0,64704 | 0,23023 | 0,82757 | 0,36025 |
| RPTOR | rs140836694 | 78639133 | normSNP | 0 | C/T | 64,8 | 0,101146 | 0,44902 | 0,50866 | 0,23023 | 1 | 0,29001 |
| RPTOR | - | 78639303 | normSNP | 0 | A/C | 68,8 | 1 | 0,97529 | 0,83078 | 1 | 0,83008 | 0,86875 |
| RPTOR | rs11653272 | 78639760 | eSNP | 0,5652 | T/G | 65,3 | 0,155603 | 0,01592 | 1 | 0,01207 | 0,13384 | 0,01592 |
| RPTOR | rs4889782 | 78640510 | normSNP | 0,1339 | T/C | 64,2 | 0,358877 | 0,12537 | 0,66142 | 0,07105 | 0,13466 | 0,61638 |
| RPTOR | rs4889874 | 78640548 | eSNP | 0 | A/G | 77,8 | 1 | 0,79426 | 0,51329 | 1 | 0,50293 | 0,58178 |
| RPTOR | rs1485330 | 78640822 | eSNP | 0,0675 | G/A | 71 | 0,607314 | 0,02007 | 0,00522 | 0,39341 | 0,0176 | 0,00842 |
| RPTOR | rs11150737 | 78641290 | normSNP | 0,4775 | A/G | 71 | 1 | 0,78749 | 0,66976 | 0,69315 | 0,5169 | 0,8666 |
| RPTOR | rs7218924 | 78641361 | normSNP | 0,1829 | C/T | 57,4 | 0,827507 | 0,12895 | 0,64704 | 0,04367 | 0,28578 | 0,15858 |
| RPTOR | - | 78641917 | normSNP | 0 | A/C | 72,7 | 1 | 0,01064 | 0,00258 | 0,39341 | 0,00876 | 0,00507 |
| RPTOR | rs1564864 | 78642045 | eSNP | 0,0676 | A/G | 71 | 0,607314 | 0,02007 | 0,00522 | 0,39341 | 0,0176 | 0,00842 |
| RPTOR | rs6565472 | 78642620 | eSNP | 0,7548 | C/T | 77,3 | 0,543266 | 0,33398 | 0,82902 | 0,24138 | 0,66142 | 0,33398 |
| RPTOR | rs537857567 | 78642786 | normSNP | 0 | A/C | 71,6 | 0,429777 | 0,01974 | 0,00522 | 0,64411 | 0,00971 | 0,01153 |
| RPTOR | rs6565473 | 78643206 | normSNP | 0,2958 | T/C | 57,4 | 0,030148 | 0,06987 | 0,1881 | 0,02262 | 0,50866 | 0,03814 |
| RPTOR | rs1485329 | 78643561 | eSNP | 0,1215 | A/G | 64,2 | 0,064645 | 0,06362 | 0,02574 | 0,23023 | 0,13218 | 0,01978 |
| RPTOR | rs117736066 | 78643859 | normSNP | 0 | A/G | 87,5 | 1 | 0,62227 | - | - | - | - |
| RPTOR | rs7221823 | 78645538 | normSNP | 0,7291 | T/A | 71 | 1 | 0,78749 | 0,66976 | 0,69315 | 0,5169 | 0,8666 |
| RPTOR | rs7217223 | 78645663 | normSNP | 0,7265 | C/T | 71 | 1 | 0,78749 | 0,66976 | 0,69315 | 0,5169 | 0,8666 |
| RPTOR | rs7217702 | 78645929 | normSNP | 0,2774 | G/C | 57,4 | 0,030148 | 0,06987 | 0,1881 | 0,02262 | 0,50866 | 0,03814 |
| RPTOR | - | 78646165 | normSNP | 0 | A/C | 84,7 | 0,450327 | 0,48769 | - | - | - | - |
| RPTOR | rs12603933 | 78646606 | eSNP | 0,3184 | A/G | 57,4 | 0,030148 | 0,06987 | 0,1881 | 0,02262 | 0,50866 | 0,03814 |
| RPTOR | rs9807041 | 78647870 | eSNP | 0,9584 | A/G | 77,3 | 0,543266 | 0,29234 | 0,5169 | 0,24138 | 1 | 0,29234 |
| RPTOR | rs11150738 | 78648127 | eSNP | 0,0641 | A/G | 75 | 1 | 0,05851 | 0,0176 | 0,64411 | 0,02768 | 0,03191 |
| RPTOR | rs4255830 | 78649230 | normSNP | 0,3 | A/G | 58,5 | 0,046655 | 0,11759 | 0,2755 | 0,03961 | 0,50866 | 0,0711 |
| RPTOR | rs368922754 | 78649836 | normSNP | 0,3956 | T/C | 88,6 | 1 | 0,30008 | 0,19307 | 1 | 0,28883 | 0,30008 |
| RPTOR | rs9319609 | 78650699 | eSNP | 0,5477 | G/A | 76,1 | 0,769863 | 0,56935 | 0,66683 | 0,29556 | 1 | 0,46428 |
| RPTOR | rs10468604 | 78650768 | normSNP | 0,3649 | C/T | 63,1 | 0,251473 | 0,27705 | 0,37493 | 0,28706 | 0,13384 | 0,86574 |
| RPTOR | rs10468603 | 78650932 | normSNP | 0,3732 | T/C | 64,2 | 0,167526 | 0,3147 | 0,27021 | 0,45614 | 0,13384 | 0,60615 |
| RPTOR | rs4889875 | 78651500 | eSNP | 0,1454 | C/T | 64,2 | 1 | 0,11244 | 0,08182 | 0,10141 | 0,5213 | 0,03782 |
| RPTOR | rs113071613 | 78651587 | eSNP | 0,1425 | A/C | 54,5 | 0,001094 | 0,01486 | 0,00807 | 0,0183 | 0,64704 | 0,00382 |
| RPTOR | rs9901333 | 78651781 | eSNP | 0,9393 | A/G | 77,3 | 0,543266 | 0,29234 | 0,5169 | 0,24138 | 1 | 0,29234 |
| RPTOR | rs7208283 | 78653003 | creSNP | 0 | G | 100 | - | - | - | - | - | - |
| RPTOR | rs9319610 | 78653614 | eSNP | 0,4408 | A/C | 51,1 | 0,205154 | 0,03082 | 1 | 0,01544 | 0,03058 | 0,15571 |
| RPTOR | rs2019154 | 78654344 | eSNP | 0,0677 | G/A | 72,7 | 0,590502 | 0,06141 | 0,01836 | 0,64411 | 0,03058 | 0,03156 |
| RPTOR | rs9897426 | 78654867 | eSNP | 0,0892 | A/T | 86,9 | 1 | 0,46622 | - | - | - | - |
| RPTOR | rs8075839 | 78654905 | eSNP | 0,16 | G/A | 64,8 | 1 | 0,15027 | 0,12969 | 0,10141 | 0,66847 | 0,05688 |
| RPTOR | rs34863264 | 78655312 | eSNP | 0,0676 | C/T | 71,6 | 0,429777 | 0,01974 | 0,00522 | 0,64411 | 0,00971 | 0,01153 |
| RPTOR | rs374797335 | 78655452 | normSNP | 0 | C/G | 71,6 | 0,12371 | 0,42964 | 0,19823 | 0,50044 | 0,36782 | 0,21315 |
| RPTOR | rs9901744 | 78655478 | normSNP | 0,2737 | C/G | 84,1 | 0,522684 | 0,24091 | - | - | - | - |
| RPTOR | rs9319611 | 78655613 | eSNP | 0,1368 | A/T | 64,8 | 1 | 0,15027 | 0,12969 | 0,10141 | 0,66847 | 0,05688 |
| RPTOR | rs57796737 | 78655917 | normSNP | 0,097 | C/T | 58 | 0 | 0,81928 | 0,7871 | 0,55323 | 1 | 0,63493 |
| RPTOR | rs7210015 | 78656487 | eSNP | 0,4243 | C/A | 51,7 | 0,139282 | 0,02178 | 0,81718 | 0,01544 | 0,01685 | 0,20593 |
| RPTOR | rs7214361 | 78657849 | eSNP | 0,1433 | A/G | 64,2 | 1 | 0,11244 | 0,08182 | 0,10141 | 0,5213 | 0,03782 |
| RPTOR | - | 78658204 | normSNP | 0 | A/C | 58 | 0 | 0,13009 | 0,13466 | 0,04345 | 0,5336 | 0,06138 |
| RPTOR | rs7209576 | 78658447 | eSNP | 0,4411 | G/A | 56,2 | 0,000467 | 0,052 | 0,0293 | 0,81312 | 0,03613 | 0,15308 |
| RPTOR | rs7211217 | 78658741 | eSNP | 0,7627 | C/T | 77,3 | 0,543266 | 0,29234 | 0,5169 | 0,24138 | 1 | 0,29234 |
| RPTOR | rs62067874 | 78659425 | normSNP | 0 | A/T | 90,3 | 0,002441 | 0,93957 | 0,76375 | 1 | 0,72474 | 0,83002 |
| RPTOR | rs8073610 | 78659427 | normSNP | 0 | A/T | 84,7 | 0,004149 | 0,52241 | 0,80249 | 0,39341 | 0,39373 | 0,85679 |
| RPTOR | rs557666597 | 78659450 | normSNP | 0,5435 | T/A | 75,6 | 0 | 0,46954 | 0,24675 | 0,26682 | 0,74706 | 0,21886 |
| RPTOR | rs7217011 | 78659925 | eSNP | 0,428 | A/C | 51,1 | 0,205154 | 0,03082 | 1 | 0,01544 | 0,03058 | 0,15571 |
| RPTOR | rs2873061 | 78660042 | eSNP | 0,1629 | G/A | 72,7 | 0,064133 | 0,10144 | 0,19585 | 0,03774 | 1 | 0,06128 |
| RPTOR | rs12942946 | 78660175 | eSNP | 0 | C/T | 84,7 | 0,414695 | 0,19381 | 0,1493 | 0,55323 | 0,07775 | 0,31252 |
| RPTOR | rs7216337 | 78660226 | normSNP | 0 | A/G | 98,9 | 0,016949 | 1 | - | - | - | - |
| RPTOR | - | 78660782 | normSNP | 0 | A/C | 69,9 | 0,004288 | 0,04757 | 0,0176 | 0,55939 | 0,0319 | 0,06157 |
| RPTOR | rs9901355 | 78661301 | eSNP | 0,9574 | G/A | 76,7 | 0,772392 | 0,53436 | 0,5169 | 0,29556 | 0,82571 | 0,35947 |
| RPTOR | rs11652856 | 78661640 | eSNP | 0,4254 | C/G | 51,7 | 0,139282 | 0,02178 | 0,81718 | 0,01544 | 0,01685 | 0,20593 |
| RPTOR | rs756075 | 78662909 | ecreSNP | 0,4199 | T/C | 51,1 | 0,205154 | 0,03082 | 1 | 0,01544 | 0,03058 | 0,15571 |
| RPTOR | rs34388940 | 78663350 | ecreSNP | 0,0679 | G/T | 70,5 | 0,454607 | 0,03538 | 0,00995 | 0,39341 | 0,0315 | 0,01389 |
| RPTOR | rs2316058 | 78663775 | eSNP | 0,1643 | C/G | 64,8 | 1 | 0,15027 | 0,12969 | 0,10141 | 0,66847 | 0,05688 |
| RPTOR | rs140193761 | 78664142 | normSNP | 0 | A/C | 56,2 | 0,66556 | 0,47767 | 0,25593 | 1 | 0,28477 | 0,45871 |
| RPTOR | rs4889882 | 78664217 | eSNP | 0,1392 | G/A | 65,3 | 1 | 0,04316 | 0,05101 | 0,03774 | 0,5213 | 0,01462 |
| RPTOR | rs7225028 | 78664613 | eSNP | 0,0962 | A/G | 86,9 | 1 | 0,46622 | - | - | - | - |
| RPTOR | rs12452916 | 78664655 | eSNP | 0,0674 | C/T | 71 | 0,607314 | 0,02007 | 0,00522 | 0,39341 | 0,0176 | 0,00842 |
| RPTOR | rs9907193 | 78665199 | eSNP | 0,0997 | A/T | 86,9 | 1 | 0,46622 | - | - | - | - |
| RPTOR | rs9906827 | 78665405 | eSNP | 0,4472 | T/C | 51,7 | 0,139282 | 0,02178 | 0,81718 | 0,01544 | 0,01685 | 0,20593 |
| RPTOR | - | 78666078 | normSNP | 0 | A/C | 70,5 | 0 | 0,52057 | 0,65278 | 0,32371 | 0,45614 | 0,45723 |
| RPTOR | rs7210372 | 78666191 | eSNP | 0,4284 | A/G | 51,7 | 0,139282 | 0,02178 | 0,81718 | 0,01544 | 0,01685 | 0,20593 |
| RPTOR | rs12943155 | 78667125 | eSNP | 0,0674 | A/G | 71 | 0,607314 | 0,02007 | 0,00522 | 0,39341 | 0,0176 | 0,00842 |
| RPTOR | rs6565476 | 78667460 | eSNP | 0,9573 | C/T | 77,3 | 0,543266 | 0,29234 | 0,5169 | 0,24138 | 1 | 0,29234 |
| RPTOR | rs4889784 | 78667863 | normSNP | 0,1193 | T/G | 70,5 | 1 | 0,46353 | 0,52216 | 0,23023 | 1 | 0,31125 |
| RPTOR | rs4889785 | 78667935 | eSNP | 0,0961 | T/C | 86,9 | 1 | 0,46622 | - | - | - | - |
| RPTOR | rs9889315 | 78668235 | eSNP | 0,0961 | A/G | 86,9 | 1 | 0,46622 | - | - | - | - |
| RPTOR | rs60469059 | 78668313 | eSNP | 0,096 | C/T | 86,9 | 1 | 0,46622 | - | - | - | - |
| RPTOR | rs11869453 | 78668430 | eSNP | 0,9829 | G/A | 77,3 | 0,543266 | 0,29234 | 0,5169 | 0,24138 | 1 | 0,29234 |
| RPTOR | rs869190 | 78668593 | eSNP | 0,0676 | G/T | 71,6 | 0,429777 | 0,01974 | 0,00522 | 0,64411 | 0,00971 | 0,01153 |
| RPTOR | rs884204 | 78669248 | eSNP | 0,1577 | C/T | 64,8 | 1 | 0,15027 | 0,12969 | 0,10141 | 0,66847 | 0,05688 |
| RPTOR | rs11655435 | 78669443 | eSNP | 0,9786 | G/C | 77,3 | 0,543266 | 0,29234 | 0,5169 | 0,24138 | 1 | 0,29234 |
| RPTOR | rs34177980 | 78669788 | ecreSNP | 0,0963 | C/T | 86,9 | 1 | 0,46622 | - | - | - | - |
| RPTOR | rs62067879 | 78669867 | ecreSNP | 0,9829 | T/A | 77,3 | 0,543266 | 0,29234 | 0,5169 | 0,24138 | 1 | 0,29234 |
| RPTOR | rs74002832 | 78670044 | ecreSNP | 0,0974 | T/C | 86,9 | 1 | 0,46622 | - | - | - | - |
| RPTOR | rs7208502 | 78670328 | ecreSNP | 0,4457 | T/C | 51,1 | 0,205154 | 0,03082 | 1 | 0,01544 | 0,03058 | 0,15571 |
| RPTOR | rs1471791 | 78670371 | ecreSNP | 0,0975 | G/A | 86,9 | 1 | 0,46622 | - | - | - | - |
| RPTOR | rs12945216 | 78670642 | eSNP | 0,0675 | C/T | 71,6 | 0,429777 | 0,01974 | 0,00522 | 0,64411 | 0,00971 | 0,01153 |
| RPTOR | rs9911171 | 78670903 | eSNP | 0,9829 | C/T | 77,3 | 0,543266 | 0,29234 | 0,5169 | 0,24138 | 1 | 0,29234 |
| RPTOR | rs148570652 | 78670971 | normSNP | 0 | A/C | 86,4 | 0,683252 | 0,01544 | - | - | - | - |
| RPTOR | rs35007677 | 78671067 | eSNP | 0,0961 | G/T | 86,9 | 1 | 0,46622 | - | - | - | - |
| RPTOR | rs68144719 | 78671515 | eSNP | 0,1392 | C/T | 64,2 | 1 | 0,11244 | 0,08182 | 0,10141 | 0,5213 | 0,03782 |
| RPTOR | - | 78671728 | normSNP | 0 | A/C | 52,8 | 0,019941 | 0,06381 | 0,07072 | 0,0319 | 0,82571 | 0,02048 |
| RPTOR | rs56930167 | 78671900 | normSNP | 0,221 | A/C | 54 | 0,002383 | 0,08117 | 0,04645 | 0,06001 | 0,82058 | 0,02509 |
| RPTOR | rs34290925 | 78671969 | eSNP | 0,0962 | G/A | 86,9 | 1 | 0,46622 | - | - | - | - |
| RPTOR | rs1471789 | 78672086 | eSNP | 0,1589 | G/A | 64,8 | 1 | 0,15027 | 0,12969 | 0,10141 | 0,66847 | 0,05688 |
| RPTOR | rs12948054 | 78672790 | normSNP | 0 | C/T | 85,2 | 0,387915 | 0,27904 | 0,22367 | 0,55323 | 0,12463 | 0,41595 |
| RPTOR | rs12940029 | 78673080 | eSNP | 0,1658 | C/T | 64,8 | 1 | 0,15027 | 0,12969 | 0,10141 | 0,66847 | 0,05688 |
| RPTOR | - | 78674170 | normSNP | 0 | A/C | 88,1 | 1 | 0,45249 | - | - | - | - |
| RPTOR | rs62067884 | 78674235 | normSNP | 0 | T/G | 84,7 | 1 | 0,30359 | 0,23605 | 0,49425 | 0,46622 | 0,30359 |
| RPTOR | rs79341363 | 78674236 | normSNP | 0 | A/G | 85,2 | 1 | 0,20288 | 0,1493 | 0,49425 | 0,32371 | 0,20288 |
| RPTOR | rs62067889 | 78674702 | eSNP | 0,0937 | C/G | 86,9 | 1 | 0,46622 | - | - | - | - |
| RPTOR | rs7211095 | 78675059 | eSNP | 0,4464 | T/G | 51,1 | 0,205154 | 0,03082 | 1 | 0,01544 | 0,03058 | 0,15571 |
| RPTOR | rs35232546 | 78675805 | eSNP | 0,0945 | T/C | 86,9 | 1 | 0,46622 | - | - | - | - |
| RPTOR | rs9894952 | 78676510 | eSNP | 0,0957 | T/C | 86,9 | 1 | 0,46622 | - | - | - | - |
| RPTOR | rs9894978 | 78676547 | eSNP | 0,1599 | C/T | 64,2 | 1 | 0,11244 | 0,08182 | 0,10141 | 0,5213 | 0,03782 |
| RPTOR | rs9895186 | 78676606 | eSNP | 0,0958 | T/C | 86,9 | 1 | 0,46622 | - | - | - | - |
| RPTOR | rs4062178 | 78676905 | eSNP | 0,0957 | T/C | 86,9 | 1 | 0,46622 | - | - | - | - |
| RPTOR | rs2315925 | 78677190 | eSNP | 0,0957 | T/A | 86,9 | 1 | 0,46622 | - | - | - | - |
| RPTOR | rs11867785 | 78677666 | eSNP | 0,1712 | A/C | 64,2 | 1 | 0,11244 | 0,08182 | 0,10141 | 0,5213 | 0,03782 |
| RPTOR | rs2315924 | 78678137 | eSNP | 0,097 | C/A | 86,9 | 1 | 0,46622 | - | - | - | - |
| RPTOR | rs8065598 | 78678243 | eSNP | 0,9861 | G/A | 77,3 | 0,543266 | 0,29234 | 0,5169 | 0,24138 | 1 | 0,29234 |
| RPTOR | rs9898952 | 78678399 | eSNP | 0,9861 | A/G | 77,3 | 0,543266 | 0,29234 | 0,5169 | 0,24138 | 1 | 0,29234 |
| RPTOR | rs12948040 | 78678434 | eSNP | 0,0661 | A/G | 71 | 0,607314 | 0,02007 | 0,00522 | 0,39341 | 0,0176 | 0,00842 |
| RPTOR | rs11150739 | 78678979 | eSNP | 0,9626 | A/G | 77,3 | 0,543266 | 0,29234 | 0,5169 | 0,24138 | 1 | 0,29234 |
| RPTOR | rs12939413 | 78679449 | eSNP | 0,0676 | C/T | 71 | 0,607314 | 0,02007 | 0,00522 | 0,39341 | 0,0176 | 0,00842 |
| RPTOR | rs12601001 | 78681084 | eSNP | 0,0676 | T/C | 71 | 0,607314 | 0,02007 | 0,00522 | 0,39341 | 0,0176 | 0,00842 |
| RPTOR | rs4889887 | 78681092 | normSNP | 0,1198 | T/C | 70,5 | 0,80348 | 0,7422 | 0,66976 | 0,45614 | 1 | 0,51194 |
| RPTOR | rs9900445 | 78681520 | eSNP | 0,0972 | A/G | 86,9 | 1 | 0,46622 | - | - | - | - |
| RPTOR | rs2306690 | 78681590 | eSNP | 0,1583 | G/A | 64,2 | 1 | 0,11244 | 0,08182 | 0,10141 | 0,5213 | 0,03782 |
| RPTOR | rs61391154 | 78682414 | eSNP | 0,1396 | G/T | 64,8 | 0,642017 | 0,0233 | 0,08417 | 0,00983 | 1 | 0,01274 |
| RPTOR | rs8081444 | 78682569 | eSNP | 0,9839 | G/A | 77,3 | 0,543266 | 0,29234 | 0,5169 | 0,24138 | 1 | 0,29234 |
| RPTOR | rs12450876 | 78682630 | eSNP | 0,0676 | A/G | 71 | 0,607314 | 0,02007 | 0,00522 | 0,39341 | 0,0176 | 0,00842 |
| RPTOR | rs12453034 | 78682756 | eSNP | 0,0679 | G/A | 71,6 | 0,792807 | 0,03585 | 0,01003 | 0,39341 | 0,03058 | 0,01433 |
| RPTOR | rs8068064 | 78683460 | normSNP | 0,2479 | A/G | 58 | 1 | 0,1193 | 0,82058 | 0,04367 | 0,20012 | 0,21439 |
| RPTOR | rs8068594 | 78683803 | eSNP | 0,9744 | G/T | 77,3 | 0,543266 | 0,29234 | 0,5169 | 0,24138 | 1 | 0,29234 |
| RPTOR | - | 78683918 | normSNP | 0 | A/C | 65,3 | 1 | 0,04316 | 0,05101 | 0,03774 | 0,5213 | 0,01462 |
| RPTOR | rs370411238 | 78684371 | normSNP | 0 | A/C | 59,7 | 0,000019 | 0,96663 | 0,83078 | 1 | 0,80828 | 0,89871 |
| RPTOR | - | 78685175 | normSNP | 0 | A/C | 80,1 | 1 | 0,16984 | 0,65754 | 0,24138 | 0,25593 | 0,16984 |
| RPTOR | rs67161723 | 78685176 | normSNP | 0,8673 | C/T | 51,7 | 0,005774 | 0,19173 | 0,07072 | 0,48769 | 0,26372 | 0,14349 |
| RPTOR | rs4889888 | 78685317 | normSNP | 0 | A/G | 98,9 | 0,016949 | 1 | - | - | - | - |
| RPTOR | rs12951739 | 78685626 | eSNP | 0,4483 | G/T | 63,1 | 0 | 0,35474 | 0,39146 | 0,81312 | 0,15304 | 0,71391 |
| RPTOR | rs34479366 | 78686090 | eSNP | 0,0896 | T/C | 86,9 | 1 | 0,46622 | - | - | - | - |
| RPTOR | rs12946217 | 78686566 | eSNP | 0,0676 | C/A | 71 | 0,607314 | 0,02007 | 0,00522 | 0,39341 | 0,0176 | 0,00842 |
| RPTOR | rs117092745 | 78686749 | normSNP | 0 | C/T | 88,1 | 1 | 0,80249 | - | - | - | - |
| RPTOR | rs9904867 | 78686890 | eSNP | 0,1001 | T/C | 86,9 | 1 | 0,46622 | - | - | - | - |
| RPTOR | rs12946206 | 78687270 | eSNP | 0,0676 | G/T | 71,6 | 0,429777 | 0,01974 | 0,00522 | 0,64411 | 0,00971 | 0,01153 |
| RPTOR | rs8071397 | 78688017 | eSNP | 0,4119 | G/A | 71 | 0,119262 | 0,39313 | 0,28477 | 0,29556 | 0,52216 | 0,19544 |
| RPTOR | rs6565477 | 78688770 | normSNP | 0 | G/A | 98,9 | 0 | 1 | - | - | - | - |
| RPTOR | rs7211818 | 78688903 | eSNP | 1 | A/G | 77,3 | 0,543266 | 0,29234 | 0,5169 | 0,24138 | 1 | 0,29234 |
| RPTOR | rs7216437 | 78689128 | eSNP | 0,1818 | C/T | 64,2 | 1 | 0,11244 | 0,08182 | 0,10141 | 0,5213 | 0,03782 |
| RPTOR | rs56246352 | 78689513 | normSNP | 0 | A/C | 64,2 | 0 | 1 | 1 | 0,8234 | 1 | 1 |
| RPTOR | rs12601066 | 78691256 | eSNP | 0,0676 | G/T | 71 | 0,607314 | 0,02007 | 0,00522 | 0,39341 | 0,0176 | 0,00842 |
| RPTOR | rs71368076 | 78691258 | eSNP | 0,0676 | A/T | 71 | 0,607314 | 0,02007 | 0,00522 | 0,39341 | 0,0176 | 0,00842 |
| RPTOR | rs12601200 | 78691263 | eSNP | 0,0674 | C/T | 71 | 0,607314 | 0,02007 | 0,00522 | 0,39341 | 0,0176 | 0,00842 |
| RPTOR | rs12944407 | 78691979 | eSNP | 0,0676 | C/T | 71 | 0,607314 | 0,02007 | 0,00522 | 0,39341 | 0,0176 | 0,00842 |
| RPTOR | rs6565478 | 78692004 | eSNP | 0,1804 | G/A | 64,2 | 1 | 0,11244 | 0,08182 | 0,10141 | 0,5213 | 0,03782 |
| RPTOR | rs4889889 | 78692063 | normSNP | 0 | A | 100 | - | - | - | - | - | - |
| RPTOR | rs9897608 | 78693282 | eSNP | 0,1566 | C/T | 64,8 | 1 | 0,15027 | 0,12969 | 0,10141 | 0,66847 | 0,05688 |
| RPTOR | rs9896820 | 78693534 | eSNP | 0,9754 | C/T | 76,7 | 0,381822 | 0,33657 | 0,66683 | 0,24138 | 0,82757 | 0,33657 |
| RPTOR | rs66463810 | 78694496 | eSNP | 0,1394 | G/A | 64,8 | 0,815986 | 0,05662 | 0,08182 | 0,03774 | 0,66944 | 0,02219 |
| RPTOR | rs7503911 | 78694997 | creSNP | 0,2621 | T/C | 56,8 | 0,664749 | 0,12131 | 0,81718 | 0,04367 | 0,19965 | 0,20647 |
| RPTOR | rs729996 | 78695215 | ecreSNP | 0,1412 | A/G | 64,8 | 1 | 0,15027 | 0,12969 | 0,10141 | 0,66847 | 0,05688 |
| RPTOR | rs9913162 | 78695546 | ecreSNP | 0,1827 | G/A | 64,8 | 1 | 0,15027 | 0,12969 | 0,10141 | 0,66847 | 0,05688 |
| RPTOR | rs140367792 | 78695664 | normSNP | 0,7508 | A/C | 81,2 | 0,286985 | 0,82485 | 0,65754 | 1 | 0,8234 | 0,82485 |
| RPTOR | rs8069141 | 78696273 | normSNP | 0 | G | 100 | - | - | - | - | - | - |
| RPTOR | rs61107058 | 78696294 | eSNP | 0,7625 | G/A | 77,3 | 0,543266 | 0,29234 | 0,5169 | 0,24138 | 1 | 0,29234 |
| RPTOR | rs74002861 | 78696861 | normSNP | 0,4031 | A/C | 88,1 | 1 | 0,446 | 0,30762 | 1 | 0,43624 | 0,446 |
| RPTOR | rs55825314 | 78696887 | normSNP | 0 | G/A | 86,4 | 0,683252 | 0,01544 | - | - | - | - |
| RPTOR | rs140995574 | 78697010 | normSNP | 0,2555 | A/C | 60,8 | 0,014695 | 0,03048 | 0,0093 | 0,43624 | 0,04345 | 0,03776 |
| RPTOR | rs4889890 | 78697422 | normSNP | 0 | T/G | 98,9 | 0 | 1 | - | - | - | - |
| RPTOR | rs8072229 | 78699819 | eSNP | 0,0619 | C/T | 71 | 0,607314 | 0,02007 | 0,00522 | 0,39341 | 0,0176 | 0,00842 |
| RPTOR | rs8072355 | 78699863 | eSNP | 0,0683 | C/T | 71 | 0,607314 | 0,02007 | 0,00522 | 0,39341 | 0,0176 | 0,00842 |
| RPTOR | rs8072836 | 78700151 | eSNP | 0,0618 | C/T | 71 | 0,607314 | 0,02007 | 0,00522 | 0,39341 | 0,0176 | 0,00842 |
| RPTOR | rs9899843 | 78700833 | eSNP | 0,7876 | C/T | 78,4 | 1 | 0,57104 | 0,66142 | 0,29556 | 1 | 0,46147 |
| RPTOR | rs11871154 | 78700939 | eSNP | 0,7886 | T/C | 79 | 0,752823 | 0,33071 | 0,66142 | 0,24138 | 0,8234 | 0,33071 |
| RPTOR | rs58418838 | 78700955 | eSNP | 0,0513 | C/T | 64,2 | 1 | 0,03624 | 0,08182 | 0,01951 | 0,83078 | 0,01623 |
| RPTOR | rs12941958 | 78701430 | eSNP | 0,0619 | T/A | 71 | 0,607314 | 0,02007 | 0,00522 | 0,39341 | 0,0176 | 0,00842 |
| RPTOR | rs8069295 | 78702123 | eSNP | 0,6687 | T/C | 68,2 | 0,46289 | 0,01512 | 0,83008 | 0,01207 | 0,19965 | 0,01512 |
| RPTOR | rs12951944 | 78702382 | eSNP | 0,0619 | T/G | 71 | 0,607314 | 0,02007 | 0,00522 | 0,39341 | 0,0176 | 0,00842 |
| RPTOR | rs9902376 | 78703855 | normSNP | 0 | C | 100 | - | - | - | - | - | - |
| RPTOR | rs9896771 | 78703899 | normSNP | 0 | G/A | 73,3 | 0,411688 | 0,18386 | 0,13218 | 0,12995 | 0,50293 | 0,07167 |
| RPTOR | rs57816483 | 78704156 | eSNP | 0,6687 | T/C | 68,2 | 0,46289 | 0,01512 | 0,83008 | 0,01207 | 0,19965 | 0,01512 |
| RPTOR | rs12946115 | 78704240 | eSNP | 0,0618 | A/C | 71 | 0,607314 | 0,02007 | 0,00522 | 0,39341 | 0,0176 | 0,00842 |
| RPTOR | rs17848706 | 78704618 | normSNP | 0 | G/T | 80,7 | 0,297649 | 0,34797 | 0,82058 | 0,15362 | 0,63199 | 0,47074 |
| RPTOR | rs12603074 | 78705292 | eSNP | 0,062 | G/A | 71 | 0,607314 | 0,02007 | 0,00522 | 0,39341 | 0,0176 | 0,00842 |
| RPTOR | rs2315921 | 78706517 | ecreSNP | 0,643 | A/T | 68,2 | 0,46289 | 0,01512 | 0,83008 | 0,01207 | 0,19965 | 0,01512 |
| RPTOR | rs117635805 | 78707141 | normSNP | 0 | A/T | 88,1 | 1 | 0,80249 | - | - | - | - |
| RPTOR | rs9915378 | 78708418 | eSNP | 0,0625 | G/A | 63,6 | 0,821795 | 0,08077 | 0,08182 | 0,05731 | 0,66847 | 0,02809 |
| RPTOR | rs9891160 | 78708633 | eSNP | 0,7889 | G/A | 79 | 0,752823 | 0,33071 | 0,66142 | 0,24138 | 0,8234 | 0,33071 |
| RPTOR | rs7503394 | 78709446 | normSNP | 0 | T | 100 | - | - | - | - | - | - |
| RPTOR | rs12601101 | 78709557 | eSNP | 0,0621 | A/G | 71,6 | 0,429777 | 0,01974 | 0,00522 | 0,64411 | 0,00971 | 0,01153 |
| RPTOR | rs35078731 | 78709996 | eSNP | 0,0621 | A/C | 73,3 | 0,594031 | 0,03529 | 0,00995 | 0,64411 | 0,01685 | 0,01976 |
| RPTOR | rs12952097 | 78710207 | eSNP | 0,0604 | C/A | 72,2 | 0,794069 | 0,02015 | 0,00522 | 0,39341 | 0,01685 | 0,00867 |
| RPTOR | rs12942288 | 78711247 | ecreSNP | 0,0596 | G/C | 72,7 | 0,590502 | 0,01958 | 0,00522 | 0,64411 | 0,0093 | 0,0118 |
| RPTOR | rs12952578 | 78711630 | ecreSNP | 0 | C/T | 75,6 | 1 | 0,03259 | 0,0093 | 0,64411 | 0,01469 | 0,0198 |
| RPTOR | rs12936454 | 78711723 | ecreSNP | 0,0598 | A/T | 72,2 | 0,794069 | 0,02015 | 0,00522 | 0,39341 | 0,01685 | 0,00867 |
| RPTOR | rs12951431 | 78711935 | ecreSNP | 0,0596 | G/A | 72,7 | 0,590502 | 0,01958 | 0,00522 | 0,64411 | 0,0093 | 0,0118 |
| RPTOR | rs3843963 | 78711966 | creSNP | 0,1619 | G/A | 56,2 | 1 | 0,15758 | 0,64704 | 0,0557 | 0,28578 | 0,17146 |
| RPTOR | rs9915755 | 78712071 | eSNP | 0,7845 | G/A | 79 | 0,752823 | 0,33071 | 0,66142 | 0,24138 | 0,8234 | 0,33071 |
| RPTOR | rs9890672 | 78712157 | eSNP | 0,771 | A/G | 77,8 | 1 | 0,15922 | 0,82757 | 0,11643 | 0,50293 | 0,15922 |
| RPTOR | rs71163995 | 78712330 | creSNP | 0 | A/C | 68,8 | 0,000044 | 0,20108 | 0,08417 | 0,17437 | 0,45249 | 0,07678 |
| RPTOR | rs6565479 | 78712423 | creSNP | 0,1631 | G/A | 56,2 | 1 | 0,15758 | 0,64704 | 0,0557 | 0,28578 | 0,17146 |
| RPTOR | rs117934333 | 78712660 | normSNP | 0 | A/G | 88,1 | 1 | 0,80249 | - | - | - | - |
| RPTOR | - | 78712763 | normSNP | 0 | A/C | 68,2 | 0,003209 | 0,058 | 0,0181 | 0,15304 | 0,15952 | 0,02272 |
| RPTOR | rs12943681 | 78712885 | eSNP | 0,0599 | G/A | 72,7 | 1 | 0,03577 | 0,00995 | 0,39341 | 0,0293 | 0,01463 |
| RPTOR | rs377147269 | 78712955 | normSNP | 0 | A/C | 66,5 | 0,000023 | 0,91226 | 0,66847 | 0,79553 | 0,80249 | 0,69215 |
| RPTOR | rs531050067 | 78712987 | normSNP | 0,1565 | A/C | 56,8 | 0,00106 | 0,6855 | 0,38541 | 0,63199 | 0,64704 | 0,43181 |
| RPTOR | rs62067925 | 78712990 | normSNP | 0,1575 | A/C | 64,8 | 0 | 0,69126 | 0,39146 | 0,62227 | 0,59681 | 0,4468 |
| RPTOR | rs3850125 | 78713204 | normSNP | 0,1631 | G/A | 56,2 | 1 | 0,15758 | 0,64704 | 0,0557 | 0,28578 | 0,17146 |
| RPTOR | rs111960383 | 78713238 | normSNP | 0,1639 | A/C | 58,5 | 0,046655 | 0,05453 | 0,01588 | 0,30762 | 0,12215 | 0,03695 |
| RPTOR | rs12947053 | 78714031 | normSNP | 0 | T/C | 84,7 | 0,414695 | 0,19381 | 0,1493 | 0,55323 | 0,07775 | 0,31252 |
| RPTOR | rs7406799 | 78714161 | normSNP | 0 | A | 100 | - | - | - | - | - | - |
| RPTOR | rs12947784 | 78714793 | eSNP | 0,0619 | C/T | 71 | 0,607314 | 0,02007 | 0,00522 | 0,39341 | 0,0176 | 0,00842 |
| RPTOR | rs8067547 | 78714842 | eSNP | 0,0602 | C/T | 72,2 | 0,794069 | 0,02015 | 0,00522 | 0,39341 | 0,01685 | 0,00867 |
| RPTOR | rs4889786 | 78715567 | eSNP | 0,0524 | C/T | 63,1 | 1 | 0,1016 | 0,12636 | 0,05731 | 0,83078 | 0,04055 |
| RPTOR | rs9674559 | 78715608 | eSNP | 0,7864 | A/G | 79 | 0,752823 | 0,33071 | 0,66142 | 0,24138 | 0,8234 | 0,33071 |
| RPTOR | rs2316057 | 78715680 | normSNP | 0,1624 | G/A | 56,2 | 1 | 0,15758 | 0,64704 | 0,0557 | 0,28578 | 0,17146 |
| RPTOR | rs2873058 | 78715697 | normSNP | 0,1614 | G/A | 56,2 | 1 | 0,15758 | 0,64704 | 0,0557 | 0,28578 | 0,17146 |
| RPTOR | rs12938982 | 78716122 | eSNP | 0,0619 | G/A | 71,6 | 0,429777 | 0,01974 | 0,00522 | 0,64411 | 0,00971 | 0,01153 |
| RPTOR | rs12942358 | 78716183 | normSNP | 0,1624 | G/A | 55,1 | 0,831558 | 0,15877 | 0,64017 | 0,0557 | 0,28578 | 0,16369 |
| RPTOR | - | 78716248 | normSNP | 0 | A/C | 58,5 | 0,084069 | 0,60585 | 0,64017 | 0,33085 | 0,82902 | 0,39344 |
| RPTOR | - | 78716375 | normSNP | 0 | A/C | 79 | 0,752823 | 0,33071 | 0,66142 | 0,24138 | 0,8234 | 0,33071 |
| RPTOR | rs12943019 | 78716417 | normSNP | 0,0593 | A/G | 72,2 | 1 | 0,09656 | 0,03206 | 0,69315 | 0,04897 | 0,06351 |
| RPTOR | rs79240811 | 78716418 | normSNP | 0,776 | T/C | 84,7 | 0,414695 | 0,24943 | 1 | 0,24138 | 0,45249 | 0,24943 |
| RPTOR | rs34285166 | 78716425 | normSNP | 0,3696 | G/A | 79 | 1 | 0,57719 | 0,82571 | 0,29556 | 0,82058 | 0,58001 |
| RPTOR | rs12950676 | 78716460 | normSNP | 0,1582 | T/C | 54 | 0,084562 | 0,07428 | 0,02351 | 0,46622 | 0,12636 | 0,07114 |
| RPTOR | rs35755748 | 78716461 | normSNP | 0,7695 | G/A | 89,2 | 0,054774 | 0,10745 | 0,58001 | 0,24138 | 0,12878 | 0,10745 |
| RPTOR | - | 78716490 | normSNP | 0 | A/C | 65,9 | 0,000008 | 0,41526 | 0,19823 | 0,61069 | 0,30762 | 0,29712 |
| RPTOR | rs12947503 | 78716600 | normSNP | 0,162 | A/G | 54 | 1 | 0,18476 | 0,64017 | 0,06712 | 0,28578 | 0,17551 |
| RPTOR | rs12951048 | 78716680 | normSNP | 0,1624 | G/A | 54,5 | 1 | 0,09741 | 0,64017 | 0,03199 | 0,20012 | 0,12674 |
| RPTOR | rs12941240 | 78716804 | normSNP | 0,1605 | C/T | 55,7 | 1 | 0,15977 | 0,48769 | 0,0557 | 0,39344 | 0,12492 |
| RPTOR | rs12950333 | 78716850 | normSNP | 0,1885 | T/C | 55,7 | 1 | 0,15977 | 0,48769 | 0,0557 | 0,39344 | 0,12492 |
| RPTOR | rs3764371 | 78717404 | eSNP | 0,2029 | C/A | 56,8 | 0,12954 | 0,03321 | 0,18267 | 0,0096 | 0,38541 | 0,02293 |
| RPTOR | rs56248419 | 78717501 | normSNP | 0 | C/T | 74,4 | 0,785467 | 0,00961 | 0,00249 | 0,64411 | 0,00426 | 0,00696 |
| RPTOR | rs56399116 | 78717505 | normSNP | 0 | T/G | 71,6 | 1 | 0,01804 | 0,00522 | 0,69315 | 0,00876 | 0,01742 |
| RPTOR | rs12950348 | 78717511 | normSNP | 0 | T/A | 73,3 | 0,78468 | 0,03261 | 0,00971 | 0,23023 | 0,04645 | 0,01098 |
| RPTOR | rs12950350 | 78717513 | normSNP | 0 | T/C | 73,9 | 0,584344 | 0,05393 | 0,0176 | 0,23023 | 0,07509 | 0,0179 |
| RPTOR | rs12941644 | 78717519 | normSNP | 0 | C/T | 79 | 0,518935 | 0,02463 | 0,00728 | 0,64411 | 0,01015 | 0,0184 |
| RPTOR | rs62067931 | 78717523 | normSNP | 0 | G/T | 78,4 | 0,53643 | 0,01346 | 0,00383 | 0,64411 | 0,00543 | 0,01115 |
| RPTOR | rs62067957 | 78717528 | normSNP | 0 | C/G | 83,5 | 0,236986 | 0,00341 | 0,00174 | 1 | 0,00082 | 0,01088 |
| RPTOR | rs528918725 | 78717565 | normSNP | 0 | T/A | 86,9 | 0 | 0,34898 | 0,36558 | 0,17368 | 0,55323 | 0,24609 |
| RPTOR | rs539712541 | 78717567 | normSNP | 0 | T/C | 87,5 | 0 | 0,20049 | 0,5336 | 0,17368 | 0,49425 | 0,20049 |
| RPTOR | rs12939914 | 78717582 | normSNP | 0,1658 | G/C | 71,6 | 0,000006 | 0,78925 | 1 | 0,58001 | 0,59681 | 0,78466 |
| RPTOR | rs139657129 | 78717901 | normSNP | 0 | T/G | 78,4 | 0 | 0,32685 | 0,1377 | 0,26682 | 0,39341 | 0,16957 |
| RPTOR | rs113949530 | 78717924 | normSNP | 0 | C/G | 68,8 | 0 | 0,66355 | 0,66142 | 0,80249 | 0,36558 | 0,89944 |
| RPTOR | rs74466951 | 78718675 | normSNP | 0,0861 | G/T | 84,7 | 0 | 0,71432 | 0,41692 | 0,50044 | 0,69315 | 0,42022 |
| RPTOR | rs71368083 | 78718707 | normSNP | 0 | C/T | 89,2 | 0,00689 | 0,94898 | 0,77674 | 1 | 0,74706 | 0,83441 |
| RPTOR | rs34891233 | 78718734 | normSNP | 0 | G/C | 72,2 | 0,794069 | 0,02015 | 0,00522 | 0,39341 | 0,01685 | 0,00867 |
| RPTOR | rs72851570 | 78718774 | normSNP | 0 | C/T | 66,5 | 1 | 0,07639 | 0,13218 | 0,03774 | 0,83008 | 0,03668 |
| RPTOR | rs12935984 | 78719106 | eSNP | 0,2696 | T/C | 50,6 | 0,291175 | 0,13388 | 0,81312 | 0,05373 | 0,13218 | 0,19722 |
| RPTOR | rs74002883 | 78719153 | normSNP | 0,1253 | G/T | 86,4 | 1 | 0,80598 | 0,80828 | 1 | 0,62227 | 0,80598 |
| RPTOR | rs11868257 | 78719170 | eSNP | 0,2689 | C/G | 51,7 | 0,139282 | 0,12952 | 0,81718 | 0,05373 | 0,12969 | 0,20593 |
| RPTOR | rs57673311 | 78719180 | eSNP | 0,6451 | G/C | 76,1 | 0,769863 | 0,17099 | 0,66683 | 0,11643 | 0,66142 | 0,17099 |
| RPTOR | rs11868266 | 78719260 | eSNP | 0,6451 | G/C | 76,1 | 0,769863 | 0,17099 | 0,66683 | 0,11643 | 0,66142 | 0,17099 |
| RPTOR | - | 78719319 | normSNP | 0 | A/C | 52,8 | 0,057316 | 0,07501 | 0,04 | 1 | 0,04897 | 0,21401 |
| RPTOR | rs11868826 | 78719571 | eSNP | 0,6468 | G/C | 76,1 | 0,769863 | 0,17099 | 0,66683 | 0,11643 | 0,66142 | 0,17099 |
| RPTOR | rs12601596 | 78719690 | eSNP | 0 | G/A | 65,9 | 0,641308 | 0,02841 | 0,01685 | 0,07105 | 0,19965 | 0,00763 |
| RPTOR | rs112983841 | 78719981 | eSNP | 0 | G/T | 66,5 | 1 | 0,15246 | 0,05255 | 0,50044 | 0,13218 | 0,07788 |
| RPTOR | rs138537551 | 78720001 | normSNP | 0 | C/T | 89,8 | 1 | 0,28883 | - | - | - | - |
| RPTOR | - | 78720727 | normSNP | 0 | A/C | 71 | 0,000001 | 0,64159 | 0,38076 | 0,41692 | 0,7871 | 0,34628 |
| RPTOR | rs3884384 | 78720729 | eSNP | 0,2762 | A/C | 50,6 | 0,139061 | 0,0596 | 0,0183 | 0,47784 | 0,12969 | 0,06715 |
| RPTOR | rs7213696 | 78721171 | normSNP | 0,2393 | G/A | 58 | 0,517852 | 0,04444 | 0,26372 | 0,01306 | 0,39146 | 0,03697 |
| RPTOR | rs60513142 | 78721364 | eSNP | 0,6475 | G/A | 75,6 | 0,574804 | 0,16586 | 0,83008 | 0,11643 | 0,51329 | 0,16586 |
| RPTOR | rs12947909 | 78721708 | eSNP | 0,06 | A/G | 72,2 | 0,794069 | 0,02015 | 0,00522 | 0,39341 | 0,01685 | 0,00867 |
| RPTOR | rs376883753 | 78722277 | normSNP | 0 | A/G | 85,2 | 0 | 0,39119 | 0,17437 | 0,28706 | 0,45614 | 0,18039 |
| RPTOR | rs147576596 | 78722281 | normSNP | 0,111 | T/G | 82,4 | 0,000002 | 0,01594 | 0,00502 | 0,17368 | 0,01951 | 0,01517 |
| RPTOR | rs71368085 | 78722514 | normSNP | 0,0582 | T/C | 72,7 | 0,185157 | 0,05874 | 0,0176 | 0,28706 | 0,07072 | 0,0247 |
| RPTOR | rs2467398 | 78723235 | normSNP | 0 | T/G | 71,6 | 0 | 0,62875 | 0,34928 | 0,33749 | 1 | 0,33674 |
| RPTOR | rs3929295 | 78723447 | eSNP | 0,2756 | C/G | 52,8 | 0,139838 | 0,05423 | 1 | 0,02736 | 0,05101 | 0,20511 |
| RPTOR | rs4602089 | 78723985 | ecreSNP | 0,0587 | A/G | 72,2 | 0,794069 | 0,02015 | 0,00522 | 0,39341 | 0,01685 | 0,00867 |
| RPTOR | rs12939143 | 78724610 | eSNP | 0,2766 | A/G | 51,7 | 0,291447 | 0,08067 | 0,64017 | 0,02736 | 0,13218 | 0,11415 |
| RPTOR | rs12942526 | 78724677 | eSNP | 0,2762 | G/A | 51,7 | 0,291447 | 0,08067 | 0,64017 | 0,02736 | 0,13218 | 0,11415 |
| RPTOR | rs12600633 | 78725381 | ecreSNP | 0,0586 | G/A | 72,2 | 0,794069 | 0,02015 | 0,00522 | 0,39341 | 0,01685 | 0,00867 |
| RPTOR | rs12601089 | 78725470 | ecreSNP | 0,0585 | C/T | 72,2 | 0,794069 | 0,02015 | 0,00522 | 0,39341 | 0,01685 | 0,00867 |
| RPTOR | rs9902224 | 78725624 | ecreSNP | 0,2749 | G/A | 51,7 | 0,291447 | 0,08067 | 0,64017 | 0,02736 | 0,13218 | 0,11415 |
| RPTOR | rs9899782 | 78725640 | ecreSNP | 0,6484 | G/A | 75,6 | 0,574804 | 0,16586 | 0,83008 | 0,11643 | 0,51329 | 0,16586 |
| RPTOR | rs4396582 | 78725691 | ecreSNP | 0,2749 | G/A | 52,3 | 0,205576 | 0,06885 | 0,81718 | 0,02736 | 0,08417 | 0,15513 |
| RPTOR | rs2316056 | 78725710 | creSNP | 0,0982 | C/T | 59,7 | 0,660254 | 0,07402 | 0,17614 | 0,0314 | 0,83112 | 0,03738 |
| RPTOR | rs9902435 | 78725713 | ecreSNP | 0,2753 | G/A | 51,7 | 0,291447 | 0,08067 | 0,64017 | 0,02736 | 0,13218 | 0,11415 |
| RPTOR | rs9902639 | 78725766 | ecreSNP | 0,2766 | G/A | 51,7 | 0,291447 | 0,08067 | 0,64017 | 0,02736 | 0,13218 | 0,11415 |
| RPTOR | rs3887735 | 78726174 | ecreSNP | 0,2766 | T/G | 52,3 | 0,205576 | 0,06885 | 0,81718 | 0,02736 | 0,08417 | 0,15513 |
| RPTOR | rs4401092 | 78726194 | ecreSNP | 0,2766 | G/A | 51,7 | 0,291447 | 0,08067 | 0,64017 | 0,02736 | 0,13218 | 0,11415 |
| RPTOR | rs4969230 | 78726261 | creSNP | 0,099 | T/C | 58,5 | 0,667331 | 0,16989 | 0,25593 | 0,07623 | 0,83112 | 0,08177 |
| RPTOR | rs55792002 | 78726403 | ecreSNP | 0,6484 | G/C | 75,6 | 0,574804 | 0,16586 | 0,83008 | 0,11643 | 0,51329 | 0,16586 |
| RPTOR | rs3073626 | 78726780 | normSNP | 0,2766 | A/C | 76,7 | 0,004937 | 0,12195 | 0,04345 | 0,50044 | 0,07775 | 0,08657 |
| RPTOR | rs34178751 | 78727178 | eSNP | 0,2766 | A/C | 51,1 | 0,205154 | 0,10996 | 1 | 0,05373 | 0,08417 | 0,25666 |
| RPTOR | rs12953290 | 78727473 | eSNP | 0,0585 | C/T | 72,2 | 0,794069 | 0,02015 | 0,00522 | 0,39341 | 0,01685 | 0,00867 |
| RPTOR | rs12944923 | 78727734 | ecreSNP | 0,0591 | T/C | 71 | 0,607314 | 0,0054 | 0,00123 | 0,39341 | 0,00492 | 0,0027 |
| RPTOR | rs2291359 | 78728314 | eSNP | 0 | G/A | 65,3 | 1 | 0,0458 | 0,01685 | 0,17368 | 0,13384 | 0,01462 |
| RPTOR | rs2291360 | 78728521 | eSNP | 0,2446 | G/A | 68,8 | 0,318835 | 0,62693 | 0,51955 | 0,39341 | 0,83112 | 0,38056 |
| RPTOR | rs72851589 | 78728638 | normSNP | 0 | A/G | 87,5 | 1 | 0,01251 | - | - | - | - |
| RPTOR | rs11658179 | 78728694 | normSNP | 0,1034 | C/T | 59,1 | 0,827931 | 0,19374 | 0,36782 | 0,07623 | 0,66976 | 0,11693 |
| RPTOR | rs12944470 | 78728813 | eSNP | 0,0585 | G/A | 72,7 | 0,590502 | 0,01958 | 0,00522 | 0,64411 | 0,0093 | 0,0118 |
| RPTOR | - | 78728929 | normSNP | 0 | A/C | 72,7 | 0,590502 | 0,01958 | 0,00522 | 0,64411 | 0,0093 | 0,0118 |
| RPTOR | rs12938221 | 78729057 | eSNP | 0,0585 | T/C | 72,2 | 0,794069 | 0,02015 | 0,00522 | 0,39341 | 0,01685 | 0,00867 |
| RPTOR | rs12947166 | 78729668 | eSNP | 0,0585 | T/C | 72,2 | 0,794069 | 0,02015 | 0,00522 | 0,39341 | 0,01685 | 0,00867 |
| RPTOR | rs11385165 | 78729989 | normSNP | 0,0585 | A/C | 77,3 | 0,764065 | 0,0153 | 0,00426 | 0,64411 | 0,00639 | 0,01155 |
| RPTOR | rs12602935 | 78730985 | eSNP | 0 | A/G | 67 | 1 | 0,03034 | 0,0176 | 0,07105 | 0,19823 | 0,00821 |
| RPTOR | rs12947328 | 78730987 | eSNP | 0,0586 | G/T | 72,7 | 0,590502 | 0,01958 | 0,00522 | 0,64411 | 0,0093 | 0,0118 |
| RPTOR | rs56863354 | 78731015 | eSNP | 0,0586 | G/A | 72,7 | 0,590502 | 0,01958 | 0,00522 | 0,64411 | 0,0093 | 0,0118 |
| RPTOR | rs56032528 | 78731085 | eSNP | 0,0584 | T/G | 72,7 | 0,590502 | 0,01958 | 0,00522 | 0,64411 | 0,0093 | 0,0118 |
| RPTOR | rs72851595 | 78732071 | eSNP | 0,1706 | G/T | 82,4 | 1 | 0,40162 | 0,82058 | 0,49425 | 0,48769 | 0,40162 |
| RPTOR | rs4969235 | 78732673 | normSNP | 0,1019 | A/T | 59,7 | 1 | 0,2063 | 0,50293 | 0,07623 | 0,52216 | 0,16184 |
| RPTOR | rs7217606 | 78733219 | eSNP | 0,0583 | T/A | 71,6 | 1 | 0,01876 | 0,00522 | 0,23023 | 0,0293 | 0,00636 |
| RPTOR | rs9895584 | 78733492 | eSNP | 0,5343 | A/G | 75,6 | 0,574804 | 0,16586 | 0,83008 | 0,11643 | 0,51329 | 0,16586 |
| RPTOR | rs61197798 | 78733528 | eSNP | 0,4541 | G/A | 75,6 | 0,574804 | 0,16586 | 0,83008 | 0,11643 | 0,51329 | 0,16586 |
| RPTOR | rs7217825 | 78733901 | eSNP | 0,0545 | T/C | 93,2 | 0,330711 | 0,7387 | 0,74706 | 1 | 0,50044 | 0,7387 |
| RPTOR | rs78550371 | 78734086 | normSNP | 0 | G/A | 73,3 | 0,594031 | 0,43519 | 0,19965 | 0,64411 | 0,27966 | 0,21354 |
| RPTOR | rs117736345 | 78734100 | normSNP | 0 | A/C | 61,4 | 0,259162 | 0,83451 | 1 | 0,58001 | 0,66449 | 0,77153 |
| RPTOR | rs66607237 | 78734107 | normSNP | 0 | T/C | 82,4 | 0 | 0,92411 | 0,79553 | 1 | 0,69315 | 0,88027 |
| RPTOR | rs60064073 | 78734391 | normSNP | 0,1026 | C/T | 59,7 | 0,660254 | 0,07402 | 0,17614 | 0,0314 | 0,83112 | 0,03738 |
| RPTOR | rs12950541 | 78734493 | eSNP | 0,0583 | G/A | 72,2 | 0,794069 | 0,02015 | 0,00522 | 0,39341 | 0,01685 | 0,00867 |
| RPTOR | rs9902665 | 78734576 | creSNP | 0,1026 | C/T | 59,7 | 0,660254 | 0,07402 | 0,17614 | 0,0314 | 0,83112 | 0,03738 |
| RPTOR | rs8080789 | 78735202 | ecreSNP | 0,0515 | G/A | 93,2 | 0,330711 | 0,7387 | 0,74706 | 1 | 0,50044 | 0,7387 |
| RPTOR | rs55966178 | 78735892 | eSNP | 0,5333 | T/G | 75,6 | 0,574804 | 0,16586 | 0,83008 | 0,11643 | 0,51329 | 0,16586 |
| RPTOR | rs12450464 | 78736865 | eSNP | 0,6394 | G/A | 75,6 | 0,574804 | 0,16586 | 0,83008 | 0,11643 | 0,51329 | 0,16586 |
| RPTOR | rs7215564 | 78737287 | eSNP | 0 | T/C | 93,2 | 0,330711 | 0,7387 | 0,74706 | 1 | 0,50044 | 0,7387 |
| RPTOR | rs9913009 | 78737350 | eSNP | 0,5333 | T/C | 75,6 | 0,574804 | 0,16586 | 0,83008 | 0,11643 | 0,51329 | 0,16586 |
| RPTOR | - | 78737917 | normSNP | 0 | A/C | 55,1 | 0 | 0,66412 | 0,51955 | 0,36782 | 0,79553 | 0,3958 |
| RPTOR | - | 78737970 | normSNP | 0 | A/C | 83 | 0,263109 | 0,54103 | 1 | 0,29556 | 0,62227 | 0,70416 |
| RPTOR | rs12948369 | 78738554 | eSNP | 0,0581 | T/C | 87,5 | 0,002945 | 0,38424 | 0,17437 | 0,64411 | 0,21023 | 0,23983 |
| RPTOR | rs57860295 | 78738791 | eSNP | 0,6377 | G/A | 75,6 | 0,574804 | 0,16586 | 0,83008 | 0,11643 | 0,51329 | 0,16586 |
| RPTOR | rs10871488 | 78739079 | eSNP | 0 | C/T | 93,2 | 0,330711 | 0,7387 | 0,74706 | 1 | 0,50044 | 0,7387 |
| RPTOR | rs11869890 | 78739163 | eSNP | 0,5333 | G/T | 75,6 | 0,574804 | 0,16586 | 0,83008 | 0,11643 | 0,51329 | 0,16586 |
| RPTOR | rs9901049 | 78739340 | eSNP | 0,5333 | T/G | 75,6 | 0,574804 | 0,16586 | 0,83008 | 0,11643 | 0,51329 | 0,16586 |
| RPTOR | rs67271326 | 78739591 | eSNP | 0 | C/G | 65,9 | 1 | 0,07383 | 0,03058 | 0,17368 | 0,19823 | 0,02359 |
| RPTOR | rs66508321 | 78739672 | eSNP | 0 | G/A | 65,3 | 1 | 0,0458 | 0,01685 | 0,17368 | 0,13384 | 0,01462 |
| RPTOR | rs9900687 | 78739721 | eSNP | 0 | T/C | 93,2 | 0,330711 | 0,7387 | 0,74706 | 1 | 0,50044 | 0,7387 |
| RPTOR | rs4969246 | 78740579 | eSNP | 0 | A/G | 93,2 | 0,330711 | 0,7387 | 0,74706 | 1 | 0,50044 | 0,7387 |
| RPTOR | - | 78740635 | normSNP | 0 | A/C | 88,1 | 0,096441 | 0,30655 | 0,59681 | 0,24138 | 0,77674 | 0,30655 |
| RPTOR | rs9908929 | 78740913 | eSNP | 0,5309 | C/G | 75,6 | 0,574804 | 0,16586 | 0,83008 | 0,11643 | 0,51329 | 0,16586 |
| RPTOR | rs62069375 | 78741007 | normSNP | 0,1025 | T/C | 60,2 | 0,82439 | 0,08727 | 0,26372 | 0,0314 | 0,66976 | 0,0569 |
| RPTOR | rs12943552 | 78741031 | eSNP | 0,0594 | C/T | 72,2 | 0,794069 | 0,02015 | 0,00522 | 0,39341 | 0,01685 | 0,00867 |
| RPTOR | rs12953163 | 78741916 | eSNP | 0,0594 | C/T | 73,3 | 0,594031 | 0,01007 | 0,00258 | 0,64411 | 0,00463 | 0,00692 |
| RPTOR | rs9915518 | 78742076 | eSNP | 0,5336 | G/A | 75,6 | 0,574804 | 0,16586 | 0,83008 | 0,11643 | 0,51329 | 0,16586 |
| RPTOR | rs7213638 | 78742093 | eSNP | 0,5322 | C/T | 76,1 | 0,769863 | 0,17099 | 0,66683 | 0,11643 | 0,66142 | 0,17099 |
| RPTOR | rs59920425 | 78742260 | eSNP | 0,1694 | C/A | 82,4 | 1 | 0,40162 | 0,82058 | 0,49425 | 0,48769 | 0,40162 |
| RPTOR | rs11150741 | 78742362 | eSNP | 0,2447 | A/G | 68,8 | 0,318835 | 0,62693 | 0,51955 | 0,39341 | 0,83112 | 0,38056 |
| RPTOR | rs9890313 | 78742436 | eSNP | 0,5336 | C/T | 75,6 | 0,574804 | 0,16586 | 0,83008 | 0,11643 | 0,51329 | 0,16586 |
| RPTOR | rs9891139 | 78742454 | eSNP | 0,5274 | A/G | 75,6 | 0,574804 | 0,16586 | 0,83008 | 0,11643 | 0,51329 | 0,16586 |
| RPTOR | rs9894401 | 78742526 | eSNP | 0,5607 | G/A | 75,6 | 0,574804 | 0,16586 | 0,83008 | 0,11643 | 0,51329 | 0,16586 |
| RPTOR | rs9903212 | 78742732 | eSNP | 0,5326 | T/G | 75,6 | 0,574804 | 0,16586 | 0,83008 | 0,11643 | 0,51329 | 0,16586 |
| RPTOR | rs9897595 | 78742771 | eSNP | 0,5336 | A/G | 75,6 | 0,574804 | 0,16586 | 0,83008 | 0,11643 | 0,51329 | 0,16586 |
| RPTOR | rs9897830 | 78742879 | eSNP | 0,5329 | A/G | 75,6 | 0,574804 | 0,16586 | 0,83008 | 0,11643 | 0,51329 | 0,16586 |
| RPTOR | - | 78743031 | normSNP | 0 | A/C | 79,5 | 0,750596 | 0,16228 | 0,65754 | 0,11643 | 0,64704 | 0,16228 |
| RPTOR | rs9895847 | 78743114 | eSNP | 0,5343 | G/A | 76,1 | 0,769863 | 0,17099 | 0,66683 | 0,11643 | 0,66142 | 0,17099 |
| RPTOR | rs59144499 | 78743284 | eSNP | 0,5336 | A/G | 75,6 | 0,574804 | 0,16586 | 0,83008 | 0,11643 | 0,51329 | 0,16586 |
| RPTOR | rs9989484 | 78743611 | eSNP | 0,5336 | T/G | 75,6 | 0,574804 | 0,16586 | 0,83008 | 0,11643 | 0,51329 | 0,16586 |
| RPTOR | rs111912114 | 78743727 | normSNP | 0,5257 | A/C | 75,6 | 0,574804 | 0,16586 | 0,83008 | 0,11643 | 0,51329 | 0,16586 |
| RPTOR | rs4380096 | 78743748 | eSNP | 0,0503 | A/G | 66,5 | 0,812114 | 0,01833 | 0,0093 | 0,07105 | 0,13384 | 0,0047 |
| RPTOR | rs55851355 | 78744050 | eSNP | 0,5343 | A/G | 75,6 | 0,574804 | 0,16586 | 0,83008 | 0,11643 | 0,51329 | 0,16586 |
| RPTOR | rs55774251 | 78744073 | eSNP | 0,5336 | C/T | 75,6 | 0,574804 | 0,16586 | 0,83008 | 0,11643 | 0,51329 | 0,16586 |
| RPTOR | rs57396399 | 78744228 | eSNP | 0,1707 | G/A | 82,4 | 1 | 0,40162 | 0,82058 | 0,49425 | 0,48769 | 0,40162 |
| RPTOR | rs9903994 | 78744325 | eSNP | 0,5343 | G/C | 75,6 | 0,574804 | 0,16586 | 0,83008 | 0,11643 | 0,51329 | 0,16586 |
| RPTOR | rs2340773 | 78744395 | eSNP | 0 | C/T | 93,8 | 0,282904 | 1 | 1 | 1 | 0,72474 | 1 |
| RPTOR | rs74001037 | 78744480 | normSNP | 0,1232 | A/G | 85,8 | 1 | 0,62812 | 0,63199 | 1 | 0,46622 | 0,62812 |
| RPTOR | rs7208831 | 78744676 | eSNP | 0,5343 | G/T | 75,6 | 0,574804 | 0,16586 | 0,83008 | 0,11643 | 0,51329 | 0,16586 |
| RPTOR | rs66827493 | 78745215 | eSNP | 0 | C/T | 65,9 | 1 | 0,07383 | 0,03058 | 0,17368 | 0,19823 | 0,02359 |
| RPTOR | rs60956249 | 78745526 | eSNP | 0,535 | G/T | 75,6 | 0,574804 | 0,16586 | 0,83008 | 0,11643 | 0,51329 | 0,16586 |
| RPTOR | rs73366835 | 78745543 | eSNP | 0,535 | T/G | 75,6 | 0,574804 | 0,16586 | 0,83008 | 0,11643 | 0,51329 | 0,16586 |
| RPTOR | rs9989442 | 78745642 | eSNP | 0,5343 | C/T | 75,6 | 0,574804 | 0,16586 | 0,83008 | 0,11643 | 0,51329 | 0,16586 |
| RPTOR | rs4969256 | 78745643 | eSNP | 0 | G/A | 67 | 1 | 0,03034 | 0,0176 | 0,07105 | 0,19823 | 0,00821 |
| RPTOR | rs9989446 | 78745759 | eSNP | 0,5343 | A/G | 76,1 | 0,560648 | 0,17853 | 1 | 0,07799 | 0,36782 | 0,49188 |
| RPTOR | rs7216268 | 78746146 | eSNP | 0,5343 | C/A | 75,6 | 0,574804 | 0,16586 | 0,83008 | 0,11643 | 0,51329 | 0,16586 |
| RPTOR | rs7216295 | 78746186 | eSNP | 0,5343 | C/T | 75,6 | 0,574804 | 0,16586 | 0,83008 | 0,11643 | 0,51329 | 0,16586 |
| RPTOR | rs4969404 | 78746363 | normSNP | 0,0898 | C/T | 58 | 0,661265 | 0,19043 | 0,35932 | 0,07623 | 0,66944 | 0,11092 |
| RPTOR | rs12947507 | 78746684 | eSNP | 0,0596 | G/A | 71,6 | 0,792807 | 0,01074 | 0,0026 | 0,39341 | 0,0093 | 0,00494 |
| RPTOR | rs7221610 | 78746716 | eSNP | 0,5343 | A/G | 75,6 | 0,574804 | 0,16586 | 0,83008 | 0,11643 | 0,51329 | 0,16586 |
| RPTOR | - | 78746929 | normSNP | 0 | A/C | 76,7 | 0,772392 | 0,16286 | 0,82902 | 0,11643 | 0,50866 | 0,16286 |
| RPTOR | rs7221492 | 78746958 | eSNP | 0,5336 | C/A | 75,6 | 0,574804 | 0,16586 | 0,83008 | 0,11643 | 0,51329 | 0,16586 |
| RPTOR | rs7211443 | 78747434 | ecreSNP | 0,5322 | T/A | 75,6 | 0,574804 | 0,16586 | 0,83008 | 0,11643 | 0,51329 | 0,16586 |
| RPTOR | rs7211454 | 78747461 | ecreSNP | 0,5384 | T/G | 75 | 0,570228 | 0,17286 | 0,66847 | 0,11643 | 0,66449 | 0,17286 |
| RPTOR | rs12941973 | 78747722 | ecreSNP | 0,0658 | C/T | 72,7 | 0,590502 | 0,01958 | 0,00522 | 0,64411 | 0,0093 | 0,0118 |
| RPTOR | rs9900956 | 78747744 | ecreSNP | 0,5363 | T/C | 75,6 | 0,574804 | 0,16586 | 0,83008 | 0,11643 | 0,51329 | 0,16586 |
| RPTOR | rs4969411 | 78747982 | eSNP | 0 | C/T | 93,2 | 0,330711 | 0,7387 | 0,74706 | 1 | 0,50044 | 0,7387 |
| RPTOR | rs12452243 | 78748327 | ecreSNP | 0,6378 | A/G | 75,6 | 0,574804 | 0,16586 | 0,83008 | 0,11643 | 0,51329 | 0,16586 |
| RPTOR | rs8064506 | 78748473 | ecreSNP | 0,5391 | T/C | 75,6 | 0,574804 | 0,16586 | 0,83008 | 0,11643 | 0,51329 | 0,16586 |
| RPTOR | rs8077901 | 78748802 | eSNP | 0,5384 | G/T | 75,6 | 0,574804 | 0,16586 | 0,83008 | 0,11643 | 0,51329 | 0,16586 |
| RPTOR | rs7208823 | 78749619 | eSNP | 0,5426 | A/G | 75,6 | 0,574804 | 0,16586 | 0,83008 | 0,11643 | 0,51329 | 0,16586 |
| RPTOR | rs7208971 | 78749671 | eSNP | 0,5426 | A/T | 75 | 0,570228 | 0,13364 | 1 | 0,11643 | 0,38541 | 0,13364 |
| RPTOR | rs7208536 | 78749786 | eSNP | 0,5419 | C/T | 75,6 | 0,574804 | 0,16586 | 0,83008 | 0,11643 | 0,51329 | 0,16586 |
| RPTOR | rs11450408 | 78749845 | normSNP | 0 | A/C | 88,6 | 0,000943 | 0,73427 | 0,77674 | 0,64411 | 0,50044 | 1 |
| RPTOR | rs9910524 | 78750043 | eSNP | 0,5377 | C/G | 76,1 | 0,377719 | 0,33657 | 0,83008 | 0,24138 | 0,66449 | 0,33657 |
| RPTOR | rs9912037 | 78750348 | eSNP | 0,2536 | C/A | 67,6 | 0,633079 | 0,68361 | 0,51955 | 0,45614 | 0,83078 | 0,40446 |
| RPTOR | rs11150742 | 78751066 | eSNP | 0,537 | G/C | 75 | 1 | 0,08488 | 0,83008 | 0,05544 | 0,38076 | 0,08488 |
| RPTOR | rs9898483 | 78751455 | eSNP | 0,5356 | T/C | 75,6 | 0,574804 | 0,16586 | 0,83008 | 0,11643 | 0,51329 | 0,16586 |
| RPTOR | rs9898212 | 78751489 | eSNP | 0,5346 | A/C | 75,6 | 0,574804 | 0,16586 | 0,83008 | 0,11643 | 0,51329 | 0,16586 |
| RPTOR | rs9904216 | 78751565 | eSNP | 0,6387 | T/G | 75,6 | 0,574804 | 0,16586 | 0,83008 | 0,11643 | 0,51329 | 0,16586 |
| RPTOR | rs9898441 | 78751603 | eSNP | 0,5377 | A/C | 75,6 | 0,574804 | 0,16586 | 0,83008 | 0,11643 | 0,51329 | 0,16586 |
| RPTOR | rs2340772 | 78752017 | eSNP | 0 | A/C | 93,2 | 0,330711 | 0,7387 | 0,74706 | 1 | 0,50044 | 0,7387 |
| RPTOR | rs2340771 | 78752947 | eSNP | 0 | C/A | 93,8 | 0,282904 | 0,48394 | 0,50044 | 1 | 0,28706 | 0,48394 |
| RPTOR | rs9908112 | 78753455 | eSNP | 0 | G/A | 92 | 0,082844 | 0,41018 | 1 | 0,49425 | 0,50044 | 0,41018 |
| RPTOR | rs9911979 | 78753849 | ecreSNP | 0,6367 | G/A | 75,6 | 0,574804 | 0,16586 | 0,83008 | 0,11643 | 0,51329 | 0,16586 |
| RPTOR | rs9915667 | 78754340 | ecreSNP | 0,1909 | G/A | 59,1 | 0,276226 | 0,01003 | 0,00543 | 0,05731 | 0,19823 | 0,00256 |
| RPTOR | rs12149993 | 78754499 | ecreSNP | 0,2651 | C/G | 68,8 | 0,318835 | 0,62693 | 0,51955 | 0,39341 | 0,83112 | 0,38056 |
| RPTOR | rs72852757 | 78754579 | ecreSNP | 0,6339 | G/C | 75 | 0,570228 | 0,17286 | 0,66847 | 0,11643 | 0,66449 | 0,17286 |
| RPTOR | rs2340770 | 78754770 | ecreSNP | 0 | C/T | 92 | 0,082844 | 0,41018 | 1 | 0,49425 | 0,50044 | 0,41018 |
| RPTOR | rs12452137 | 78755149 | eSNP | 0,5336 | A/G | 75,6 | 0,574804 | 0,16586 | 0,83008 | 0,11643 | 0,51329 | 0,16586 |
| RPTOR | rs9893335 | 78755268 | eSNP | 0,5965 | G/A | 75,6 | 0,574804 | 0,16586 | 0,83008 | 0,11643 | 0,51329 | 0,16586 |
| RPTOR | rs2340769 | 78755271 | eSNP | 0 | T/C | 93,2 | 0,330711 | 0,7387 | 0,74706 | 1 | 0,50044 | 0,7387 |
| RPTOR | rs11150743 | 78755331 | eSNP | 0,5965 | G/C | 75,6 | 0,574804 | 0,16586 | 0,83008 | 0,11643 | 0,51329 | 0,16586 |
| RPTOR | rs6565480 | 78755639 | ecreSNP | 0,5965 | A/G | 75,6 | 0,574804 | 0,16586 | 0,83008 | 0,11643 | 0,51329 | 0,16586 |
| RPTOR | rs12949402 | 78755764 | ecreSNP | 0,0599 | G/A | 72,2 | 0,794069 | 0,02015 | 0,00522 | 0,39341 | 0,01685 | 0,00867 |
| RPTOR | rs12325875 | 78756492 | eSNP | 0,5965 | C/T | 75,6 | 0,574804 | 0,16586 | 0,83008 | 0,11643 | 0,51329 | 0,16586 |
| RPTOR | rs11150744 | 78756696 | eSNP | 0,0595 | A/C | 72,7 | 0,590502 | 0,01958 | 0,00522 | 0,64411 | 0,0093 | 0,0118 |
| RPTOR | rs11150745 | 78757626 | eSNP | 0,0802 | A/G | 65,3 | 1 | 0,00431 | 0,00105 | 0,17368 | 0,0181 | 0,00182 |
| RPTOR | rs67583258 | 78757949 | eSNP | 0,5813 | C/T | 75,6 | 0,574804 | 0,16586 | 0,83008 | 0,11643 | 0,51329 | 0,16586 |
| RPTOR | rs67414861 | 78757970 | eSNP | 0,5813 | C/T | 75,6 | 0,574804 | 0,16586 | 0,83008 | 0,11643 | 0,51329 | 0,16586 |
| RPTOR | rs8066075 | 78758449 | eSNP | 0,5805 | C/T | 80,7 | 0,080583 | 0,09852 | 0,64704 | 0,07799 | 0,1377 | 0,72707 |
| RPTOR | rs7209132 | 78758567 | eSNP | 0,2901 | T/C | 59,7 | 0,014365 | 0,78816 | 0,5169 | 0,61069 | 0,8234 | 0,49478 |
| RPTOR | rs8066994 | 78758702 | eSNP | 0,5813 | A/G | 75,6 | 0,144297 | 0,06185 | 0,51329 | 0,01888 | 0,48769 | 0,1419 |
| RPTOR | rs4969424 | 78759739 | eSNP | 0 | C/A | 89,8 | 0,004197 | 0,48452 | 1 | 0,29556 | 0,50044 | 0,67161 |
| RPTOR | rs4969425 | 78760902 | eSNP | 0,0686 | G/A | 65,9 | 1 | 0,07383 | 0,03058 | 0,17368 | 0,19823 | 0,02359 |
| RPTOR | rs11150746 | 78761389 | eSNP | 0,4127 | A/G | 69,3 | 1 | 0,66919 | 0,39295 | 1 | 0,38896 | 0,50961 |
| RPTOR | rs4969266 | 78761546 | normSNP | 0 | C/T | 76,7 | 0,548234 | 0,42403 | 0,51329 | 0,39341 | 0,25593 | 0,86362 |
| RPTOR | rs11150747 | 78761607 | normSNP | 0 | C/T | 78,4 | 1 | 0,55435 | 1 | 0,29556 | 0,65278 | 0,71302 |
| RPTOR | rs7225755 | 78761708 | normSNP | 0,0582 | A/C | 61,4 | 0,821753 | 0,39445 | 0,37493 | 0,21023 | 1 | 0,20126 |
| RPTOR | rs72852775 | 78761726 | normSNP | 0 | A/G | 85,8 | 0,367167 | 0,32998 | 0,1377 | 0,55323 | 0,19307 | 0,14827 |
| RPTOR | rs7224758 | 78761732 | eSNP | 0 | G/A | 85,8 | 0,679266 | 0,45226 | 0,22367 | 1 | 0,20941 | 0,28306 |
| RPTOR | rs12450690 | 78762136 | eSNP | 0,2428 | G/T | 75,6 | 1 | 0,89063 | 1 | 0,64411 | 0,82571 | 0,85965 |
| RPTOR | rs7225553 | 78762233 | normSNP | 0 | G/A | 87,5 | 0,123522 | 0,47688 | 0,43624 | 0,55323 | 0,26682 | 0,67299 |
| RPTOR | rs4969426 | 78762808 | normSNP | 0,1308 | T/A | 68,8 | 0,618894 | 0,7933 | 0,66847 | 0,69315 | 0,5213 | 0,86505 |
| RPTOR | rs4969427 | 78762886 | normSNP | 0 | G/A | 79 | 1 | 0,40402 | 0,27021 | 0,29556 | 0,49599 | 0,19488 |
| RPTOR | rs719781 | 78763580 | creSNP | 0 | G/A | 68,8 | 1 | 0,04659 | 0,0181 | 1 | 0,0176 | 0,06707 |
| RPTOR | rs4969429 | 78765219 | normSNP | 0,1338 | G/A | 77,8 | 0,220867 | 0,58909 | 0,82902 | 0,49425 | 0,82757 | 0,58909 |
| RPTOR | rs35292957 | 78765712 | creSNP | 0 | C/G | 68,2 | 1 | 0,09228 | 0,05358 | 0,72474 | 0,03058 | 0,19485 |
| RPTOR | rs8069962 | 78766464 | creSNP | 0,1057 | A/G | 62,5 | 0,364913 | 0,27865 | 0,65754 | 0,17368 | 0,19965 | 0,74055 |
| RPTOR | rs12953234 | 78766752 | normSNP | 0,0769 | A/G | 52,8 | 1 | 0,55775 | 0,33749 | 0,43624 | 0,83112 | 0,28307 |
| RPTOR | rs12601434 | 78767275 | normSNP | 0 | C/G | 69,9 | 0,800508 | 0,22995 | 0,08686 | 0,69315 | 0,13218 | 0,12663 |
| RPTOR | rs2672889 | 78768561 | normSNP | 0,1346 | C/T | 71,6 | 0,429777 | 0,30861 | 0,83112 | 0,15362 | 0,39146 | 0,72189 |
| RPTOR | rs2589138 | 78769600 | creSNP | 0 | C/T | 73,3 | 0,594031 | 0,13509 | 0,08686 | 0,15362 | 0,27966 | 0,04939 |
| RPTOR | rs77068358 | 78770616 | normSNP | 0,0631 | G/C | 89,2 | 1 | 0,79553 | - | - | - | - |
| RPTOR | rs908238 | 78770624 | eSNP | 0 | G/C | 83,5 | 0,449015 | 0,25232 | 0,16844 | 1 | 0,24675 | 0,25232 |
| RPTOR | rs9909563 | 78770960 | normSNP | 0,0529 | A/T | 72,7 | 0,279925 | 0,68222 | 0,39344 | 1 | 0,39146 | 0,46332 |
| RPTOR | rs11654508 | 78771947 | normSNP | 0 | A/G | 75 | 1 | 0,34798 | 0,51955 | 0,15362 | 1 | 0,28749 |
| RPTOR | rs9889802 | 78772281 | eSNP | 0 | C/G | 83,5 | 1 | 0,49236 | 0,24675 | 1 | 0,23605 | 0,30009 |
| RPTOR | rs9890864 | 78772393 | eSNP | 0 | A/G | 88,1 | 0,343361 | 0,96399 | 0,79553 | 1 | 0,7871 | 0,82297 |
| RPTOR | rs2589139 | 78772502 | normSNP | 0 | G/C | 64,2 | 1 | 0,01987 | 0,0293 | 0,33085 | 0,00512 | 0,26654 |
| RPTOR | rs9895861 | 78772519 | eSNP | 0 | C/G | 83 | 1 | 0,36821 | 0,16844 | 1 | 0,15952 | 0,21633 |
| RPTOR | rs58419250 | 78773313 | eSNP | 0 | G/A | 83 | 1 | 0,36821 | 0,16844 | 1 | 0,15952 | 0,21633 |
| RPTOR | rs7208789 | 78773522 | eSNP | 0 | C/T | 93,8 | 0,282904 | 1 | 1 | 1 | 0,72474 | 1 |
| RPTOR | rs7222621 | 78773652 | eSNP | 0 | G/A | 83 | 1 | 0,36821 | 0,16844 | 1 | 0,15952 | 0,21633 |
| RPTOR | rs7224354 | 78773665 | eSNP | 0 | A/T | 83 | 1 | 0,36821 | 0,16844 | 1 | 0,15952 | 0,21633 |
| RPTOR | rs7222643 | 78773681 | eSNP | 0 | G/A | 83 | 1 | 0,36821 | 0,16844 | 1 | 0,15952 | 0,21633 |
| RPTOR | rs11657137 | 78773720 | normSNP | 0 | A/G | 89,8 | 1 | 0,28883 | - | - | - | - |
| RPTOR | rs11652074 | 78773977 | normSNP | 0 | G/A | 89,8 | 1 | 0,28883 | - | - | - | - |
| RPTOR | rs9903560 | 78774203 | eSNP | 0 | G/A | 83 | 1 | 0,36821 | 0,16844 | 1 | 0,15952 | 0,21633 |
| RPTOR | rs72853708 | 78774305 | normSNP | 0 | G/A | 89,8 | 1 | 0,28883 | - | - | - | - |
| RPTOR | rs9905648 | 78774380 | eSNP | 0 | C/G | 83 | 1 | 0,36821 | 0,16844 | 1 | 0,15952 | 0,21633 |
| RPTOR | rs9891152 | 78774765 | eSNP | 0 | T/C | 83 | 1 | 0,36821 | 0,16844 | 1 | 0,15952 | 0,21633 |
| RPTOR | rs72853711 | 78775052 | normSNP | 0,0867 | T/C | 85,2 | 1 | 0,61217 | 0,33749 | 1 | 0,32371 | 0,39587 |
| RPTOR | rs7219896 | 78775571 | eSNP | 0 | T/C | 83 | 1 | 0,36821 | 0,16844 | 1 | 0,15952 | 0,21633 |
| RPTOR | rs11654579 | 78775619 | normSNP | 0 | G/T | 89,8 | 1 | 0,28883 | - | - | - | - |
| RPTOR | rs7215319 | 78775693 | eSNP | 0 | C/T | 85,8 | 0,367167 | 0,79729 | 0,62227 | 0,55323 | 0,79553 | 0,53872 |
| RPTOR | rs7214338 | 78775815 | eSNP | 0 | G/A | 82,4 | 1 | 0,50775 | 0,25593 | 1 | 0,24675 | 0,30676 |
| RPTOR | rs7214655 | 78775970 | eSNP | 0 | G/A | 83 | 1 | 0,36821 | 0,16844 | 1 | 0,15952 | 0,21633 |
| RPTOR | rs60087410 | 78776001 | eSNP | 0 | A/C | 83 | 1 | 0,36821 | 0,16844 | 1 | 0,15952 | 0,21633 |
| RPTOR | rs116234138 | 78776103 | normSNP | 0 | G/T | 89,8 | 1 | 0,28883 | - | - | - | - |
| RPTOR | rs59189552 | 78776108 | eSNP | 0 | C/T | 83 | 1 | 0,36821 | 0,16844 | 1 | 0,15952 | 0,21633 |
| RPTOR | rs9913285 | 78776506 | eSNP | 0 | C/G | 83 | 1 | 0,36821 | 0,16844 | 1 | 0,15952 | 0,21633 |
| RPTOR | rs9914359 | 78776597 | eSNP | 0 | A/G | 83 | 1 | 0,36821 | 0,16844 | 1 | 0,15952 | 0,21633 |
| RPTOR | rs7225574 | 78777941 | eSNP | 0 | G/T | 73,3 | 0,283292 | 0,54895 | 0,28578 | 1 | 0,28274 | 0,35959 |
| RPTOR | rs9893956 | 78778398 | eSNP | 0 | G/C | 83 | 1 | 0,36821 | 0,16844 | 1 | 0,15952 | 0,21633 |
| RPTOR | rs9895714 | 78778427 | normSNP | 0 | A/C | 55,1 | 1 | 0,08391 | 0,03613 | 1 | 0,05409 | 0,17372 |
| RPTOR | rs9908495 | 78778692 | eSNP | 0,0664 | T/C | 68,8 | 0,318835 | 0,13578 | 0,05255 | 1 | 0,05409 | 0,1131 |
| RPTOR | - | 78778940 | normSNP | 0 | A/C | 88,6 | 0,296329 | 0,53811 | 0,28883 | 1 | 0,26682 | 0,36223 |
| RPTOR | rs11651724 | 78779158 | normSNP | 0 | G/A | 89,8 | 1 | 0,28883 | - | - | - | - |
| RPTOR | rs60024815 | 78779664 | normSNP | 0 | A/T | 75 | 1 | 0,34798 | 0,51955 | 0,15362 | 1 | 0,28749 |
| RPTOR | rs59153096 | 78779665 | normSNP | 0 | A/C | 75 | 1 | 0,34798 | 0,51955 | 0,15362 | 1 | 0,28749 |
| RPTOR | rs4969443 | 78779805 | eSNP | 0 | A/G | 83 | 1 | 0,36821 | 0,16844 | 1 | 0,15952 | 0,21633 |
| RPTOR | rs4969277 | 78779981 | eSNP | 0 | C/T | 83 | 1 | 0,36821 | 0,16844 | 1 | 0,15952 | 0,21633 |
| RPTOR | rs4969444 | 78780010 | eSNP | 0 | G/A | 83 | 1 | 0,36821 | 0,16844 | 1 | 0,15952 | 0,21633 |
| RPTOR | rs4969445 | 78780080 | eSNP | 0 | G/T | 83 | 1 | 0,36821 | 0,16844 | 1 | 0,15952 | 0,21633 |
| RPTOR | rs7223529 | 78781356 | normSNP | 0 | T/A | 76,1 | 0,560648 | 0,20999 | 0,66449 | 0,07799 | 0,65278 | 0,30177 |
| RPTOR | rs2672886 | 78781651 | normSNP | 0 | C/T | 51,1 | 0,398701 | 0,68616 | 0,47784 | 0,46622 | 1 | 0,38563 |
| RPTOR | rs72853728 | 78781670 | normSNP | 0 | T/C | 84,1 | 0,686169 | 0,81558 | 0,81718 | 1 | 0,64017 | 0,81558 |
| RPTOR | rs2672887 | 78781804 | normSNP | 0 | T/A | 54 | 0,6672 | 0,0761 | 0,10415 | 0,03961 | 0,83078 | 0,02635 |
| RPTOR | rs734338 | 78782340 | normSNP | 0 | G/A | 55,1 | 0,831558 | 0,12279 | 0,06001 | 0,17437 | 0,52216 | 0,04333 |
| RPTOR | rs2090204 | 78782690 | normSNP | 0 | G/T | 86,9 | 0,633425 | 0,00261 | 0,00082 | 0,49425 | 0,00347 | 0,00261 |
| RPTOR | rs9904611 | 78783157 | normSNP | 0 | G/A | 81,2 | 0,171764 | 0,85576 | 0,64704 | 0,64411 | 0,80828 | 0,58736 |
| RPTOR | rs11658248 | 78783782 | normSNP | 0 | G/A | 83 | 0,707919 | 0,32205 | 0,24675 | 0,55323 | 0,1493 | 0,42973 |
| RPTOR | rs370281100 | 78783848 | normSNP | 0 | A/C | 61,9 | 0,02279 | 0,12307 | 0,28274 | 0,28883 | 0,04345 | 0,88969 |
| RPTOR | rs7211857 | 78784003 | normSNP | 0 | A/G | 85,2 | 1 | 0,60356 | 0,63199 | 0,49425 | 1 | 0,60356 |
| RPTOR | rs139834432 | 78784077 | normSNP | 0 | A/C | 89,2 | 1 | 0,7922 | 0,59681 | 1 | 0,7871 | 0,7922 |
| RPTOR | - | 78784200 | normSNP | 0 | A/C | 83,5 | 0,449015 | 0,64433 | 0,64704 | 1 | 0,48769 | 0,64433 |
| RPTOR | rs9915426 | 78784312 | normSNP | 0 | A/G | 80,7 | 1 | 0,58533 | 0,50293 | 0,55323 | 0,35932 | 0,69946 |
| RPTOR | - | 78784364 | normSNP | 0 | A/C | 83,5 | 0,449015 | 1 | 1 | 1 | 0,81718 | 1 |
| RPTOR | rs9899671 | 78784495 | normSNP | 0 | T/A | 80,7 | 1 | 0,58533 | 0,50293 | 0,55323 | 0,35932 | 0,69946 |
| RPTOR | rs72853735 | 78784728 | normSNP | 0 | A/G | 87,5 | 1 | 0,31725 | 0,20941 | 1 | 0,30762 | 0,31725 |
| RPTOR | rs2333990 | 78784925 | normSNP | 0 | G/A | 80,7 | 1 | 0,58533 | 0,50293 | 0,55323 | 0,35932 | 0,69946 |
| RPTOR | rs9894955 | 78784990 | normSNP | 0 | A/T | 87,5 | 1 | 0,31725 | 0,20941 | 1 | 0,30762 | 0,31725 |
| RPTOR | rs4998688 | 78785482 | normSNP | 0 | C/T | 81,8 | 0,178114 | 0,65754 | - | - | - | - |
| RPTOR | rs35688078 | 78785548 | normSNP | 0 | C/A | 88,6 | 1 | 0,60518 | 0,43624 | 1 | 0,59681 | 0,60518 |
| RPTOR | rs191278357 | 78785666 | normSNP | 0 | G/A | 89,2 | 1 | 0,79553 | - | - | - | - |
| RPTOR | rs4969455 | 78785746 | normSNP | 0 | C/T | 95,5 | 0,153145 | 1 | 0,69315 | 1 | 1 | 1 |
| RPTOR | rs2672903 | 78786389 | normSNP | 0 | G/T | 95,5 | 0,153145 | 1 | 0,69315 | 1 | 1 | 1 |
| RPTOR | rs72853745 | 78786586 | normSNP | 0 | C/T | 88,6 | 1 | 0,60518 | 0,43624 | 1 | 0,59681 | 0,60518 |
| RPTOR | rs8065459 | 78786989 | normSNP | 0 | C/T | 88,1 | 1 | 0,446 | 0,30762 | 1 | 0,43624 | 0,446 |
| RPTOR | rs2138126 | 78787230 | normSNP | 0 | A/G | 52,8 | 1 | 0,1533 | 0,05373 | 0,43624 | 0,28578 | 0,09054 |
| RPTOR | rs7212127 | 78787662 | normSNP | 0 | G/A | 65,9 | 0,812114 | 0,52832 | 0,83008 | 0,33085 | 0,38896 | 0,75499 |
| RPTOR | rs2138125 | 78788489 | normSNP | 0 | A/G | 52,3 | 1 | 0,14313 | 0,05373 | 0,30762 | 0,39344 | 0,06808 |
| RPTOR | rs2048753 | 78789288 | normSNP | 0 | G/A | 76,1 | 0,137642 | 0,09248 | 0,0315 | 1 | 0,03058 | 0,04794 |
| RPTOR | rs2589131 | 78789736 | eSNP | 0 | G/A | 82,4 | 0,130197 | 0,4357 | 0,34928 | 0,64411 | 0,20941 | 0,58395 |
| RPTOR | rs9898104 | 78789761 | normSNP | 0 | T/C | 80,1 | 1 | 0,69693 | 0,65754 | 0,55323 | 0,49599 | 0,8476 |
| RPTOR | rs9898554 | 78789966 | normSNP | 0 | T/G | 80,1 | 1 | 0,69693 | 0,65754 | 0,55323 | 0,49599 | 0,8476 |
| RPTOR | rs9893314 | 78790275 | normSNP | 0 | A/G | 80,1 | 1 | 0,69693 | 0,65754 | 0,55323 | 0,49599 | 0,8476 |
| RPTOR | rs2589132 | 78790487 | normSNP | 0 | C/T | 53,4 | 0,830084 | 0,13786 | 0,05373 | 0,28883 | 0,39295 | 0,06093 |
| RPTOR | rs11150748 | 78790586 | normSNP | 0 | G/C | 81,8 | 1 | 0,574 | 0,49599 | 0,55323 | 0,34928 | 0,69675 |
| RPTOR | rs9912928 | 78791502 | normSNP | 0 | T/G | 80,1 | 1 | 0,69693 | 0,65754 | 0,55323 | 0,49599 | 0,8476 |
| RPTOR | rs1567961 | 78791682 | normSNP | 0 | T/C | 58 | 0,829388 | 0,36711 | 0,65278 | 0,26682 | 0,19965 | 0,76239 |
| RPTOR | rs9907099 | 78791866 | normSNP | 0 | C/T | 80,7 | 1 | 0,58533 | 0,50293 | 0,55323 | 0,35932 | 0,69946 |
| RPTOR | rs28617259 | 78792701 | normSNP | 0 | G/A | 88,1 | 1 | 0,446 | 0,30762 | 1 | 0,43624 | 0,446 |
| RPTOR | rs9893680 | 78792758 | normSNP | 0 | T/G | 65,3 | 0,816855 | 0,45458 | 0,66683 | 0,33085 | 0,28274 | 0,87546 |
| RPTOR | rs2589133 | 78793476 | creSNP | 0 | A/G | 53,4 | 0,282756 | 0,21042 | 0,32371 | 0,09388 | 0,66683 | 0,10083 |
| RPTOR | rs72853757 | 78793497 | creSNP | 0 | T/C | 81,2 | 0,286985 | 0,26777 | 0,18267 | 1 | 0,26372 | 0,26777 |
| RPTOR | rs2589134 | 78793593 | creSNP | 0 | A/G | 88,1 | 1 | 0,00434 | 0,00171 | 1 | 0,00347 | 0,00434 |
| RPTOR | rs7221948 | 78793642 | normSNP | 0 | T/C | 60,2 | 0,82439 | 0,66306 | 0,8234 | 0,36558 | 0,66976 | 0,52839 |
| RPTOR | rs7217174 | 78793653 | normSNP | 0 | C/T | 80,7 | 1 | 0,58533 | 0,50293 | 0,55323 | 0,35932 | 0,69946 |
| RPTOR | rs9895510 | 78794032 | normSNP | 0 | C/G | 80,7 | 1 | 0,58533 | 0,50293 | 0,55323 | 0,35932 | 0,69946 |
| RPTOR | rs76334049 | 78794255 | normSNP | 0 | G/T | 85,8 | 0,681984 | 0,47784 | - | - | - | - |
| RPTOR | rs2138122 | 78794427 | normSNP | 0 | C/T | 82,4 | 0,290417 | 0,26071 | 0,17614 | 1 | 0,25593 | 0,26071 |
| RPTOR | rs11653207 | 78794778 | normSNP | 0 | T/G | 80,1 | 1 | 0,69693 | 0,65754 | 0,55323 | 0,49599 | 0,8476 |
| RPTOR | rs2589135 | 78795008 | normSNP | 0 | C/T | 51,7 | 0,199168 | 0,31386 | 0,30762 | 0,17437 | 0,82902 | 0,13645 |
| RPTOR | rs9910469 | 78795252 | normSNP | 0 | T/C | 80,1 | 1 | 0,69693 | 0,65754 | 0,55323 | 0,49599 | 0,8476 |
| RPTOR | rs9908195 | 78795555 | normSNP | 0 | G/A | 82,4 | 0,290417 | 0,26071 | 0,17614 | 1 | 0,25593 | 0,26071 |
| RPTOR | rs9911574 | 78795868 | normSNP | 0 | A/G | 60,2 | 0,82439 | 0,66306 | 0,8234 | 0,36558 | 0,66976 | 0,52839 |
| RPTOR | rs12103708 | 78796361 | normSNP | 0 | T/A | 80,1 | 1 | 0,69693 | 0,65754 | 0,55323 | 0,49599 | 0,8476 |
| RPTOR | rs10672537 | 78796595 | normSNP | 0 | A/C | 66,5 | 1 | 0,52822 | 0,28274 | 0,50044 | 0,51955 | 0,26376 |
| RPTOR | rs2672901 | 78796666 | normSNP | 0 | C/T | 73,3 | 0,78468 | 0,42906 | 0,39146 | 0,23023 | 0,82571 | 0,24109 |
| RPTOR | rs56273414 | 78796704 | normSNP | 0 | T/C | 81,2 | 0,725846 | 0,16003 | 0,11706 | 0,49425 | 0,25593 | 0,16003 |
| RPTOR | rs2019040 | 78797066 | normSNP | 0 | T/A | 88,1 | 1 | 0,00434 | 0,00171 | 1 | 0,00347 | 0,00434 |
| RPTOR | rs9895174 | 78797412 | normSNP | 0 | C/T | 80,7 | 1 | 0,58533 | 0,50293 | 0,55323 | 0,35932 | 0,69946 |
| RPTOR | rs8082099 | 78798488 | normSNP | 0 | G/A | 82,4 | 0,290417 | 0,26071 | 0,17614 | 1 | 0,25593 | 0,26071 |
| RPTOR | rs182273256 | 78798972 | normSNP | 0 | C/T | 82,4 | 0,290417 | 0,26071 | 0,17614 | 1 | 0,25593 | 0,26071 |
| RPTOR | rs34630038 | 78799047 | normSNP | 0 | G/A | 80,7 | 1 | 0,77868 | 0,8234 | 0,55323 | 0,64704 | 1 |
| RPTOR | rs114445964 | 78799101 | normSNP | 0 | C/T | 85,2 | 0,685494 | 0,15952 | - | - | - | - |
| RPTOR | - | 78799263 | normSNP | 0 | A/C | 83 | 0,707919 | 0,76988 | 0,81718 | 0,55323 | 0,63199 | 1 |
| RPTOR | rs35904260 | 78799310 | normSNP | 0 | A/G | 62,5 | 0,364913 | 0,23879 | 0,18267 | 0,17368 | 0,66944 | 0,09586 |
| RPTOR | rs7212390 | 78799793 | normSNP | 0 | G/A | 79 | 1 | 0,78875 | 0,50866 | 1 | 0,49599 | 0,58001 |
| RPTOR | rs8182275 | 78799864 | normSNP | 0 | C/T | 68,8 | 0,027446 | 0,30441 | 0,12969 | 1 | 0,13384 | 0,18914 |
| RPTOR | rs12947198 | 78801227 | creSNP | 0 | C/A | 80,1 | 1 | 0,69693 | 0,65754 | 0,55323 | 0,49599 | 0,8476 |
| RPTOR | rs118003029 | 78801680 | creSNP | 0 | G/A | 86,9 | 1 | 0,46622 | - | - | - | - |
| RPTOR | rs28742979 | 78801687 | creSNP | 0 | A/G | 77,3 | 1 | 0,90392 | 0,66449 | 1 | 0,65754 | 0,71408 |
| RPTOR | rs62069421 | 78801703 | creSNP | 0 | T/C | 80,1 | 1 | 0,69693 | 0,65754 | 0,55323 | 0,49599 | 0,8476 |
| RPTOR | rs62069422 | 78801772 | creSNP | 0 | A/G | 80,1 | 1 | 0,69693 | 0,65754 | 0,55323 | 0,49599 | 0,8476 |
| RPTOR | rs56238004 | 78801819 | creSNP | 0 | T/G | 80,1 | 1 | 0,69693 | 0,65754 | 0,55323 | 0,49599 | 0,8476 |
| RPTOR | rs56119556 | 78801985 | creSNP | 0 | C/T | 80,1 | 1 | 0,69693 | 0,65754 | 0,55323 | 0,49599 | 0,8476 |
| RPTOR | rs72856006 | 78802198 | creSNP | 0 | C/G | 88,6 | 1 | 0,60518 | 0,43624 | 1 | 0,59681 | 0,60518 |
| RPTOR | - | 78802650 | normSNP | 0 | A/C | 82,4 | 1 | 0,40162 | 0,82058 | 0,49425 | 0,48769 | 0,40162 |
| RPTOR | rs7210110 | 78803150 | normSNP | 0 | G/A | 79 | 1 | 0,78875 | 0,50866 | 1 | 0,49599 | 0,58001 |
| RPTOR | rs7216306 | 78803575 | normSNP | 0 | A/G | 80,7 | 1 | 0,58533 | 0,50293 | 0,55323 | 0,35932 | 0,69946 |
| RPTOR | rs72856013 | 78803597 | normSNP | 0 | G/A | 88,1 | 1 | 0,446 | 0,30762 | 1 | 0,43624 | 0,446 |
| RPTOR | rs12942555 | 78804085 | normSNP | 0 | A/G | 80,7 | 1 | 0,58533 | 0,50293 | 0,55323 | 0,35932 | 0,69946 |
| RPTOR | rs35629006 | 78804700 | normSNP | 0 | G/A | 81,2 | 1 | 0,68977 | 0,65278 | 0,55323 | 0,48769 | 0,84631 |
| RPTOR | rs35220590 | 78805491 | normSNP | 0 | A/G | 81,2 | 0,725846 | 0,79102 | 0,50293 | 1 | 0,49599 | 0,54508 |
| RPTOR | - | 78805643 | normSNP | 0 | A/G | 83 | 0,285634 | 0,65278 | - | - | - | - |
| RPTOR | rs11280580 | 78805653 | normSNP | 0 | A/G | 51,7 | 0 | 0,23037 | 0,33085 | 0,12995 | 0,79553 | 0,10322 |
| RPTOR | rs35867023 | 78806000 | normSNP | 0 | G/A | 71,6 | 0,429777 | 0,12739 | 0,28578 | 0,15362 | 0,08586 | 0,72189 |
| RPTOR | rs28377011 | 78806027 | creSNP | 0 | T/C | 86,9 | 0,633425 | 0,96677 | 0,80249 | 1 | 0,79553 | 0,82718 |
| RPTOR | rs2672871 | 78806313 | creSNP | 0 | G/C | 83,5 | 0,694991 | 0,26114 | 1 | 0,24138 | 0,46622 | 0,26114 |
| RPTOR | rs11395060 | 78806916 | normSNP | 0 | A/C | 80,7 | 0,297649 | 0,15319 | 0,11107 | 0,64411 | 0,05373 | 0,27823 |
| RPTOR | rs10468605 | 78807502 | creSNP | 0 | T/C | 59,1 | 0,514093 | 0,01321 | 0,11107 | 0,00483 | 0,66944 | 0,00943 |
| RPTOR | rs4270238 | 78808012 | creSNP | 0 | T/G | 58 | 0,661265 | 0,00571 | 0,06568 | 0,00232 | 0,66944 | 0,00366 |
| RPTOR | rs908232 | 78809293 | normSNP | 0 | A/G | 58,5 | 0,667331 | 0,00455 | 0,04 | 0,00232 | 0,83112 | 0,00232 |
| RPTOR | rs2333989 | 78809403 | normSNP | 0 | G/C | 59,7 | 0,660254 | 0,01088 | 0,07072 | 0,00483 | 0,83112 | 0,00613 |
| RPTOR | rs200435195 | 78809868 | normSNP | 0 | A/C | 52,8 | 0,668263 | 0,00543 | 0,00125 | 0,28883 | 0,05358 | 0,00682 |
| RPTOR | rs2138117 | 78809880 | normSNP | 0 | G/A | 58,5 | 0,667331 | 0,00455 | 0,04 | 0,00232 | 0,83112 | 0,00232 |
| RPTOR | rs2138116 | 78809884 | normSNP | 0 | T/C | 58,5 | 0,667331 | 0,00455 | 0,04 | 0,00232 | 0,83112 | 0,00232 |
| RPTOR | rs2176030 | 78809957 | normSNP | 0 | A/G | 58,5 | 0,667331 | 0,00455 | 0,04 | 0,00232 | 0,83112 | 0,00232 |
| RPTOR | rs2589143 | 78810687 | normSNP | 0 | G/T | 77,8 | 0,756052 | 0,0386 | 0,07879 | 0,05544 | 0,49599 | 0,0386 |
| RPTOR | rs117878919 | 78811073 | normSNP | 0 | G/A | 87,5 | 1 | 0,79973 | 0,80249 | 1 | 0,61069 | 0,79973 |
| RPTOR | rs2589144 | 78811244 | normSNP | 0 | C/T | 85,8 | 1 | 0,09272 | 0,05373 | 1 | 0,08749 | 0,09272 |
| RPTOR | rs746405 | 78812175 | normSNP | 0 | T/G | 56,8 | 0,828904 | 0,02204 | 0,01179 | 0,0557 | 0,39295 | 0,00611 |
| RPTOR | - | 78813370 | normSNP | 0 | A/C | 69,3 | 0,000024 | 0,18823 | 0,82902 | 0,17437 | 0,12463 | 0,58896 |
| RPTOR | rs146204651 | 78814113 | normSNP | 0 | A/C | 86,9 | 1 | 0,80598 | 0,62227 | 1 | 0,80249 | 0,80598 |
| RPTOR | rs146029097 | 78814152 | normSNP | 0 | A/C | 80,7 | 0,297649 | 0,82483 | 0,82058 | 0,64411 | 0,63199 | 1 |
| RPTOR | rs2672870 | 78814193 | normSNP | 0 | C/T | 50,6 | 0 | 0,87469 | 0,83078 | 0,66847 | 0,69315 | 0,73884 |
| RPTOR | rs12944497 | 78814197 | normSNP | 0 | T/C | 75 | 0 | 0,73743 | 0,48769 | 0,7871 | 0,50044 | 0,59273 |
| RPTOR | rs34415141 | 78815929 | normSNP | 0 | G/T | 86,9 | 1 | 0,80598 | 0,62227 | 1 | 0,80249 | 0,80598 |
| RPTOR | rs9897826 | 78816156 | normSNP | 0 | C/T | 85,2 | 1 | 0,08169 | 0,05373 | 0,49425 | 0,1377 | 0,08169 |
| RPTOR | rs7219745 | 78816908 | normSNP | 0 | A/C | 85,8 | 0,679266 | 0,12683 | 0,08749 | 0,49425 | 0,20941 | 0,12683 |
| RPTOR | rs35495979 | 78817027 | normSNP | 0 | C/T | 76,1 | 1 | 0,09277 | 0,5169 | 0,05544 | 0,65754 | 0,09277 |
| RPTOR | rs2672900 | 78818367 | normSNP | 0 | G/A | 86,9 | 1 | 0,80598 | 0,62227 | 1 | 0,80249 | 0,80598 |
| RPTOR | rs2589158 | 78818603 | normSNP | 0 | A/G | 85,8 | 0,367167 | 0,82183 | 1 | 0,55323 | 0,79553 | 0,83788 |
| RPTOR | rs4969219 | 78819890 | creSNP | 0 | T/G | 60,8 | 0,370164 | 0,01494 | 0,02351 | 0,01951 | 0,5213 | 0,00438 |
| RPTOR | rs2589157 | 78820023 | creSNP | 0 | C/T | 86,9 | 0,633425 | 0,05273 | 0,02262 | 0,49425 | 0,06712 | 0,05273 |
| RPTOR | rs3829572 | 78820212 | creSNP | 0 | A/G | 60,8 | 0,654387 | 0,05002 | 0,04345 | 0,05731 | 0,52216 | 0,01487 |
| RPTOR | rs3751945 | 78820329 | creSNP | 0 | T/C | 61,4 | 0,498878 | 0,02287 | 0,04345 | 0,01951 | 0,66944 | 0,00793 |
| RPTOR | rs2589156 | 78820374 | creSNP | 0 | G/A | 86,9 | 1 | 0,80598 | 0,62227 | 1 | 0,80249 | 0,80598 |
| RPTOR | rs2248502 | 78821572 | normSNP | 0 | G/A | 83 | 0,448557 | 0,36451 | 0,25593 | 1 | 0,35932 | 0,36451 |
| RPTOR | rs35074002 | 78821704 | normSNP | 0 | T/C | 60,2 | 0,505336 | 0,00953 | 0,02351 | 0,00983 | 0,66944 | 0,00304 |
| RPTOR | rs2589155 | 78822217 | normSNP | 0 | A/G | 85,8 | 0,367167 | 0,0739 | 0,0469 | 0,24138 | 0,19307 | 0,0739 |
| RPTOR | rs2589154 | 78822546 | normSNP | 0 | A/G | 81,8 | 0,284578 | 0,65574 | 0,50293 | 1 | 0,65278 | 0,65574 |
| RPTOR | rs2589153 | 78822589 | normSNP | 0 | G/A | 86,9 | 0,633425 | 0,11573 | 0,07775 | 0,49425 | 0,19307 | 0,11573 |
| RPTOR | rs2672899 | 78822910 | normSNP | 0 | C/G | 85,8 | 0,367167 | 0,0739 | 0,0469 | 0,24138 | 0,19307 | 0,0739 |
| RPTOR | rs2589151 | 78822959 | normSNP | 0 | A/G | 81,8 | 0,284578 | 0,65574 | 0,50293 | 1 | 0,65278 | 0,65574 |
| RPTOR | rs2589150 | 78823858 | normSNP | 0 | A/C | 85,8 | 0,679266 | 0,29133 | 0,22367 | 0,49425 | 0,45249 | 0,29133 |
| RPTOR | rs2672898 | 78824278 | normSNP | 0 | T/C | 77,8 | 0,545987 | 0,14918 | 0,19248 | 0,24138 | 0,50866 | 0,14918 |
| RPTOR | rs1040127 | 78824287 | normSNP | 0 | C/T | 86,9 | 1 | 0,80598 | 0,62227 | 1 | 0,80249 | 0,80598 |
| RPTOR | rs2672897 | 78824365 | normSNP | 0 | A/G | 78,4 | 0,75223 | 0,09882 | 0,12636 | 0,24138 | 0,37493 | 0,09882 |
| RPTOR | rs2672896 | 78824803 | normSNP | 0 | A/G | 78,4 | 0,75223 | 0,09882 | 0,12636 | 0,24138 | 0,37493 | 0,09882 |
| RPTOR | - | 78825300 | normSNP | 0 | A/C | 68,2 | 0,448635 | 1 | - | - | - | - |
| RPTOR | rs2589149 | 78826134 | normSNP | 0 | G/A | 86,9 | 1 | 0,80598 | 0,62227 | 1 | 0,80249 | 0,80598 |
| RPTOR | rs2672895 | 78826211 | normSNP | 0 | C/G | 86,9 | 1 | 0,80598 | 0,62227 | 1 | 0,80249 | 0,80598 |
| RPTOR | rs2589148 | 78826217 | normSNP | 0 | C/A | 86,4 | 0,654989 | 0,405 | 0,32371 | 0,49425 | 0,61069 | 0,405 |
| RPTOR | rs2063787 | 78827002 | normSNP | 0 | G/A | 87,5 | 1 | 1 | 0,80249 | 1 | 1 | 1 |
| RPTOR | rs2063786 | 78827062 | normSNP | 0 | T/C | 83,5 | 0,050842 | 0,09823 | 0,33749 | 0,05544 | 0,79553 | 0,09823 |
| RPTOR | rs2245187 | 78827124 | normSNP | 0 | C/T | 83 | 0,00027 | 0,04241 | 0,0469 | 0,01888 | 0,55939 | 0,0164 |
| RPTOR | rs2589119 | 78827428 | normSNP | 0 | G/A | 86,9 | 0,633425 | 0,11573 | 0,07775 | 0,49425 | 0,19307 | 0,11573 |
| RPTOR | rs2672894 | 78827615 | normSNP | 0 | G/A | 57,4 | 0,083292 | 0,45057 | 0,27021 | 0,30762 | 0,82757 | 0,20672 |
| RPTOR | rs2672893 | 78827863 | normSNP | 0 | G/A | 83,5 | 1 | 0,02436 | 0,01015 | 0,49425 | 0,0319 | 0,02436 |
| RPTOR | rs10871489 | 78827993 | normSNP | 0 | A/G | 71,6 | 0,429777 | 0,00967 | 0,01836 | 0,05544 | 0,19823 | 0,00967 |
| RPTOR | rs4969282 | 78828266 | normSNP | 0 | G/T | 85,2 | 0,682452 | 0,05745 | 0,0319 | 1 | 0,05373 | 0,05745 |
| RPTOR | rs4969220 | 78828353 | normSNP | 0 | G/A | 94,9 | 0,280048 | 0,72474 | - | - | - | - |
| RPTOR | rs12943041 | 78829375 | normSNP | 0 | G/A | 73,3 | 1 | 0,16129 | 0,83078 | 0,07799 | 0,2755 | 0,60613 |
| RPTOR | rs9895380 | 78829484 | normSNP | 0 | T/G | 69,9 | 0,615285 | 0,52812 | 1 | 0,28706 | 0,51329 | 0,63113 |
| RPTOR | rs17848633 | 78829531 | normSNP | 0 | C/T | 72,7 | 1 | 0,18671 | 1 | 0,07799 | 0,38541 | 0,49107 |
| RPTOR | rs35352641 | 78829856 | normSNP | 0 | C/T | 73,3 | 1 | 0,16129 | 0,83078 | 0,07799 | 0,2755 | 0,60613 |
| RPTOR | rs7218598 | 78830079 | creSNP | 0 | T/C | 69,3 | 0,801789 | 0,56163 | 0,83112 | 0,28706 | 0,66449 | 0,52073 |
| RPTOR | rs2672891 | 78831983 | normSNP | 0 | C/T | 73,3 | 0,78468 | 0,0626 | 0,66847 | 0,03876 | 0,12215 | 0,61636 |
| RPTOR | rs56233717 | 78833745 | normSNP | 0 | T/C | 68,2 | 1 | 0,56296 | 0,83078 | 0,28706 | 0,66683 | 0,51803 |
| RPTOR | rs56296322 | 78833955 | normSNP | 0 | G/A | 68,8 | 0,807538 | 0,56505 | 0,66944 | 0,28706 | 0,82902 | 0,42044 |
| RPTOR | rs2672890 | 78834207 | normSNP | 0 | C/T | 85,2 | 1 | 0,88489 | 0,63199 | 1 | 0,62227 | 0,67183 |
| RPTOR | rs908241 | 78835678 | creSNP | 0 | G/A | 88,6 | 1 | 0,60518 | 0,43624 | 1 | 0,59681 | 0,60518 |
| RPTOR | rs4586503 | 78836386 | creSNP | 0 | G/A | 76,7 | 0,018766 | 0,83078 | - | - | - | - |
| RPTOR | rs2589118 | 78836553 | normSNP | 0 | T/C | 54 | 0,292422 | 0,23925 | 0,80828 | 0,09388 | 0,28274 | 0,25758 |
| RPTOR | rs2063785 | 78836846 | normSNP | 0 | A/G | 72,7 | 1 | 0,21128 | 0,66944 | 0,07799 | 0,66449 | 0,30097 |
| RPTOR | rs80014805 | 78837793 | normSNP | 0 | A/G | 86,9 | 0,633425 | 0,11573 | 0,07775 | 0,49425 | 0,19307 | 0,11573 |
| RPTOR | rs4969221 | 78837799 | normSNP | 0 | A/G | 81,2 | 0,171764 | 0,12919 | 0,06568 | 0,15362 | 0,22367 | 0,04376 |
| RPTOR | rs72490481 | 78837996 | normSNP | 0 | A/G | 76,1 | 1 | 0,84112 | 0,82902 | 0,64411 | 0,65754 | 1 |
| RPTOR | rs4969222 | 78838122 | normSNP | 0 | G/A | 89,8 | 1 | 0,40773 | 0,41692 | 1 | 0,26682 | 0,40773 |
| RPTOR | rs2256433 | 78838126 | normSNP | 0 | T/C | 97,2 | 0,115274 | 0,05544 | - | - | - | - |
| RPTOR | rs1964760 | 78838141 | normSNP | 0 | T/C | 85,8 | 0,681984 | 0,81312 | - | - | - | - |
| RPTOR | rs2014851 | 78838172 | normSNP | 0 | A/G | 67,6 | 0,633079 | 0,68705 | 0,83008 | 0,45614 | 0,5213 | 0,86777 |
| RPTOR | rs2014852 | 78838181 | normSNP | 0 | A/G | 82,4 | 0,724368 | 0,82704 | 1 | 0,55323 | 0,81312 | 0,84473 |
| RPTOR | rs74534309 | 78838310 | normSNP | 0 | G/T | 70,5 | 0,076431 | 0,68222 | 0,39146 | 1 | 0,39344 | 0,45919 |
| RPTOR | rs75817578 | 78838311 | normSNP | 0 | A/T | 70,5 | 0,076431 | 0,68222 | 0,39146 | 1 | 0,39344 | 0,45919 |
| RPTOR | rs12939768 | 78839202 | normSNP | 0 | A/T | 71,6 | 0,792807 | 0,20218 | 0,20012 | 0,39341 | 0,08417 | 0,48962 |
| RPTOR | rs11651493 | 78840038 | creSNP | 0 | G/A | 54 | 1 | 0,64405 | 0,34928 | 0,79553 | 0,52216 | 0,45277 |
| RPTOR | rs11651587 | 78840250 | creSNP | 0 | G/A | 54 | 1 | 0,64405 | 0,34928 | 0,79553 | 0,52216 | 0,45277 |
| RPTOR | rs7210951 | 78840944 | normSNP | 0 | A/T | 58 | 1 | 0,52276 | 0,25593 | 0,77674 | 0,39344 | 0,3524 |
| RPTOR | rs9912092 | 78841693 | normSNP | 0 | A/G | 56,8 | 1 | 0,38471 | 0,16844 | 0,58001 | 0,39344 | 0,21765 |
| RPTOR | rs529458157 | 78842961 | normSNP | 0 | G/A | 69,3 | 0,62176 | 0,43648 | 0,28477 | 0,69315 | 0,19823 | 0,49768 |
| RPTOR | rs59453935 | 78842999 | normSNP | 0 | G/T | 72,2 | 0,432002 | 0,08875 | 0,20012 | 0,15362 | 0,05255 | 0,594 |
| RPTOR | rs2589141 | 78843139 | normSNP | 0 | T/C | 80,1 | 0,505387 | 0,58397 | 0,82571 | 0,49425 | 0,8234 | 0,58397 |
| RPTOR | rs34576464 | 78843386 | normSNP | 0 | C/G | 72,2 | 0,794069 | 0,27684 | 0,28578 | 0,39341 | 0,12969 | 0,60517 |
| RPTOR | rs34189581 | 78844235 | normSNP | 0 | G/T | 72,2 | 0,794069 | 0,27684 | 0,28578 | 0,39341 | 0,12969 | 0,60517 |
| RPTOR | rs2589142 | 78844262 | normSNP | 0 | G/A | 70,5 | 1 | 0,43227 | 0,28578 | 0,69315 | 0,19585 | 0,50018 |
| RPTOR | rs36022926 | 78844667 | normSNP | 0 | A/T | 69,3 | 0,62176 | 0,43648 | 0,28477 | 0,69315 | 0,19823 | 0,49768 |
| RPTOR | rs12150252 | 78845352 | normSNP | 0 | C/T | 72,7 | 0,590502 | 0,42541 | 0,28578 | 0,64411 | 0,19585 | 0,47774 |
| RPTOR | rs12150256 | 78845366 | normSNP | 0 | C/G | 72,2 | 0,794069 | 0,27684 | 0,28578 | 0,39341 | 0,12969 | 0,60517 |
| RPTOR | rs201987756 | 78846909 | normSNP | 0 | A/C | 74,4 | 0,573458 | 0,29237 | 0,19585 | 0,69315 | 0,11706 | 0,40375 |
| RPTOR | rs9912406 | 78847068 | normSNP | 0 | C/T | 70,5 | 1 | 0,43227 | 0,28578 | 0,69315 | 0,19585 | 0,50018 |
| RPTOR | rs34410650 | 78847595 | normSNP | 0 | A/G | 71,6 | 0,792807 | 0,20218 | 0,20012 | 0,39341 | 0,08417 | 0,48962 |
| RPTOR | rs6565484 | 78848856 | normSNP | 0 | C/T | 56,8 | 0,828904 | 0,52028 | 0,25593 | 0,7871 | 0,39295 | 0,36695 |
| RPTOR | rs34072556 | 78849025 | normSNP | 0 | C/G | 72,2 | 0,794069 | 0,27684 | 0,28578 | 0,39341 | 0,12969 | 0,60517 |
| RPTOR | rs6565485 | 78849211 | normSNP | 0 | A/G | 60,2 | 1 | 0,81392 | 0,65754 | 0,55939 | 1 | 0,53832 |
| RPTOR | rs11651707 | 78849310 | normSNP | 0 | C/T | 72,7 | 0,279925 | 0,31442 | 0,39344 | 0,29556 | 0,19823 | 0,71409 |
| RPTOR | rs112957845 | 78850056 | normSNP | 0 | G/A | 71,6 | 0,000465 | 0,67815 | 0,38541 | 0,55939 | 0,62227 | 0,39344 |
| RPTOR | rs183706698 | 78850337 | normSNP | 0 | T/C | 74,4 | 1 | 0,44399 | 0,51955 | 0,39341 | 0,27021 | 0,8638 |
| RPTOR | rs75032657 | 78850352 | normSNP | 0 | C/G | 72,2 | 0,794069 | 0,45898 | 0,52216 | 0,39341 | 0,27966 | 0,86326 |
| RPTOR | rs71272877 | 78850503 | normSNP | 0 | A/G | 72,7 | 0,279925 | 0,31442 | 0,39344 | 0,29556 | 0,19823 | 0,71409 |
| RPTOR | rs8074030 | 78850880 | normSNP | 0 | C/T | 57,4 | 0,665816 | 0,66581 | 0,36782 | 0,7871 | 0,5213 | 0,45599 |
| RPTOR | rs11659058 | 78850900 | normSNP | 0 | A/T | 72,7 | 0,279925 | 0,31442 | 0,39344 | 0,29556 | 0,19823 | 0,71409 |
| RPTOR | rs35177169 | 78850931 | normSNP | 0 | G/A | 72,7 | 0,279925 | 0,31442 | 0,39344 | 0,29556 | 0,19823 | 0,71409 |
| RPTOR | rs8068506 | 78850952 | normSNP | 0 | G/A | 70,5 | 1 | 0,43227 | 0,28578 | 0,69315 | 0,19585 | 0,50018 |
| RPTOR | rs67726311 | 78851206 | normSNP | 0 | T/C | 72,2 | 0,187315 | 0,39313 | 0,52216 | 0,29556 | 0,28477 | 0,85444 |
| RPTOR | rs34411287 | 78851303 | normSNP | 0 | A/G | 72,7 | 0,279925 | 0,31442 | 0,39344 | 0,29556 | 0,19823 | 0,71409 |
| RPTOR | rs34613006 | 78851474 | normSNP | 0 | T/C | 72,7 | 0,279925 | 0,31442 | 0,39344 | 0,29556 | 0,19823 | 0,71409 |
| RPTOR | rs35057406 | 78851527 | normSNP | 0 | A/G | 72,2 | 0,187315 | 0,39313 | 0,52216 | 0,29556 | 0,28477 | 0,85444 |
| RPTOR | rs7222366 | 78851634 | normSNP | 0 | C/T | 57,4 | 0,665816 | 0,66581 | 0,36782 | 0,7871 | 0,5213 | 0,45599 |
| RPTOR | rs7217762 | 78851762 | normSNP | 0 | C/T | 72,2 | 0,794069 | 0,27684 | 0,28578 | 0,39341 | 0,12969 | 0,60517 |
| RPTOR | rs34206260 | 78851842 | normSNP | 0 | T/C | 72,2 | 0,187315 | 0,39313 | 0,52216 | 0,29556 | 0,28477 | 0,85444 |
| RPTOR | rs11655385 | 78852170 | normSNP | 0 | G/A | 72,2 | 0,187315 | 0,39313 | 0,52216 | 0,29556 | 0,28477 | 0,85444 |
| RPTOR | rs11150752 | 78852408 | normSNP | 0 | A/C | 72,7 | 0,279925 | 0,31442 | 0,39344 | 0,29556 | 0,19823 | 0,71409 |
| RPTOR | rs2878074 | 78852588 | normSNP | 0 | A/C | 71,6 | 0,792807 | 0,20218 | 0,20012 | 0,39341 | 0,08417 | 0,48962 |
| RPTOR | rs11150753 | 78852829 | normSNP | 0 | G/A | 72,7 | 0,279925 | 0,31442 | 0,39344 | 0,29556 | 0,19823 | 0,71409 |
| RPTOR | rs11150754 | 78852913 | normSNP | 0 | C/T | 72,7 | 0,279925 | 0,31442 | 0,39344 | 0,29556 | 0,19823 | 0,71409 |
| RPTOR | rs11150755 | 78853076 | normSNP | 0 | C/T | 72,7 | 0,279925 | 0,31442 | 0,39344 | 0,29556 | 0,19823 | 0,71409 |
| RPTOR | rs752754 | 78853571 | normSNP | 0 | A/G | 72,7 | 0,279925 | 0,31442 | 0,39344 | 0,29556 | 0,19823 | 0,71409 |
| RPTOR | rs6420478 | 78854056 | normSNP | 0 | G/A | 58,5 | 0,667331 | 0,49088 | 0,25593 | 1 | 0,28578 | 0,43115 |
| RPTOR | rs2289759 | 78854223 | normSNP | 0 | A/G | 72,7 | 0,279925 | 0,31442 | 0,39344 | 0,29556 | 0,19823 | 0,71409 |
| RPTOR | rs2289760 | 78854316 | normSNP | 0 | C/T | 72,2 | 0,432002 | 0,54654 | 0,39344 | 0,64411 | 0,28274 | 0,594 |
| RPTOR | rs2248843 | 78854487 | normSNP | 0 | G/A | 58,5 | 0,667331 | 0,49088 | 0,25593 | 1 | 0,28578 | 0,43115 |
| RPTOR | rs12937510 | 78854496 | normSNP | 0 | C/T | 72,7 | 0,279925 | 0,31442 | 0,39344 | 0,29556 | 0,19823 | 0,71409 |
| RPTOR | rs2589121 | 78854848 | normSNP | 0 | T/A | 68,8 | 1 | 0,50463 | 0,5213 | 0,45614 | 0,28274 | 0,86875 |
| RPTOR | - | 78855015 | normSNP | 0 | A/C | 80,1 | 0,096563 | 0,38139 | 0,49599 | 0,39341 | 0,22367 | 0,86196 |
| RPTOR | rs2589122 | 78855091 | normSNP | 0 | A/G | 59,1 | 0,827931 | 0,63793 | 0,36782 | 1 | 0,39344 | 0,5323 |
| RPTOR | - | 78857376 | normSNP | 0 | A/C | 71 | 1 | 0,55288 | 0,39344 | 0,69315 | 0,27966 | 0,61412 |
| RPTOR | rs11656246 | 78857885 | normSNP | 0 | G/A | 72,7 | 0,279925 | 0,31442 | 0,39344 | 0,29556 | 0,19823 | 0,71409 |
| RPTOR | rs2672874 | 78858122 | normSNP | 0 | A/T | 58,5 | 1 | 0,66661 | 0,36782 | 0,77674 | 0,52216 | 0,44227 |
| RPTOR | rs2672875 | 78858180 | normSNP | 0 | A/G | 58,5 | 1 | 0,66661 | 0,36782 | 0,77674 | 0,52216 | 0,44227 |
| RPTOR | rs2589123 | 78858249 | normSNP | 0 | C/T | 58,5 | 1 | 0,66661 | 0,36782 | 0,77674 | 0,52216 | 0,44227 |
| RPTOR | rs67579373 | 78858251 | normSNP | 0 | T/C | 58 | 1 | 0,52276 | 0,25593 | 0,77674 | 0,39344 | 0,3524 |
| RPTOR | rs2589124 | 78858426 | normSNP | 0 | C/T | 59,7 | 0,506759 | 0,7598 | 0,50866 | 0,58001 | 0,83008 | 0,45915 |
| RPTOR | rs2589125 | 78858438 | normSNP | 0 | T/C | 68,8 | 1 | 0,30424 | 0,28477 | 0,45614 | 0,13218 | 0,6199 |
| RPTOR | rs2672877 | 78858662 | normSNP | 0 | G/A | 58 | 1 | 0,52276 | 0,25593 | 0,77674 | 0,39344 | 0,3524 |
| RPTOR | rs2016817 | 78859004 | normSNP | 0 | C/T | 72,7 | 0,279925 | 0,46345 | 0,66976 | 0,29556 | 0,39146 | 1 |
| RPTOR | rs2289762 | 78859110 | normSNP | 0 | G/A | 72,2 | 0,187315 | 0,39313 | 0,52216 | 0,29556 | 0,28477 | 0,85444 |
| RPTOR | rs56283765 | 78859230 | normSNP | 0 | C/T | 72,2 | 0,794069 | 0,27684 | 0,28578 | 0,39341 | 0,12969 | 0,60517 |
| RPTOR | rs2004184 | 78859501 | normSNP | 0 | T/C | 70,5 | 0,121077 | 0,19235 | 0,13384 | 0,74706 | 0,07072 | 0,36082 |
| RPTOR | rs2672878 | 78859894 | normSNP | 0 | C/T | 70,5 | 0,209862 | 0,66945 | 0,5213 | 0,64411 | 0,39295 | 0,72054 |
| RPTOR | rs55823497 | 78860412 | normSNP | 0 | C/T | 72,2 | 0,794069 | 0,27684 | 0,28578 | 0,39341 | 0,12969 | 0,60517 |
| RPTOR | rs2589129 | 78860593 | normSNP | 0 | A/G | 57,4 | 1 | 0,48454 | 0,25593 | 1 | 0,28578 | 0,44567 |
| RPTOR | rs908235 | 78861457 | normSNP | 0 | G/A | 77,3 | 0,220091 | 0,20298 | 0,38896 | 0,49425 | 0,19248 | 0,20298 |
| RPTOR | rs200877069 | 78861683 | normSNP | 0 | A/C | 77,8 | 0,028826 | 0,08271 | 0,11706 | 0,45614 | 0,02736 | 0,41462 |
| RPTOR | rs62068460 | 78861801 | normSNP | 0 | C/T | 73,3 | 1 | 0,26955 | 0,28477 | 0,39341 | 0,12636 | 0,60613 |
| RPTOR | rs4969284 | 78862220 | normSNP | 0 | G/A | 54,5 | 0,672306 | 0,31389 | 0,81312 | 0,17437 | 0,19965 | 0,53321 |
| RPTOR | rs2672881 | 78863041 | normSNP | 0 | G/C | 52,3 | 0,52088 | 0,1686 | 0,62227 | 0,11004 | 0,08586 | 0,5258 |
| RPTOR | rs2063788 | 78863298 | normSNP | 0 | C/G | 54,5 | 0,672306 | 0,31389 | 0,81312 | 0,17437 | 0,19965 | 0,53321 |
| RPTOR | rs2063789 | 78863360 | normSNP | 0 | C/A | 54,5 | 0,672306 | 0,31389 | 0,81312 | 0,17437 | 0,19965 | 0,53321 |
| RPTOR | rs2063790 | 78863578 | normSNP | 0 | G/A | 54 | 0,833432 | 0,47401 | 0,81312 | 0,28883 | 0,28578 | 0,64503 |
| RPTOR | rs2589130 | 78863660 | normSNP | 0 | C/T | 71,6 | 0,062947 | 0,88055 | 0,83008 | 0,74706 | 0,64704 | 1 |
| RPTOR | rs28374944 | 78863770 | normSNP | 0 | C/T | 55,7 | 0,829038 | 0,52785 | 1 | 0,28883 | 0,39295 | 0,55011 |
| RPTOR | rs71354838 | 78863775 | normSNP | 0 | G/A | 60,8 | 1 | 0,30831 | 0,18267 | 0,76375 | 0,13466 | 0,43374 |
| RPTOR | rs4969285 | 78863932 | normSNP | 0 | C/G | 73,3 | 0,78468 | 0,87941 | 0,66847 | 0,69315 | 0,82571 | 0,61636 |
| RPTOR | rs4969286 | 78864016 | normSNP | 0 | G/A | 54,5 | 0,672306 | 0,31389 | 0,81312 | 0,17437 | 0,19965 | 0,53321 |
| RPTOR | rs4969287 | 78864029 | normSNP | 0 | C/T | 54,5 | 0,672306 | 0,31389 | 0,81312 | 0,17437 | 0,19965 | 0,53321 |
| RPTOR | rs11870045 | 78864421 | normSNP | 0 | C/T | 55,7 | 0,391599 | 0,2878 | 0,81312 | 0,15304 | 0,19823 | 0,52074 |
| RPTOR | rs11656061 | 78864749 | normSNP | 0 | G/A | 55,1 | 0,523225 | 0,18338 | 0,81312 | 0,09388 | 0,13384 | 0,42906 |
| RPTOR | rs7221927 | 78865127 | normSNP | 0 | T/C | 69,9 | 0,615285 | 0,55095 | 0,39344 | 0,72474 | 0,2755 | 0,63113 |
| RPTOR | rs2672884 | 78865171 | normSNP | 0 | G/C | 75,6 | 1 | 0,89063 | 1 | 0,64411 | 0,82571 | 0,85965 |
| RPTOR | rs2672885 | 78865180 | normSNP | 0 | T/C | 76,1 | 1 | 0,8954 | 0,82902 | 0,64411 | 1 | 0,7234 |
| RPTOR | rs2289764 | 78865546 | normSNP | 0 | T/C | 77,8 | 0,350089 | 0,77968 | 0,50866 | 1 | 0,48769 | 0,60511 |
| RPTOR | rs2289765 | 78865630 | normSNP | 0 | A/G | 88,6 | 1 | 0,60518 | 0,43624 | 1 | 0,59681 | 0,60518 |
| RPTOR | rs2289766 | 78865824 | normSNP | 0 | A/G | 83,5 | 0,694991 | 0,8256 | 1 | 0,55323 | 0,80828 | 0,84283 |
| RPTOR | rs8064817 | 78865950 | normSNP | 0 | C/T | 83,5 | 0,694991 | 0,79939 | 0,64017 | 0,55323 | 0,80828 | 0,55149 |
| RPTOR | rs11868966 | 78865999 | normSNP | 0 | A/C | 85,8 | 0,367167 | 0,79729 | 0,62227 | 0,55323 | 0,79553 | 0,53872 |
| RPTOR | rs8069822 | 78866410 | normSNP | 0 | A/G | 79 | 0,518935 | 0,88763 | 1 | 0,64411 | 0,81718 | 0,85845 |
| RPTOR | rs2333987 | 78867041 | normSNP | 0 | C/T | 78,4 | 0,53643 | 0,89604 | 0,82571 | 0,64411 | 1 | 0,72187 |
| RPTOR | rs2333986 | 78867061 | normSNP | 0 | C/T | 79,5 | 0,342495 | 0,89633 | 0,8234 | 0,64411 | 1 | 0,7205 |
| RPTOR | rs2077148 | 78867123 | normSNP | 0 | C/T | 80,7 | 0,297649 | 0,89659 | 0,82058 | 0,64411 | 1 | 0,71873 |
| RPTOR | rs3042650 | 78867195 | normSNP | 0 | A/C | 88,1 | 0,016486 | 0,41496 | 0,7871 | 0,29556 | 0,36558 | 0,83818 |
| RPTOR | rs2333985 | 78867242 | normSNP | 0 | A/G | 83,5 | 0,050842 | 0,85363 | 0,63199 | 0,64411 | 0,79553 | 0,57986 |
| RPTOR | rs2333984 | 78867377 | normSNP | 0 | A/G | 82,4 | 0,130197 | 0,85491 | 0,64017 | 0,64411 | 0,80249 | 0,58395 |
| RPTOR | rs3751943 | 78867733 | normSNP | 0 | A/G | 76,1 | 0,560648 | 1 | 1 | 1 | 1 | 1 |
| RPTOR | rs17848656 | 78867822 | normSNP | 0 | C/T | 79 | 0,518935 | 0,88763 | 1 | 0,64411 | 0,81718 | 0,85845 |
| RPTOR | rs55929430 | 78867885 | normSNP | 0 | T/C | 79 | 0,518935 | 0,88763 | 1 | 0,64411 | 0,81718 | 0,85845 |
| RPTOR | rs55880615 | 78867906 | normSNP | 0 | G/A | 80,7 | 0,297649 | 0,89659 | 0,82058 | 0,64411 | 1 | 0,71873 |
| RPTOR | rs55777744 | 78867951 | normSNP | 0 | G/C | 79,5 | 0,342495 | 0,8299 | 0,8234 | 0,64411 | 0,64017 | 1 |
| RPTOR | rs7219554 | 78868185 | normSNP | 0 | A/G | 77,8 | 0,756052 | 0,88876 | 1 | 0,64411 | 0,82058 | 0,85905 |
| RPTOR | rs7503014 | 78868442 | normSNP | 0 | C/T | 81,8 | 0,147858 | 0,89684 | 0,81718 | 0,64411 | 1 | 0,71651 |
| RPTOR | rs6565486 | 78868450 | normSNP | 0 | C/A | 81,8 | 0,147858 | 0,89684 | 0,81718 | 0,64411 | 1 | 0,71651 |
| RPTOR | rs7503016 | 78868493 | normSNP | 0 | C/T | 78,4 | 0,53643 | 0,89604 | 0,82571 | 0,64411 | 1 | 0,72187 |
| RPTOR | rs4969288 | 78869003 | normSNP | 0 | G/A | 98,9 | 0 | 1 | - | - | - | - |
| RPTOR | rs4969289 | 78869374 | normSNP | 0 | A/G | 76,7 | 0,228113 | 0,91375 | 1 | 0,69315 | 0,81718 | 0,86744 |
| RPTOR | rs7502932 | 78869413 | normSNP | 0 | A/G | 77,3 | 0,368313 | 1 | 1 | 1 | 1 | 1 |
| RPTOR | rs7502953 | 78869492 | normSNP | 0 | A/G | 77,3 | 0,368313 | 1 | 1 | 1 | 1 | 1 |
| RPTOR | rs11150756 | 78869689 | normSNP | 0 | G/A | 80,1 | 0,316322 | 0,88632 | 1 | 0,64411 | 0,81312 | 0,85763 |
| RPTOR | rs7503238 | 78869717 | normSNP | 0 | G/A | 77,8 | 0,756052 | 0,88876 | 1 | 0,64411 | 0,82058 | 0,85905 |
| RPTOR | rs12948455 | 78869874 | normSNP | 0 | T/A | 77,8 | 0,756052 | 0,88876 | 1 | 0,64411 | 0,82058 | 0,85905 |
| RPTOR | rs59698542 | 78869992 | normSNP | 0 | A/G | 78,4 | 0,53643 | 0,89604 | 0,82571 | 0,64411 | 1 | 0,72187 |
| RPTOR | rs58504207 | 78870002 | normSNP | 0 | T/G | 78,4 | 0,53643 | 0,89604 | 0,82571 | 0,64411 | 1 | 0,72187 |
| RPTOR | rs61259441 | 78870054 | normSNP | 0 | A/G | 76,7 | 0,548234 | 0,97367 | 0,82757 | 1 | 0,82058 | 0,86362 |
| RPTOR | rs60189970 | 78870117 | normSNP | 0 | C/T | 77,3 | 0,764065 | 0,89573 | 0,82757 | 0,64411 | 1 | 0,72283 |
| RPTOR | rs56821198 | 78870160 | normSNP | 0 | G/A | 76,7 | 0,004937 | 0,96793 | 0,8234 | 1 | 0,80249 | 0,87721 |
| RPTOR | rs112808324 | 78870166 | normSNP | 0 | A/C | 65,9 | 0 | 0,65072 | 0,37493 | 0,35932 | 1 | 0,35531 |
| RPTOR | - | 78870358 | normSNP | 0 | A/C | 72,7 | 0,7908 | 0,08013 | 0,83078 | 0,03876 | 0,1881 | 0,50345 |
| RPTOR | rs7210331 | 78870640 | normSNP | 0 | C/T | 78,4 | 0,53643 | 0,89604 | 0,82571 | 0,64411 | 1 | 0,72187 |
| RPTOR | rs7225506 | 78870670 | normSNP | 0 | G/A | 77,3 | 0,368313 | 1 | 1 | 1 | 1 | 1 |
| RPTOR | rs7223865 | 78870711 | normSNP | 0 | T/G | 77,3 | 0,368313 | 1 | 1 | 1 | 1 | 1 |
| RPTOR | rs6565487 | 78870874 | normSNP | 0 | A/G | 68,8 | 0 | 0,90541 | 0,66142 | 0,80249 | 0,76375 | 0,70455 |
| RPTOR | rs5822373 | 78870902 | normSNP | 0 | A/C | 50 | 0,000002 | 0,88573 | 0,82571 | 0,82571 | 0,62227 | 1 |
| RPTOR | rs11652900 | 78871227 | normSNP | 0 | G/A | 79 | 0,518935 | 0,88763 | 1 | 0,64411 | 0,81718 | 0,85845 |
| RPTOR | rs10871490 | 78871354 | normSNP | 0 | T/C | 77,8 | 0,350089 | 0,97276 | 0,82571 | 1 | 0,81718 | 0,86325 |
| RPTOR | rs4969290 | 78871585 | normSNP | 0 | C/T | 78,4 | 0,53643 | 0,83422 | 0,82571 | 0,64411 | 0,64704 | 1 |
| RPTOR | rs4969291 | 78871992 | normSNP | 0 | C/T | 77,8 | 0,756052 | 0,88876 | 1 | 0,64411 | 0,82058 | 0,85905 |
| RPTOR | rs4969292 | 78872094 | normSNP | 0 | C/T | 77,8 | 0,756052 | 0,88876 | 1 | 0,64411 | 0,82058 | 0,85905 |
| RPTOR | rs4969293 | 78872101 | normSNP | 0 | T/A | 80,1 | 0,316322 | 0,88632 | 1 | 0,64411 | 0,81312 | 0,85763 |
| RPTOR | rs4969294 | 78872102 | normSNP | 0 | C/T | 80,1 | 0,316322 | 0,88632 | 1 | 0,64411 | 0,81312 | 0,85763 |
| RPTOR | rs4969224 | 78872645 | normSNP | 0 | C/T | 81,2 | 0,069828 | 0,96849 | 0,81718 | 1 | 0,80249 | 0,86101 |
| RPTOR | rs12232521 | 78872953 | normSNP | 0 | G/A | 78,4 | 0,53643 | 0,89604 | 0,82571 | 0,64411 | 1 | 0,72187 |
| RPTOR | rs7217623 | 78873014 | normSNP | 0 | G/A | 82,4 | 0,130197 | 0,85491 | 0,64017 | 0,64411 | 0,80249 | 0,58395 |
| RPTOR | rs4969295 | 78873206 | normSNP | 0 | T/A | 78,4 | 0,53643 | 0,83422 | 0,82571 | 0,64411 | 0,64704 | 1 |
| RPTOR | rs4969296 | 78873297 | normSNP | 0 | G/A | 81,2 | 0,069828 | 0,96849 | 0,81718 | 1 | 0,80249 | 0,86101 |
| RPTOR | rs4969297 | 78873464 | normSNP | 0 | G/A | 86,9 | 0,03454 | 0,69262 | 0,43624 | 1 | 0,39373 | 0,54762 |
| RPTOR | rs4969298 | 78873473 | normSNP | 0 | T/G | 84,1 | 0,446693 | 0,83708 | 0,81312 | 0,55323 | 1 | 0,68951 |
| RPTOR | rs4969299 | 78873719 | normSNP | 0 | C/T | 77,8 | 0,350089 | 0,97276 | 0,82571 | 1 | 0,81718 | 0,86325 |
| RPTOR | rs7222184 | 78873750 | normSNP | 0 | T/C | 77,8 | 0,350089 | 0,97276 | 0,82571 | 1 | 0,81718 | 0,86325 |
| RPTOR | rs7223334 | 78874038 | normSNP | 0 | G/A | 79 | 0,518935 | 0,88763 | 1 | 0,64411 | 0,81718 | 0,85845 |
| RPTOR | rs7222906 | 78874141 | normSNP | 0 | T/C | 80,7 | 0,080583 | 0,88305 | 0,64704 | 1 | 0,62227 | 0,72707 |
| RPTOR | rs7208146 | 78874167 | normSNP | 0 | C/T | 77,3 | 0,368313 | 1 | 1 | 1 | 1 | 1 |
| RPTOR | rs7221866 | 78874235 | normSNP | 0 | C/G | 77,3 | 0,368313 | 1 | 1 | 1 | 1 | 1 |
| RPTOR | rs7223646 | 78874242 | normSNP | 0 | G/A | 79,5 | 0,1819 | 1 | 1 | 1 | 1 | 1 |
| RPTOR | rs7225783 | 78874350 | normSNP | 0 | A/G | 77,3 | 0,368313 | 1 | 1 | 1 | 1 | 1 |
| RPTOR | rs7208148 | 78874368 | normSNP | 0 | G/A | 77,3 | 0,368313 | 1 | 1 | 1 | 1 | 1 |
| RPTOR | rs7226292 | 78874672 | normSNP | 0 | A/G | 77,8 | 0,350089 | 0,97276 | 0,82571 | 1 | 0,81718 | 0,86325 |
| RPTOR | rs7208835 | 78874759 | normSNP | 0 | G/A | 79,5 | 0,1819 | 1 | 1 | 1 | 1 | 1 |
| RPTOR | rs7208264 | 78874800 | normSNP | 0 | G/C | 77,3 | 0,368313 | 1 | 1 | 1 | 1 | 1 |
| RPTOR | rs7213674 | 78875183 | normSNP | 0 | C/T | 79 | 0,518935 | 0,88763 | 1 | 0,64411 | 0,81718 | 0,85845 |
| RPTOR | rs7502391 | 78875303 | normSNP | 0 | C/T | 81,8 | 0,147858 | 0,89684 | 0,81718 | 0,64411 | 1 | 0,71651 |
| RPTOR | rs2280144 | 78875611 | normSNP | 0 | A/G | 76,7 | 0,548234 | 0,97367 | 0,82757 | 1 | 0,82058 | 0,86362 |
| RPTOR | rs67761192 | 78875620 | normSNP | 0 | A/G | 77,3 | 0,368313 | 1 | 1 | 1 | 1 | 1 |
| RPTOR | rs2280146 | 78875669 | normSNP | 0 | A/G | 79,5 | 0,1819 | 1 | 1 | 1 | 1 | 1 |
| RPTOR | rs2280147 | 78876227 | normSNP | 0 | T/C | 75 | 0,158857 | 1 | 1 | 1 | 1 | 1 |
| RPTOR | rs868432 | 78877735 | normSNP | 0 | G/A | 51,7 | 0,833064 | 0,45423 | 0,63199 | 0,20941 | 0,52216 | 0,29518 |
| RPTOR | rs4969300 | 78877789 | normSNP | 0 | G/A | 89,8 | 1 | 0,59017 | 0,41692 | 1 | 0,58001 | 0,59017 |
| RPTOR | rs9898178 | 78878937 | normSNP | 0 | A/G | 84,7 | 1 | 0,23738 | 0,47784 | 0,49425 | 0,22367 | 0,23738 |
| RPTOR | - | 78878979 | normSNP | 0 | A/C | 67,6 | 0 | 0,93729 | 0,82571 | 1 | 0,72474 | 0,90353 |
| RPTOR | rs62068487 | 78879035 | normSNP | 0 | G/A | 76,7 | 1 | 0,85618 | 0,66449 | 0,64411 | 0,8234 | 0,59502 |
| RPTOR | rs61124969 | 78879128 | normSNP | 0 | G/A | 64,8 | 0,243117 | 0,317 | 0,66142 | 0,12995 | 0,66944 | 0,30461 |
| RPTOR | rs11652465 | 78879613 | normSNP | 0 | G/C | 61,9 | 0,042888 | 0,316 | 0,82058 | 0,12995 | 0,5169 | 0,3758 |
| RPTOR | rs12951596 | 78880383 | normSNP | 0 | C/T | 86,4 | 1 | 0,21792 | 0,22367 | 1 | 0,1377 | 0,21792 |
| RPTOR | rs12950583 | 78881342 | normSNP | 0 | G/A | 77,3 | 1 | 0,4438 | 0,66449 | 0,29556 | 0,37493 | 1 |
| RPTOR | rs7216750 | 78881927 | normSNP | 0 | G/A | 63,1 | 0,107553 | 0,31521 | 0,8234 | 0,12995 | 0,51955 | 0,38318 |
| RPTOR | rs2063791 | 78882329 | normSNP | 0 | G/A | 62,5 | 0,068563 | 0,29919 | 1 | 0,12995 | 0,38896 | 0,4824 |
| RPTOR | rs1468029 | 78882806 | normSNP | 0 | G/A | 63,1 | 0,107553 | 0,31521 | 0,8234 | 0,12995 | 0,51955 | 0,38318 |
| RPTOR | rs6420479 | 78882825 | normSNP | 0 | G/A | 86,4 | 1 | 0,21792 | 0,22367 | 1 | 0,1377 | 0,21792 |
| RPTOR | rs1468030 | 78882840 | normSNP | 0 | A/G | 62,5 | 0,173224 | 0,56694 | 0,8234 | 0,28706 | 0,66847 | 0,49542 |
| RPTOR | rs1468031 | 78882953 | normSNP | 0 | G/A | 61,9 | 0,116586 | 0,54794 | 1 | 0,28706 | 0,51955 | 0,60663 |
| RPTOR | rs6565488 | 78882972 | normSNP | 0 | C/A | 86,4 | 1 | 0,21792 | 0,22367 | 1 | 0,1377 | 0,21792 |
| RPTOR | rs6565489 | 78883164 | normSNP | 0 | A/C | 52,8 | 1 | 0,42775 | 0,63199 | 0,19307 | 0,52216 | 0,28307 |
| RPTOR | rs59618198 | 78883510 | normSNP | 0 | A/G | 63,1 | 0,107553 | 0,31521 | 0,8234 | 0,12995 | 0,51955 | 0,38318 |
| RPTOR | rs2333983 | 78884094 | normSNP | 0 | G/A | 84,7 | 1 | 0,23738 | 0,47784 | 0,49425 | 0,22367 | 0,23738 |
| RPTOR | rs7502267 | 78884446 | normSNP | 0 | G/C | 60,8 | 0,07203 | 0,54992 | 1 | 0,28706 | 0,5169 | 0,60071 |
| RPTOR | rs4969225 | 78884692 | normSNP | 0 | G/A | 85,2 | 1 | 0,15024 | 0,33749 | 0,49425 | 0,1377 | 0,15024 |
| RPTOR | rs2012330 | 78884941 | normSNP | 0 | A/G | 84,7 | 1 | 0,47404 | 0,23605 | 1 | 0,22367 | 0,29223 |
| RPTOR | rs4558471 | 78884949 | normSNP | 0 | A/C | 84,7 | 1 | 0,47404 | 0,23605 | 1 | 0,22367 | 0,29223 |
| RPTOR | rs7220598 | 78885690 | normSNP | 0 | G/A | 87,5 | 1 | 0,07338 | 0,07775 | 1 | 0,03961 | 0,07338 |
| RPTOR | rs7220348 | 78885858 | normSNP | 0 | C/A | 61,4 | 0,076955 | 0,50303 | 0,82058 | 0,28706 | 0,38896 | 0,72944 |
| RPTOR | rs4969301 | 78885904 | normSNP | 0 | A/G | 51,1 | 0,134297 | 0,35966 | 0,43624 | 0,17437 | 0,66449 | 0,18032 |
| RPTOR | rs4969226 | 78885940 | normSNP | 0 | A/G | 86,4 | 1 | 0,21792 | 0,22367 | 1 | 0,1377 | 0,21792 |
| RPTOR | rs7225616 | 78886782 | normSNP | 0 | C/G | 77,3 | 0,764065 | 0,77698 | 0,51329 | 0,64411 | 0,65278 | 0,47764 |
| RPTOR | rs1012117 | 78887602 | normSNP | 0 | G/A | 62,5 | 0,068563 | 0,29919 | 1 | 0,12995 | 0,38896 | 0,4824 |
| RPTOR | rs6565490 | 78888084 | normSNP | 0 | C/T | 85,8 | 0,679266 | 0,22394 | 0,46622 | 0,49425 | 0,20941 | 0,22394 |
| RPTOR | rs7212558 | 78888278 | normSNP | 0 | C/T | 54 | 0,057346 | 0,50246 | 0,80249 | 0,24091 | 0,51329 | 0,39341 |
| RPTOR | rs7225137 | 78888342 | normSNP | 0 | G/A | 54 | 0,057346 | 0,50246 | 0,80249 | 0,24091 | 0,51329 | 0,39341 |
| RPTOR | rs112205638 | 78888352 | normSNP | 0 | G/A | 77,3 | 0,764065 | 0,77698 | 0,51329 | 0,64411 | 0,65278 | 0,47764 |
| RPTOR | rs77187387 | 78888446 | normSNP | 0 | C/T | 77,3 | 0,764065 | 0,77698 | 0,51329 | 0,64411 | 0,65278 | 0,47764 |
| RPTOR | rs74733395 | 78888448 | normSNP | 0 | C/T | 77,3 | 0,764065 | 0,77698 | 0,51329 | 0,64411 | 0,65278 | 0,47764 |
| RPTOR | - | 78888489 | normSNP | 0 | A/C | 61,4 | 0,186082 | 0,09992 | 1 | 0,03774 | 0,19823 | 0,31252 |
| RPTOR | rs11657677 | 78888580 | normSNP | 0 | C/T | 63,1 | 0,49209 | 0,10851 | 0,82571 | 0,03774 | 0,28578 | 0,24838 |
| RPTOR | rs9910171 | 78889118 | normSNP | 0 | G/A | 69,3 | 0 | 0,74313 | 0,50866 | 0,45249 | 1 | 0,44873 |
| RPTOR | rs11872019 | 78889604 | normSNP | 0 | C/T | 89,8 | 1 | 0,59681 | - | - | - | - |
| RPTOR | rs9914547 | 78889678 | normSNP | 0 | G/A | 77,3 | 1 | 0,90392 | 0,66449 | 1 | 0,65754 | 0,71408 |
| RPTOR | rs2333982 | 78890238 | normSNP | 0 | C/T | 64,2 | 0,064645 | 0,46952 | 1 | 0,23023 | 0,51955 | 0,59506 |
| RPTOR | rs4969302 | 78890470 | normSNP | 0 | C/T | 85,8 | 0,679266 | 0,22394 | 0,46622 | 0,49425 | 0,20941 | 0,22394 |
| RPTOR | rs11651049 | 78890955 | normSNP | 0 | C/A | 62,5 | 0,068563 | 0,29919 | 1 | 0,12995 | 0,38896 | 0,4824 |
| RPTOR | rs11651077 | 78890975 | normSNP | 0 | C/T | 63,1 | 0,49209 | 0,32748 | 0,82571 | 0,17368 | 0,28578 | 0,62164 |
| RPTOR | rs11650768 | 78891030 | normSNP | 0 | G/C | 73,9 | 1 | 0,18427 | 1 | 0,07799 | 0,38076 | 0,49193 |
| RPTOR | - | 78891040 | normSNP | 0 | A/C | 77,8 | 0,118144 | 0,4373 | 0,37493 | 0,23023 | 0,81312 | 0,23953 |
| RPTOR | rs374653970 | 78891058 | normSNP | 0 | G/A | 60,2 | 0 | 0,67776 | 0,39295 | 0,64704 | 0,55939 | 0,474 |
| RPTOR | rs9897437 | 78891148 | normSNP | 0 | A/C | 82,4 | 0,130197 | 0,10207 | 0,34928 | 0,15362 | 0,07775 | 0,8553 |
| RPTOR | rs111261319 | 78891166 | normSNP | 0 | G/A | 79 | 1 | 0,51645 | 0,27021 | 1 | 0,25593 | 0,35563 |
| RPTOR | rs12937265 | 78891741 | normSNP | 0 | C/T | 61,4 | 0,026422 | 0,30105 | 1 | 0,12995 | 0,38541 | 0,47518 |
| RPTOR | rs9904983 | 78891753 | normSNP | 0 | G/A | 85,2 | 1 | 0,15024 | 0,33749 | 0,49425 | 0,1377 | 0,15024 |
| RPTOR | rs12938555 | 78891802 | normSNP | 0 | C/A | 84,7 | 1 | 0,23738 | 0,47784 | 0,49425 | 0,22367 | 0,23738 |
| RPTOR | rs4969303 | 78891923 | normSNP | 0 | G/A | 85,2 | 1 | 0,15024 | 0,33749 | 0,49425 | 0,1377 | 0,15024 |
| RPTOR | rs4969304 | 78892047 | normSNP | 0 | C/T | 56,2 | 0,130686 | 0,64241 | 0,63199 | 0,36558 | 0,82902 | 0,40143 |
| RPTOR | rs5016362 | 78892269 | normSNP | 0 | C/G | 51,1 | 0,000236 | 0,49234 | 0,24091 | 1 | 0,34928 | 0,43145 |
| RPTOR | rs5016361 | 78892272 | normSNP | 0 | A/G | 51,1 | 0,000005 | 0,44893 | 0,21023 | 1 | 0,32371 | 0,39196 |
| RPTOR | rs77291926 | 78892299 | normSNP | 0 | G/A | 75 | 0,005579 | 0,39344 | - | - | - | - |
| RPTOR | rs5016359 | 78892310 | normSNP | 0 | C/T | 80,7 | 0,729955 | 0,01219 | 0,17614 | 0,11643 | 0,0183 | 0,01219 |
| RPTOR | rs9903928 | 78892323 | normSNP | 0 | G/C | 80,1 | 0,738348 | 0,19001 | 0,26372 | 0,29556 | 0,10415 | 0,57763 |
| RPTOR | rs9903929 | 78892326 | normSNP | 0 | G/A | 80,1 | 0,738348 | 0,19001 | 0,26372 | 0,29556 | 0,10415 | 0,57763 |
| RPTOR | rs9912386 | 78892337 | normSNP | 0 | T/C | 52,8 | 0,085646 | 0,95811 | 1 | 0,77674 | 0,82757 | 0,86522 |
| RPTOR | rs75869656 | 78892348 | normSNP | 0 | G/A | 51,1 | 0,134297 | 0,71828 | 0,79553 | 0,41692 | 0,66449 | 0,50427 |
| RPTOR | rs12944674 | 78892569 | normSNP | 0 | G/A | 62,5 | 0,022287 | 0,43371 | 0,82058 | 0,23023 | 0,38541 | 0,71716 |
| RPTOR | rs34048269 | 78892647 | normSNP | 0 | G/A | 80,1 | 0,505387 | 0,41486 | 0,82571 | 0,49425 | 0,50293 | 0,41486 |
| RPTOR | rs12451162 | 78893276 | normSNP | 0 | G/A | 77,8 | 1 | 0,36306 | 0,51329 | 0,29556 | 0,26372 | 0,85444 |
| RPTOR | rs4969305 | 78893513 | normSNP | 0 | G/A | 85,2 | 1 | 0,15024 | 0,33749 | 0,49425 | 0,1377 | 0,15024 |
| RPTOR | rs7215994 | 78893671 | normSNP | 0 | A/G | 64,8 | 0,484636 | 0,20202 | 0,2755 | 0,28706 | 0,0872 | 0,73993 |
| RPTOR | rs12938300 | 78894022 | normSNP | 0 | G/C | 60,8 | 0,024135 | 0,2722 | 0,81718 | 0,12995 | 0,2755 | 0,58914 |
| RPTOR | rs9893657 | 78894321 | normSNP | 0 | C/T | 77,8 | 1 | 0,36306 | 0,51329 | 0,29556 | 0,26372 | 0,85444 |
| RPTOR | rs9900417 | 78894338 | normSNP | 0 | T/C | 76,7 | 0,772392 | 0,51191 | 0,82902 | 0,29556 | 0,50866 | 0,85488 |
| RPTOR | rs9897968 | 78894514 | normSNP | 0 | G/A | 76,1 | 0,769863 | 0,55872 | 1 | 0,29556 | 0,66142 | 0,7147 |
| RPTOR | rs34517247 | 78894793 | normSNP | 0 | A/G | 60,2 | 0,044455 | 0,15939 | 0,81718 | 0,07105 | 0,19248 | 0,48115 |
| RPTOR | rs7503237 | 78894845 | normSNP | 0 | A/G | 63,6 | 0,821795 | 0,43092 | 0,38541 | 0,5336 | 0,19823 | 0,75604 |
| RPTOR | rs4969306 | 78894938 | normSNP | 0 | T/C | 76,1 | 0,769863 | 0,673 | 0,38896 | 1 | 0,38076 | 0,46428 |
| RPTOR | rs9901846 | 78895153 | normSNP | 0 | G/A | 63,1 | 0,650649 | 0,55846 | 0,38541 | 0,76375 | 0,28274 | 0,64714 |
| RPTOR | rs59809183 | 78895301 | normSNP | 0 | G/T | 60,2 | 0,044455 | 0,15939 | 0,81718 | 0,07105 | 0,19248 | 0,48115 |
| RPTOR | rs12452516 | 78895378 | normSNP | 0 | G/C | 77,8 | 1 | 0,36306 | 0,51329 | 0,29556 | 0,26372 | 0,85444 |
| RPTOR | rs9902459 | 78895390 | normSNP | 0 | C/A | 63,1 | 0,650649 | 0,55846 | 0,38541 | 0,76375 | 0,28274 | 0,64714 |
| RPTOR | rs9908454 | 78895460 | normSNP | 0 | T/G | 76,7 | 0,772392 | 0,51191 | 0,82902 | 0,29556 | 0,50866 | 0,85488 |
| RPTOR | rs11653064 | 78895735 | normSNP | 0 | A/G | 58,5 | 0,030329 | 0,33992 | 0,81312 | 0,17368 | 0,2755 | 0,59816 |
| RPTOR | rs11657655 | 78895788 | normSNP | 0 | C/T | 60,2 | 0,044455 | 0,15939 | 0,81718 | 0,07105 | 0,19248 | 0,48115 |
| RPTOR | rs9908270 | 78895889 | normSNP | 0 | A/C | 62,5 | 0,820641 | 0,69242 | 0,51329 | 0,76375 | 0,39146 | 0,75896 |
| RPTOR | rs9909367 | 78896010 | normSNP | 0 | A/G | 75,6 | 1 | 0,33278 | 1 | 0,15362 | 0,50866 | 0,59555 |
| RPTOR | rs2271602 | 78896488 | normSNP | 0 | C/T | 60,2 | 0,044455 | 0,15939 | 0,81718 | 0,07105 | 0,19248 | 0,48115 |
| RPTOR | rs2271603 | 78896529 | normSNP | 0 | C/T | 77,3 | 1 | 0,66721 | 0,38541 | 1 | 0,37493 | 0,46322 |
| RPTOR | rs2271605 | 78896762 | normSNP | 0 | C/T | 62,5 | 0,068563 | 0,22899 | 0,65278 | 0,12995 | 0,19585 | 0,72565 |
| RPTOR | rs2271606 | 78896768 | normSNP | 0 | C/T | 86,4 | 0,654989 | 0,32302 | 0,62227 | 0,49425 | 0,30762 | 0,32302 |
| RPTOR | rs3817292 | 78897056 | normSNP | 0 | C/G | 60,2 | 0,044455 | 0,15939 | 0,81718 | 0,07105 | 0,19248 | 0,48115 |
| RPTOR | rs2271607 | 78897146 | normSNP | 0 | T/C | 76,7 | 0,772392 | 0,51191 | 0,82902 | 0,29556 | 0,50866 | 0,85488 |
| RPTOR | rs7217786 | 78897547 | normSNP | 0 | C/T | 77,3 | 0,764065 | 0,54643 | 0,2755 | 0,64411 | 0,36782 | 0,28596 |
| RPTOR | rs6565491 | 78897561 | normSNP | 0 | T/C | 85,8 | 0,679266 | 0,22394 | 0,46622 | 0,49425 | 0,20941 | 0,22394 |
| RPTOR | rs6565492 | 78897587 | normSNP | 0 | G/A | 85,8 | 0,679266 | 0,22394 | 0,46622 | 0,49425 | 0,20941 | 0,22394 |
| RPTOR | rs6565493 | 78897674 | normSNP | 0 | T/C | 85,8 | 0,679266 | 0,22394 | 0,46622 | 0,49425 | 0,20941 | 0,22394 |
| RPTOR | rs201713580 | 78897732 | normSNP | 0 | C/T | 60,2 | 0,044455 | 0,15939 | 0,81718 | 0,07105 | 0,19248 | 0,48115 |
| RPTOR | rs72859699 | 78897733 | normSNP | 0 | T/A | 60,2 | 0,044455 | 0,15939 | 0,81718 | 0,07105 | 0,19248 | 0,48115 |
| RPTOR | rs908236 | 78898600 | normSNP | 0 | G/C | 63,1 | 0,49209 | 0,72863 | 0,82571 | 0,50044 | 0,52216 | 0,86939 |
| RPTOR | rs6565494 | 78898778 | normSNP | 0 | C/T | 85,8 | 0,679266 | 0,22394 | 0,46622 | 0,49425 | 0,20941 | 0,22394 |
| RPTOR | rs6565495 | 78898788 | normSNP | 0 | T/C | 85,8 | 0,679266 | 0,22394 | 0,46622 | 0,49425 | 0,20941 | 0,22394 |
| RPTOR | rs2271608 | 78899458 | normSNP | 0 | G/A | 77,3 | 1 | 0,4438 | 0,66449 | 0,29556 | 0,37493 | 1 |
| RPTOR | rs3817293 | 78899595 | normSNP | 0 | G/A | 76,7 | 0,772392 | 0,51191 | 0,82902 | 0,29556 | 0,50866 | 0,85488 |
| RPTOR | rs7502001 | 78900118 | normSNP | 0 | T/G | 85,8 | 0,679266 | 0,22394 | 0,46622 | 0,49425 | 0,20941 | 0,22394 |
| RPTOR | rs4969227 | 78900598 | normSNP | 0 | C/T | 76,7 | 0,772392 | 0,79865 | 0,5169 | 1 | 0,50866 | 0,58295 |
| RPTOR | rs4969228 | 78900811 | normSNP | 0 | A/G | 77,3 | 1 | 0,90392 | 0,66449 | 1 | 0,65754 | 0,71408 |
| RPTOR | - | 78901406 | normSNP | 0 | A/C | 69,3 | 1 | 0,21629 | 0,19965 | 0,45614 | 0,08417 | 0,50961 |
| RPTOR | - | 78901408 | normSNP | 0 | A/C | 84,7 | 0,414695 | 0,79854 | 0,63199 | 0,55323 | 0,80249 | 0,54562 |
| RPTOR | rs4969307 | 78901410 | normSNP | 0 | G/C | 59,7 | 0,82493 | 0,95547 | 1 | 0,77674 | 0,83078 | 0,87978 |
| RPTOR | rs36171362 | 78901443 | normSNP | 0 | T/G | 60,2 | 0,044455 | 0,15939 | 0,81718 | 0,07105 | 0,19248 | 0,48115 |
| RPTOR | rs112226528 | 78901532 | normSNP | 0 | A/C | 83,5 | 0,236986 | 0,09056 | 0,81312 | 0,11643 | 0,20941 | 0,09056 |
| RPTOR | rs10625103 | 78901539 | normSNP | 0 | A/C | 63,6 | 0 | 1 | - | - | - | - |
| RPTOR | rs35087326 | 78901540 | normSNP | 0 | A/C | 70,5 | 0,076431 | 0,32176 | 0,39146 | 0,29556 | 0,20012 | 0,71156 |
| RPTOR | rs56072626 | 78901669 | normSNP | 0 | G/A | 77,8 | 1 | 0,79426 | 0,51329 | 1 | 0,50293 | 0,58178 |
| RPTOR | rs7208237 | 78901827 | normSNP | 0 | C | 100 | - | - | - | - | - | - |
| RPTOR | rs4969310 | 78901980 | normSNP | 0 | C/T | 87,5 | 1 | 0,446 | 0,45249 | 1 | 0,30762 | 0,446 |
| RPTOR | - | 78903259 | normSNP | 0 | A/C | 87,5 | 0,615347 | 0,30567 | 0,61069 | 0,49425 | 0,28883 | 0,30567 |
| RPTOR | rs68120541 | 78903681 | normSNP | 0 | T/C | 59,7 | 0,026775 | 0,18138 | 1 | 0,07105 | 0,2755 | 0,37371 |
| RPTOR | rs142247229 | 78903833 | normSNP | 0 | A/C | 80,7 | 0,108677 | 0,38076 | - | - | - | - |
| RPTOR | rs62636946 | 78904091 | normSNP | 0 | T/C | 61,9 | 0 | 0,45249 | - | - | - | - |
| RPTOR | rs575696923 | 78904112 | normSNP | 0 | A/C | 83,5 | 0,454388 | 0,82058 | - | - | - | - |
| RPTOR | - | 78904247 | normSNP | 0 | A/C | 61,4 | 0 | 0,61069 | - | - | - | - |
| RPTOR | rs62068527 | 78904311 | normSNP | 0 | T/C | 77,3 | 1 | 0,4438 | 0,66449 | 0,29556 | 0,37493 | 1 |
| RPTOR | rs62068528 | 78904648 | normSNP | 0 | C/T | 77,8 | 1 | 0,36306 | 0,51329 | 0,29556 | 0,26372 | 0,85444 |
| RPTOR | rs9899051 | 78904680 | normSNP | 0 | A/G | 54 | 0,019913 | 0,6322 | 0,61069 | 0,36558 | 0,82571 | 0,3791 |
| RPTOR | rs35848637 | 78904962 | normSNP | 0 | G/A | 77,8 | 1 | 0,36306 | 0,51329 | 0,29556 | 0,26372 | 0,85444 |
| RPTOR | rs9899850 | 78905362 | normSNP | 0 | C/A | 76,7 | 1 | 0,24483 | 0,66449 | 0,15362 | 0,26372 | 0,85945 |
| RPTOR | rs4969311 | 78906360 | normSNP | 0 | C/G | 52,8 | 0,031387 | 0,43762 | 0,43624 | 0,24091 | 0,82571 | 0,21925 |
| RPTOR | rs2090202 | 78906844 | normSNP | 0 | C/A | 76,7 | 1 | 0,24483 | 0,66449 | 0,15362 | 0,26372 | 0,85945 |
| RPTOR | rs2090203 | 78906976 | normSNP | 0 | A/G | 76,7 | 0,772392 | 0,53486 | 0,27966 | 1 | 0,27021 | 0,35947 |
| RPTOR | rs9902338 | 78907309 | normSNP | 0 | A/G | 77,3 | 1 | 0,4438 | 0,66449 | 0,29556 | 0,37493 | 1 |
| RPTOR | rs35071447 | 78907809 | normSNP | 0 | C/A | 59,7 | 0,026775 | 0,18138 | 1 | 0,07105 | 0,2755 | 0,37371 |
| RPTOR | rs73357804 | 78908258 | normSNP | 0 | G/A | 76,1 | 1 | 0,77763 | 0,5169 | 0,64411 | 0,65754 | 0,47861 |
| RPTOR | rs62068529 | 78908322 | normSNP | 0 | C/T | 76,7 | 1 | 0,51702 | 0,38541 | 0,64411 | 0,26372 | 0,59502 |
| RPTOR | rs9915450 | 78909278 | normSNP | 0 | G/A | 77,3 | 1 | 0,4438 | 0,66449 | 0,29556 | 0,37493 | 1 |
| RPTOR | rs117176153 | 78909803 | normSNP | 0 | A/G | 88,6 | 0,296329 | 0,24404 | 0,11004 | 1 | 0,09388 | 0,16918 |
| RPTOR | rs12602601 | 78910439 | normSNP | 0 | G/T | 77,3 | 1 | 0,66721 | 0,38541 | 1 | 0,37493 | 0,46322 |
| RPTOR | rs12602731 | 78910440 | normSNP | 0 | C/T | 77,3 | 1 | 0,66721 | 0,38541 | 1 | 0,37493 | 0,46322 |
| RPTOR | rs35635776 | 78910499 | normSNP | 0 | C/A | 59,7 | 0,077289 | 0,33728 | 0,81718 | 0,17368 | 0,27966 | 0,60517 |
| RPTOR | rs11653325 | 78910629 | normSNP | 0 | C/T | 91,5 | 0,569827 | 0,39373 | - | - | - | - |
| RPTOR | rs2333885 | 78910748 | normSNP | 0 | T/C | 59,1 | 0,048985 | 0,28744 | 0,64017 | 0,17368 | 0,19248 | 0,72801 |
| RPTOR | rs12936701 | 78910751 | normSNP | 0 | A/C | 91,5 | 0,569827 | 0,39373 | - | - | - | - |
| RPTOR | rs7501689 | 78910800 | normSNP | 0 | C/T | 91,5 | 0,569827 | 0,39373 | - | - | - | - |
| RPTOR | rs1877926 | 78911041 | normSNP | 0 | C/T | 60,2 | 0,044455 | 0,50549 | 0,81718 | 0,28706 | 0,38541 | 0,7249 |
| RPTOR | rs7210742 | 78912010 | normSNP | 0 | G/A | 63,1 | 1 | 0,54782 | 0,51329 | 0,5336 | 0,28477 | 0,87587 |
| RPTOR | rs7209116 | 78912121 | normSNP | 0 | A/G | 61,9 | 1 | 0,55991 | 0,38076 | 0,76375 | 0,28477 | 0,64324 |
| RPTOR | rs9911223 | 78912271 | normSNP | 0 | G/A | 76,1 | 0,769863 | 0,4094 | 0,19585 | 1 | 0,1881 | 0,27153 |
| RPTOR | rs7219318 | 78912354 | normSNP | 0 | C/T | 62,5 | 0,820641 | 0,42803 | 0,2755 | 0,76375 | 0,19823 | 0,53919 |
| RPTOR | rs60278804 | 78912389 | normSNP | 0 | G/A | 76,7 | 0,772392 | 0,51191 | 0,82902 | 0,29556 | 0,50866 | 0,85488 |
| RPTOR | rs6565496 | 78912599 | normSNP | 0 | G/A | 86,9 | 1 | 0,31725 | 0,32371 | 1 | 0,20941 | 0,31725 |
| RPTOR | rs6565497 | 78912633 | normSNP | 0 | T/A | 62,5 | 1 | 0,4346 | 0,38076 | 0,5336 | 0,19965 | 0,75327 |
| RPTOR | rs6565498 | 78912982 | normSNP | 0 | C/T | 86,9 | 1 | 0,31725 | 0,32371 | 1 | 0,20941 | 0,31725 |
| RPTOR | rs6565499 | 78913066 | normSNP | 0 | G/A | 87,5 | 1 | 0,446 | 0,45249 | 1 | 0,30762 | 0,446 |
| RPTOR | rs7224976 | 78913441 | normSNP | 0 | C/T | 86,9 | 1 | 0,31725 | 0,32371 | 1 | 0,20941 | 0,31725 |
| RPTOR | rs34597387 | 78913787 | normSNP | 0 | C/T | 76,1 | 1 | 0,294 | 0,82902 | 0,15362 | 0,37493 | 0,7234 |
| RPTOR | rs528496779 | 78913987 | normSNP | 0 | A/C | 79,5 | 0,001184 | 0,19088 | 0,48769 | 0,07105 | 0,59681 | 0,19927 |
| RPTOR | rs17848659 | 78914245 | normSNP | 0 | G/T | 60,2 | 0,044455 | 0,15939 | 0,81718 | 0,07105 | 0,19248 | 0,48115 |
| RPTOR | rs2271609 | 78914545 | normSNP | 0 | A/G | 50 | 0,018339 | 1 | 1 | 1 | 1 | 1 |
| RPTOR | rs6420480 | 78914748 | normSNP | 0 | A/G | 86,9 | 0,633425 | 0,35913 | 0,80249 | 0,49425 | 0,43624 | 0,35913 |
| RPTOR | rs908237 | 78914751 | normSNP | 0 | G/A | 76,7 | 0,772392 | 0,79865 | 0,5169 | 1 | 0,50866 | 0,58295 |
| RPTOR | rs17848664 | 78914761 | normSNP | 0 | A/C | 77,3 | 1 | 0,90392 | 0,66449 | 1 | 0,65754 | 0,71408 |
| RPTOR | rs7501577 | 78915130 | normSNP | 0 | G/A | 86,4 | 0,654989 | 0,32302 | 0,62227 | 0,49425 | 0,30762 | 0,32302 |
| RPTOR | rs1468032 | 78915387 | normSNP | 0 | A/T | 58 | 0,078597 | 0,55526 | 0,81312 | 0,33085 | 0,38541 | 0,73066 |
| RPTOR | rs9915393 | 78915828 | normSNP | 0 | C/G | 71 | 0,437733 | 0,15705 | 1 | 0,07105 | 0,27021 | 0,42528 |
| RPTOR | rs2292639 | 78915955 | normSNP | 0 | A/C | 64,8 | 0,815986 | 0,90193 | 0,66449 | 1 | 0,66944 | 0,74657 |
| RPTOR | rs62068531 | 78915978 | normSNP | 0 | G/T | 76,1 | 1 | 0,55056 | 0,27966 | 0,64411 | 0,37493 | 0,28708 |
| RPTOR | rs11654011 | 78916128 | normSNP | 0 | G/T | 60,8 | 0,07203 | 0,55378 | 0,64704 | 0,28706 | 0,82902 | 0,3825 |
| RPTOR | rs34449704 | 78916379 | normSNP | 0 | G/A | 60,2 | 0,044455 | 0,5674 | 0,81718 | 0,28706 | 0,66449 | 0,48115 |
| RPTOR | rs6565500 | 78916489 | normSNP | 0 | A/G | 64,8 | 0,484636 | 0,69318 | 0,51329 | 0,72474 | 0,39344 | 0,73993 |
| RPTOR | rs8075155 | 78916748 | normSNP | 0 | G | 100 | - | - | - | - | - | - |
| RPTOR | rs2878052 | 78916910 | normSNP | 0 | G/A | 74,4 | 0,785467 | 0,25569 | 0,66847 | 0,15362 | 0,2755 | 0,85965 |
| RPTOR | rs1468033 | 78916953 | normSNP | 0 | A/G | 65,3 | 1 | 0,79326 | 0,5169 | 1 | 0,5213 | 0,62954 |
| RPTOR | rs999343 | 78917023 | normSNP | 0 | C/T | 65,3 | 1 | 0,79326 | 0,5169 | 1 | 0,5213 | 0,62954 |
| RPTOR | rs4508478 | 78917317 | normSNP | 0 | G/A | 65,3 | 1 | 0,79326 | 0,5169 | 1 | 0,5213 | 0,62954 |
| RPTOR | rs62068550 | 78917533 | normSNP | 0 | G/A | 86,9 | 1 | 0,31725 | 0,32371 | 1 | 0,20941 | 0,31725 |
| RPTOR | rs4999609 | 78917730 | normSNP | 0 | G/A | 65,3 | 1 | 0,79326 | 0,5169 | 1 | 0,5213 | 0,62954 |
| RPTOR | rs34056644 | 78918607 | normSNP | 0 | A/T | 77,8 | 1 | 0,08771 | 0,82757 | 0,11643 | 0,26372 | 0,08771 |
| RPTOR | rs72861344 | 78918966 | normSNP | 0 | G/A | 75 | 1 | 0,20437 | 0,51955 | 0,15362 | 0,1881 | 1 |
| RPTOR | rs17848711 | 78919131 | normSNP | 0 | G/A | 77,8 | 1 | 0,52666 | 0,2755 | 1 | 0,26372 | 0,35793 |
| RPTOR | rs8075387 | 78919162 | normSNP | 0 | G/A | 59,7 | 0,186163 | 0,60277 | 1 | 0,33085 | 0,51955 | 0,61548 |
| RPTOR | rs8077224 | 78919285 | normSNP | 0 | A/G | 51,7 | 0,000001 | 0,70911 | 0,51329 | 1 | 0,45249 | 0,71373 |
| RPTOR | rs8080910 | 78919349 | normSNP | 0 | G/A | 64,2 | 0,245052 | 0,53945 | 0,51955 | 0,55939 | 0,2755 | 0,88265 |
| RPTOR | rs17848707 | 78919387 | normSNP | 0 | G/A | 58,5 | 0,084069 | 0,60585 | 0,64017 | 0,33085 | 0,82902 | 0,39344 |
| RPTOR | rs1567962 | 78919558 | normSNP | 0 | C/T | 59,1 | 0,048985 | 0,75703 | 0,64017 | 0,50044 | 1 | 0,48633 |
| RPTOR | rs2138119 | 78919837 | normSNP | 0 | G/C | 59,7 | 0,186163 | 0,9458 | 1 | 0,74706 | 0,83008 | 0,8671 |
| RPTOR | rs2271610 | 78919859 | normSNP | 0 | C/G | 87,5 | 0,615347 | 0,30567 | 0,61069 | 0,49425 | 0,28883 | 0,30567 |
| RPTOR | rs2271611 | 78919892 | normSNP | 0 | G/A | 66,5 | 1 | 0,79374 | 0,51955 | 1 | 0,51955 | 0,63271 |
| RPTOR | rs2271612 | 78919920 | normSNP | 0 | C/T | 52,3 | 0,133442 | 0,5252 | 0,61069 | 0,26682 | 0,66449 | 0,31495 |
| RPTOR | rs7503779 | 78920172 | normSNP | 0 | G/A | 59,1 | 0,048985 | 0,75703 | 0,64017 | 0,50044 | 1 | 0,48633 |
| RPTOR | rs7208853 | 78920340 | normSNP | 0 | G/C | 74,4 | 1 | 0,10071 | 0,51955 | 0,07799 | 0,12215 | 0,8638 |
| RPTOR | rs35612742 | 78920628 | normSNP | 0 | A/C | 75 | 1 | 0,20437 | 0,51955 | 0,15362 | 0,1881 | 1 |
| RPTOR | rs7209380 | 78920681 | normSNP | 0 | G/A | 88,1 | 1 | 0,60518 | 0,61069 | 1 | 0,43624 | 0,60518 |
| RPTOR | rs9913906 | 78920807 | normSNP | 0 | A/G | 87,5 | 1 | 0,79973 | 0,80249 | 1 | 0,61069 | 0,79973 |
| RPTOR | rs4969231 | 78921117 | normSNP | 0 | C/T | 72,2 | 0,794069 | 0,13375 | 0,05409 | 1 | 0,05101 | 0,11905 |
| RPTOR | rs9912373 | 78921211 | normSNP | 0 | G/A | 89,2 | 1 | 0,43624 | - | - | - | - |
| RPTOR | rs7502563 | 78921793 | normSNP | 0 | G/A | 55,7 | 0,031411 | 0,3516 | 0,32371 | 0,21023 | 1 | 0,16081 |
| RPTOR | rs4969313 | 78921997 | normSNP | 0 | G/A | 55,7 | 0,031411 | 0,3516 | 0,32371 | 0,21023 | 1 | 0,16081 |
| RPTOR | rs1468034 | 78922158 | normSNP | 0 | G/T | 54 | 0,005809 | 0,39605 | 0,43624 | 0,21023 | 0,8234 | 0,20256 |
| RPTOR | rs2138123 | 78922219 | normSNP | 0 | T/C | 55,1 | 0,018521 | 0,40506 | 0,45249 | 0,21023 | 0,82571 | 0,21469 |
| RPTOR | rs2138124 | 78922477 | normSNP | 0 | C/T | 55,7 | 0,031411 | 0,3516 | 0,32371 | 0,21023 | 1 | 0,16081 |
| RPTOR | rs8082382 | 78922555 | normSNP | 0 | T/C | 53,4 | 0,132201 | 0,59628 | 0,45249 | 0,39373 | 1 | 0,3134 |
| RPTOR | rs8082395 | 78922584 | normSNP | 0 | T/G | 54,5 | 0,033525 | 0,66355 | 0,80249 | 0,36558 | 0,66142 | 0,48751 |
| RPTOR | rs8078643 | 78922683 | normSNP | 0 | A/G | 52,8 | 0,031387 | 0,43762 | 0,43624 | 0,24091 | 0,82571 | 0,21925 |
| RPTOR | rs6565501 | 78922914 | normSNP | 0 | C/T | 55,7 | 0,031411 | 0,3516 | 0,32371 | 0,21023 | 1 | 0,16081 |
| RPTOR | rs7222041 | 78922952 | normSNP | 0 | C/G | 52,3 | 0,133442 | 0,5252 | 0,61069 | 0,26682 | 0,66449 | 0,31495 |
| RPTOR | rs4969314 | 78923007 | normSNP | 0 | T/G | 72,7 | 1 | 0,08184 | 0,03206 | 1 | 0,0293 | 0,08331 |
| RPTOR | rs3751940 | 78923178 | normSNP | 0 | C/T | 55,7 | 0,031411 | 0,3516 | 0,32371 | 0,21023 | 1 | 0,16081 |
| RPTOR | rs1567963 | 78923828 | normSNP | 0 | T/C | 55,7 | 0,0858 | 0,57955 | 0,46622 | 0,36558 | 1 | 0,30837 |
| RPTOR | rs1468035 | 78923953 | normSNP | 0 | A/G | 52,8 | 0,009656 | 0,62537 | 0,59681 | 0,36558 | 0,8234 | 0,36591 |
| RPTOR | rs6565502 | 78924478 | normSNP | 0 | A/G | 56,8 | 0,081949 | 0,44866 | 0,63199 | 0,21023 | 0,66449 | 0,30485 |
| RPTOR | rs7218122 | 78924882 | normSNP | 0 | T/C | 56,2 | 0,05156 | 0,41261 | 0,46622 | 0,21023 | 0,82757 | 0,22572 |
| RPTOR | rs60311504 | 78925257 | normSNP | 0 | T/C | 53,4 | 0,017607 | 0,55592 | 0,43624 | 0,36558 | 1 | 0,2843 |
| RPTOR | rs4969315 | 78925383 | normSNP | 0 | A/G | 55,7 | 0,031411 | 0,3516 | 0,32371 | 0,21023 | 1 | 0,16081 |
| RPTOR | rs6420481 | 78925950 | normSNP | 0 | T/C | 53,4 | 0,017607 | 0,55592 | 0,43624 | 0,36558 | 1 | 0,2843 |
| RPTOR | rs9890953 | 78926821 | normSNP | 0 | C/T | 65,3 | 0,487128 | 0,74344 | 0,51955 | 0,5336 | 0,82902 | 0,44372 |
| RPTOR | rs9897841 | 78926853 | normSNP | 0 | C/T | 50 | 0,398577 | 0,58554 | 0,33749 | 1 | 0,39146 | 0,56348 |
| RPTOR | rs9891673 | 78927094 | normSNP | 0 | C/T | 54,5 | 0,033525 | 0,56875 | 0,45249 | 0,36558 | 1 | 0,29696 |
| RPTOR | rs55833830 | 78927342 | normSNP | 0 | C/A | 81,2 | 0,171764 | 0,22685 | 0,16844 | 0,64411 | 0,08749 | 0,36487 |
| RPTOR | rs72532144 | 78928309 | normSNP | 0 | A/C | 97,7 | 0,033801 | 0,49425 | - | - | - | - |
| RPTOR | rs7225525 | 78928792 | normSNP | 0 | C/T | 55,1 | 0,018521 | 0,40506 | 0,45249 | 0,21023 | 0,82571 | 0,21469 |
| RPTOR | rs7224748 | 78929020 | normSNP | 0 | G/A | 55,1 | 0,018521 | 0,40506 | 0,45249 | 0,21023 | 0,82571 | 0,21469 |
| RPTOR | rs8068193 | 78930171 | normSNP | 0 | G/A | 54,5 | 0,033525 | 0,56875 | 0,45249 | 0,36558 | 1 | 0,29696 |
| RPTOR | rs12951778 | 78930344 | normSNP | 0 | T/C | 75,6 | 0,018445 | 0,19953 | 0,19965 | 1 | 0,13384 | 0,19953 |
| RPTOR | rs58504028 | 78932006 | normSNP | 0 | A/C | 85,2 | 0 | 0,52084 | 0,26682 | 0,50044 | 0,39341 | 0,33013 |
| RPTOR | rs6565503 | 78933424 | normSNP | 0 | G/A | 98,9 | 0 | 1 | - | - | - | - |
| RPTOR | rs9897319 | 78935071 | normSNP | 0 | G/A | 67,6 | 0,148966 | 0,16439 | 0,27966 | 0,07799 | 0,83112 | 0,11005 |
| RPTOR | rs9899178 | 78935197 | normSNP | 0 | C/T | 64,8 | 0,101146 | 0,09428 | 0,27021 | 0,03876 | 1 | 0,07647 |
| RPTOR | rs34781048 | 78937165 | normSNP | 0 | G/A | 56,8 | 0,192749 | 0,66411 | 0,81312 | 0,36558 | 0,66683 | 0,5067 |
| RPTOR | rs71370203 | 78937249 | normSNP | 0 | C/G | 54 | 0 | 0,0965 | 0,05409 | 0,03058 | 0,64411 | 0,03554 |
| RPTOR | rs3751938 | 78937925 | normSNP | 0 | T/C | 65,3 | 0,03691 | 0,2291 | 0,08686 | 0,39373 | 0,26372 | 0,1133 |
| RPTOR | rs3751937 | 78938011 | normSNP | 0 | C/A | 80,7 | 0,108677 | 0,66142 | - | - | - | - |
| RPTOR | rs3751936 | 78938204 | normSNP | 0 | G/C | 79 | 0,339731 | 0,28139 | 0,51329 | 0,49425 | 0,27021 | 0,28139 |
| RPTOR | rs11547302 | 78938385 | normSNP | 0 | G/A | 82,4 | 1 | 0,25919 | 0,49599 | 0,49425 | 0,24675 | 0,25919 |
| RPTOR | rs3751934 | 78938498 | normSNP | 0 | C/A | 56,2 | 0,829508 | 0,08758 | 0,03613 | 1 | 0,05409 | 0,16137 |
| RPTOR | rs3751932 | 78939414 | normSNP | 0 | T/C | 89,2 | 1 | 0,28002 | 0,28883 | 1 | 0,17437 | 0,28002 |
| RPTOR | rs1062935 | 78939857 | normSNP | 0 | T/C | 53,4 | 0,017607 | 0,93723 | 0,79553 | 0,76375 | 1 | 0,72188 |
| RPTOR | - | 78939917 | normSNP | 0 | A/C | 61,4 | 0,498878 | 0,2908 | 0,11706 | 0,74706 | 0,19965 | 0,18951 |
| RPTOR | rs1045626 | 78939964 | normSNP | 0 | C/T | 54,5 | 0,010558 | 0,7652 | 0,61069 | 0,5336 | 1 | 0,47384 |
| RPTOR | rs4969318 | 78940215 | normSNP | 0 | G/T | 77,3 | 0,764065 | 0,19321 | 0,12636 | 0,64411 | 0,07072 | 0,28596 |
| RPTOR | rs7221717 | 78940347 | normSNP | 0 | A/G | 75,6 | 1 | 0,22503 | 0,08417 | 0,64411 | 0,12215 | 0,10924 |
| RPTOR | rs7223311 | 78940614 | normSNP | 0 | C/G | 54,5 | 0,033525 | 0,66355 | 0,80249 | 0,36558 | 0,66142 | 0,48751 |
| RPTOR | rs6565504 | 78940813 | normSNP | 0 | G/A | 76,7 | 0,772392 | 0,29229 | 0,12969 | 1 | 0,12215 | 0,1986 |
| RPTOR | rs6565505 | 78940874 | normSNP | 0 | T/C | 71 | 1 | 0,23011 | 0,0872 | 0,69315 | 0,12969 | 0,12857 |
| RPTOR | rs6565506 | 78941160 | normSNP | 0 | G/C | 75,6 | 1 | 0,22503 | 0,08417 | 0,64411 | 0,12215 | 0,10924 |
| RPTOR | rs62068555 | 78941544 | normSNP | 0 | C/G | 77,8 | 1 | 0,28343 | 0,12636 | 1 | 0,11706 | 0,1971 |
| RPTOR | - | 78941648 | normSNP | 0 | A/C | 76,1 | 0,137642 | 0,68538 | 0,39146 | 1 | 0,38896 | 0,4318 |
| RPTOR | rs4969319 | 78942230 | normSNP | 0 | C/T | 72,7 | 1 | 0,2028 | 0,08686 | 1 | 0,08182 | 0,16701 |
| RPTOR | rs11650042 | 78942635 | normSNP | 0 | C/T | 75 | 0,083432 | 0,42993 | 0,19965 | 1 | 0,19823 | 0,23808 |
| RPTOR | rs112148409 | 78942655 | normSNP | 0 | A/C | 61,4 | 0,000595 | 0,06148 | 0,13384 | 0,45249 | 0,0183 | 0,59754 |
| RPTOR | rs9898301 | 78942679 | normSNP | 0 | C/T | 76,1 | 1 | 0,31713 | 0,12969 | 0,64411 | 0,18267 | 0,15483 |
| RPTOR | rs9890297 | 78942916 | normSNP | 0 | A/G | 75,6 | 1 | 0,43094 | 0,19585 | 0,64411 | 0,27021 | 0,21405 |
| RPTOR | rs7406130 | 78943074 | normSNP | 0 | T/C | 70,5 | 0,454607 | 0,21054 | 0,08686 | 0,39341 | 0,19823 | 0,0809 |
| RPTOR | rs9893284 | 78943109 | normSNP | 0 | G/A | 76,1 | 1 | 0,31713 | 0,12969 | 0,64411 | 0,18267 | 0,15483 |
| RPTOR | rs9899212 | 78943114 | normSNP | 0 | C/T | 76,1 | 1 | 0,31713 | 0,12969 | 0,64411 | 0,18267 | 0,15483 |
| RPTOR | rs11649948 | 78943118 | normSNP | 0 | G/T | 75,6 | 0,084073 | 0,55552 | 0,28477 | 1 | 0,28274 | 0,32579 |
| RPTOR | rs11650349 | 78943270 | normSNP | 0 | C/T | 80,1 | 1 | 0,82932 | 1 | 0,55323 | 0,82058 | 0,8476 |
| RPTOR | rs7220687 | 78943288 | normSNP | 0 | T/C | 72,7 | 0,590502 | 0,77615 | 0,52216 | 0,64411 | 0,66683 | 0,47774 |
| RPTOR | rs4969320 | 78943907 | normSNP | 0 | C/T | 79 | 0,339731 | 0,01758 | 0,04897 | 0,49425 | 0,01469 | 0,01758 |
| RPTOR | rs4969321 | 78943920 | normSNP | 0 | T/C | 79 | 0,339731 | 0,01758 | 0,04897 | 0,49425 | 0,01469 | 0,01758 |
| RPTOR | rs4969322 | 78944104 | normSNP | 0 | C/G | 54,5 | 0,033525 | 0,74784 | 0,45249 | 0,76375 | 0,66142 | 0,48751 |
| RPTOR | rs113335931 | 78944738 | normSNP | 0 | A/G | 86,9 | 0,633425 | 0,42637 | 0,20941 | 1 | 0,19307 | 0,27246 |
| RPTOR | rs6565507 | 78944840 | normSNP | 0 | C/T | 75,6 | 1 | 0,22503 | 0,08417 | 0,64411 | 0,12215 | 0,10924 |
| RPTOR | rs113437382 | 78945137 | normSNP | 0 | C/T | 76,1 | 1 | 0,31713 | 0,12969 | 0,64411 | 0,18267 | 0,15483 |
| RPTOR | rs873445 | 78945566 | normSNP | 0 | C/T | 63,1 | 0,000063 | 0,10074 | 0,03225 | 0,20941 | 0,22367 | 0,04939 |
| RPTOR | rs11653888 | 78945935 | normSNP | 0 | G/T | 75,6 | 0,084073 | 0,31511 | 0,13384 | 1 | 0,13218 | 0,16816 |
| RPTOR | rs199810207 | 78947036 | normSNP | 0 | A/C | 98,9 | 0,016949 | 1 | - | - | - | - |
| RPTOR | rs6565508 | 78948554 | normSNP | 0 | A/G | 66,5 | 0,475184 | 0,12816 | 0,05101 | 1 | 0,05409 | 0,12866 |

**^1^**according to GRCh37/hg19.

**^2^**ecreSNP (disrupts CRE and is eQTL), creSNP (disrupts CRE, not an eQTL), eSNP (only an eQTL)

**^3^**LD with the variant of the original predictor for each gene

**^4^**Calculated with the 88 samples of the Discovery cohort that where resequenced

**^5^**p-value of each model (codominant, dominant, recessive, overdominant, logg-additive) in the preliminary association test

**Table S2.** Summary of the primers and restriction enzymes used for cloning and for mutagenesis of each SNP tested in Luciferase Reporter Assays.

| Gene | Identifier | Location | SNP | A1 | A2 | 5´ | 3´ | Primer sequence: cloning | Mutagenesis | Primer sequence:  mutagenesis |
| --- | --- | --- | --- | --- | --- | --- | --- | --- | --- | --- |
| *AKT1* | 1 | chr14:105259500-105261800 | rs1130214* | C | A | Sac I | Hind III | F-CGAGCTCGCCGCCCGGGCCTGGATTTCC  R-CCCAAGCTTGCCCCTTTGACTTCTTTGACCCAG | C to A | F-GAGGTTTTTGTGCTTGCGCTGG  R-CTGGGAGAAACCCCAGGC |
| *AKT1* | 2 | chr14:105262088-105263088 | rs74090038* | C | T | Kpn I | Hind III | F-CGGGGTACCCTGAAAATAAAAATGCTCCCCCAACC  R-CCCAAGCTTGGCGGAGCGCGCTCGGGGAGC | C to T | F-gggagggctctggactgtcagctgtca  R-tgacagctgacagtccagagccctccc |
| *AKT1* | 4 | chr14:105271000-105272088 | rs33925946* | G | T | Kpn I | Xho I | F-CGGGGTACCGCCCACCCTGGCCAGGAGTGG  R-CCGCTCGAGTCCTCTTTTCACATCATGGCGACAG | G to T | F-cctgcaggacagggaactggtccaaactc  R-gagtttggaccagttccctgtcctgcagg |
| *FCHSD1* | 1 | chr5:141015900-141017000 | rs1421896 | T | G | Kpn I | Hind III | F-*GGGGTACCAGGACCAGGGGGCGGAGCGCAG*  R-CCCAAGCTTAGAGGGTGGCCGACCTGAGGTG | G to T | F-cggtgaggaaacagggatatgcatgaggggttg  R-caacccctcatgcatatccctgtttcctcaccg |
| *FCHSD1* | 2 | chr5:141036800-141038500 | rs34798770 | G | A | Xho I | Hind III | F-CCGCTCGAGTTATTCAAGGTCACCCTGTAGCGAG  R-CCCAAGCTTTGGTAGAAGATTTCAAGCATGACAAAATG | G to A | F-actccaaatgttggagagacccatggctcaatccta  R-taggattgagccatgggtctctccaacatttggagt |
| *DDIT4* | 1 | chr10:74034943-74035797 | rs1053639 | T | A | Kpn I | Hind III | F-CCCGGTACCTTGAACTTCAACCTGAGGGGGCCG  R-CCCAAGCTTAACTGTTTTAACAAACATGTTTATTAGAAAAG | T to A | F-gctgagggactgattccagtggttggaaaactgag  R-ctcagttttccaaccactggaatcagtccctcagc |
| *DDIT4* | 2 | chr10:74035797-74036678 | rs4747241 | C | T | Kpn I | Hind III | F-CCCGGTACCTGGTTGAGTCTCTCATCAGTACCATG  R-CCCAAGCTTTGGATGTAATTCCAGGGAAGAAGG | T to C | F-atagcaggcctaccttaacgataccttgggtcac  R-gtgacccaaggtatcgttaaggtaggcctgctat |
| *DDIT4* | 3 | chr10:74036797-74037678 | rs4747242 | A | C | Kpn I | Xho I | F-CCCGGTACCCATCAGGGATTCCAATAGAGGGTCC  R-CCGCTCGAGCTGTGAGACTCAAGCTCAGGGCTC | C to A | F-gtccactcccagcctattgagattcagggct  R-agccctgaatctcaataggctgggagtggac |
| *DDIT4* | 4 | chr10:74039870-74040480 | rs10823911 | A | C | Kpn I | Xho I | F-CCCGGTACCTCTACTAAAAATAGACAAATTTGCCAAGC  R-CCGCTCGAGCTGCATCTGCTGCCCCAGAAGTTTC | C to A | F-ggcaacagattgagactctgtttaaaaaaaaaaaattcctttaattccacc  R-ggtggaattaaaggaattttttttttttaaacagagtctcaatctgttgcc |
| *RPTOR* | 2 | chr17:78578583-78580283 | rs9899898 | G | A | Xho I | Bgl II | F-CCGCTCGAGTTATTCACCATCATGAGAACAGCACAG  R-GGAAGATCTTTCAAAAGCTAACGGAAACCTCCTAAAAAG | A to G | F-ttttccgttactgtgtccgtgatgagttgaggaaacc  R-ggtttcctcaactcatcacggacacagtaacggaaaa |
| *RPTOR* | 3 | chr17:78753340-78756340 | rs9915667 | A | G | Xho I | Hind III | F-CCGCTCGAGTGTGAACATTGAGATGTGCAATTCCG  R-CCCAAGCTTTGCAAGAGGCATTAGTTTCCCGCTAAATAC | A to G | F-gggctagaaggggattggtggaggacatctc  R-gagatgtcctccaccaatccccttctagccc |
| *CMV* | - | - | - | - | - | Kpn I | Hind III | F-CGGGGTACCTAGTTATTAATAGTAATCAATTACGGGG  R-CCCAAGCTTGATCTGACGGTTCACTAAACCAGC | - | - |
